# Revealing the Role of the Cyaphide Ion as a Bridging Ligand in Heterometallic Complexes

**DOI:** 10.1002/anie.202206783

**Published:** 2022-07-06

**Authors:** Eric S. Yang, Jose M. Goicoechea

**Affiliations:** ^1^ Department of Chemistry University of Oxford Chemistry Research Laboratory 12 Mansfield Road Oxford OX1 3TA U.K.

**Keywords:** Coordination Chemistry, Cyaphide, π-Interactions, Structure and Bonding, Transition Metals

## Abstract

The synthesis of heterometallic transition metal complexes featuring bridging cyaphide ions (C≡P^−^) is reported. These are synthesized from reactions of Au(IDipp)(CP) (IDipp=1,3‐bis(2,6‐diisopropylphenyl)imidazol‐2‐ylidene) with electron‐rich, nucleophilic transition metal reagents, affording Au(IDipp)(μ_2_−C≡P)Ni(^Me^I^*i*^Pr)_2_ (^Me^I^*i*^Pr=1,3‐diisopropyl‐4,5‐dimethylimidazol‐2‐ylidene) and Au(IDipp)(μ_2_−C≡P)Rh(Cp*)(PMe_3_). These studies reveal that, in contrast to the cyanide ion, bimetallic cyaphido complexes strongly favor a η^1^ : η^2^ coordination mode that maximizes the interaction of the second metal (Ni, Rh) with the π‐manifold of the ion (and not the phosphorus atom lone pair). End‐on bridging can be effectively unlocked by blocking the π‐manifold, as demonstrated by reaction of Au(IDipp)(μ_2_−C≡P)Rh(Cp*)(PMe_3_) with an electrophilic transition metal reagent, W(CO)_5_(THF), which affords the heterotrimetallic compound Au(IDipp)(μ_3_−C≡P)[Rh(Cp*)(PMe_3_)][W(CO)_5_].

## Graphical abstract

Hetero bi‐ and tri‐metallic transition metal complexes featuring bridging cyaphide ions (C≡P^−^) are reported. These were synthesized from reactions of Au(IDipp)(CP) with electron‐rich, nucleophilic transition metal reagents. These studies suggest that the reactivity of Au(IDipp)(CP) is largely LUMO‐driven and that it behaves as a metallo‐phosphaalkyne.

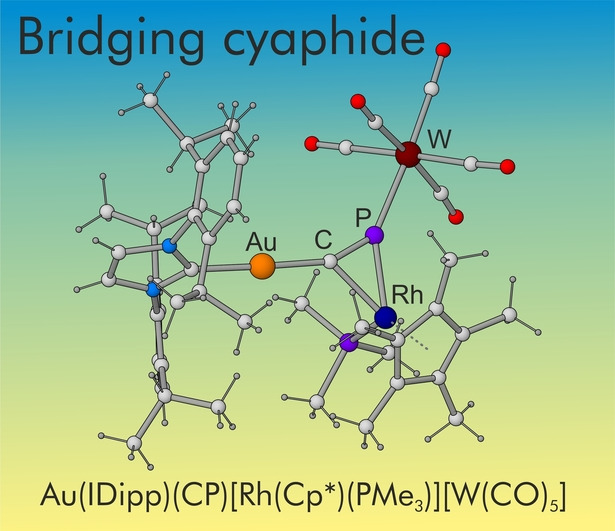

Since the landmark synthesis of Prussian Blue by Diesbach in the early 1700s, the cyanide ion has found use as a building block in inorganic and materials chemistry.^[1]^ Today, the cyanide ion is still ubiquitously used as a ligand in technologically relevant molecules and solids, particularly Prussian Blue Analogues (PBAs), which are of interest due to their stimulus‐responsive magnetic properties.[^2^, ^3^, ^4^, ^5^] Similarly, organic compounds containing a cyanido group such as nitriles (R−C≡N) and isonitriles (R−N≡C) are important species which, among their many applications, are also commonly used as ligands in coordination chemistry.^[6]^ Both nitriles and κ‐N cyanido complexes overwhelmingly prefer to coordinate to Lewis acids through an end‐on η^1^ coordination mode that is driven by σ‐donation from the nitrogen lone pair (the HOMO of the CN^−^ ion). In bimetallic compounds, the cyanide ligand typically binds terminally between two metal centers as depicted in Figure 1, in what we will refer to as a η^1^ : η^1^ (or σ+σ) interaction.^[7]^

**Figure 1:**
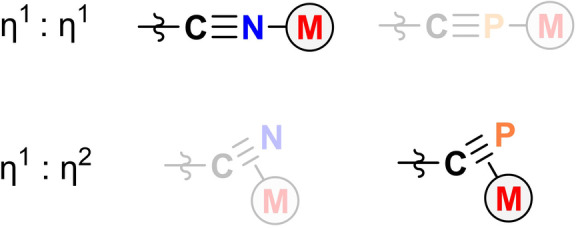
Dominant coordination modes for bridging cyanides, nitriles, and phosphaalkynes.

The valence isoelectronic heavier analogue of cyanide, the cyaphide ion (C≡P^−^), is more elusive. Only a handful of metal cyaphido complexes have been reported, the vast majority of which exhibit terminal, κ*C* coordination (i.e. [M]−C≡P).[^8^, ^9^, ^10^, ^11^, ^12^, ^13^] The dearth of known cyaphido complexes is largely due to the fact that, until recently, there was not a widely applicable route available for their synthesis. Heavier analogues of nitriles, so‐called phosphaalkynes (R−C≡P), have been studied much more widely.[^14^, ^15^] In contrast to cyanides and nitriles, phosphaalkynes usually bind to metals in a side‐on, η^2^ coordination mode, as is also observed for valence isoelectronic alkynes (Figure 1).

Molecules containing the cyaphide ion as a bridging ligand between metal centers are an attractive target. In particular, they could be used to tune electronic communication pathways in heterobimetallic complexes. The substitution of nitrogen for phosphorus in [M]−C≡X−[M] type compounds (X=N, P) should, in principle, allow for enhanced π back‐bonding, a feature which has shown to play an important role in bimetallic complexes with bridging cyanido ligands.^[16]^ However, to date, the isolation of multimetallic μ‐cyaphido complexes has proven to be a significant challenge. Despite the successful synthesis of several novel transition metal cyaphide complexes in the last two decades, the original platinum(II)/platinum(0) cyaphido complex reported by Angelici in 1992 remains the only example where the cyaphide ion behaves a bridging ligand.^[8]^ It bears mentioning at this stage that this homobimetallic platinum compound contains a η^1^ : η^2^ cyaphido ligand (σ+π).^[7]^

We recently reported the synthesis of a linear gold(I) cyaphido complex, Au(IDipp)(C≡P) (**A**), prepared by salt metathesis using a magnesium(II) cyaphide transfer reagent.^[17]^ Herein, we show that **A** can be used as a precursor to hetero‐ bi‐ and tri‐metallic transition metal complexes featuring the cyaphide ion as a bridging ligand in η^1^ : η^2^ (σ+π) and η^1^ : η^2^ : η^1^ (σ+π+σ) coordination modes.

We reasoned that the minimal steric protection of the cyaphido ligand in the linear gold(I) complex **A** would allow for reactivity at the cyaphide phosphorus lone pair. However, reactions of **A** with coordinatively unsaturated electrophiles (e.g. Mo(depe)_2_(N_2_)_2_, W(CO)_5_(THF), and B(C_6_F_5_)_3_; depe=1,2‐bis(diethylphosphino)ethane) were unsuccessful, resulting in decomposition to intractable product mixtures (as determined by ^31^P{^1^H} NMR spectroscopy). By comparison, the isostructural gold(I) cyanide complex, Au(IDipp)(CN) (**B**),^[18]^ reacts readily with B(C_6_F_5_)_3_ to afford the bridging η^1^ : η^1^‐cyanido complex Au(IDipp)(μ_2_‐CN)B(C_6_F_5_)_3_ (**1**), as depicted in Scheme 1 (see Supporting Information for further details). Structural authentication of **1** by single‐crystal X‐ray diffraction confirms that the compound contains a bridging η^1^ : η^1^‐cyanido ligand with C‐Au‐C, Au‐C‐N and C‐N‐B angles of 176.3(1), 174.1(2) and 175.7(3)°, respectively.^[19]^

**Scheme 1:**
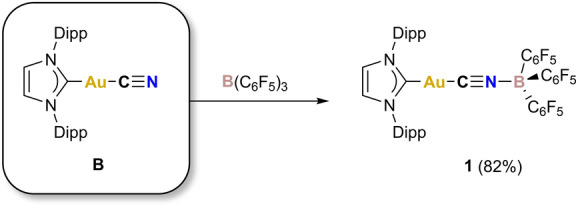
Synthesis of **1**.

Due to the lack of success in forming multi‐metallic complexes from the reaction of **A** with metal *electrophiles*, the umpolung reaction with electron‐rich, *nucleophilic* metals was attempted. We hypothesized that the increased electrophilicity of **A** (driven by energetically accessible π* orbitals) might allow access to bimetallic compounds via LUMO‐driven reactivity (see Supporting Information for computational details on the electronic structures of **A** and **B**).

For this purpose, we chose to study the reactivity of **A** towards electron‐rich late transition metal complexes. Nickel(0) centers are known to form complexes with electrophilic π‐acceptor ligands such as alkynes and phosphaalkynes;[^20^, ^21^] thus we envisioned that the nickel(0) carbene complex Ni(^Me^I^*i*^Pr)_2_(COD)_*x*_ could be used to prepare a bridging cyaphide complex.^[22]^ Ni(^Me^I^*i*^Pr)_2_(COD)_0.7_ [prepared as a mixture of Ni(^Me^I^*i*^Pr)_2_(COD) and {Ni(^Me^I^*i*^Pr)_2_}_2_(μ_2_‐COD)] reacts with **A** at room temperature, resulting in the displacement of COD and immediate, quantitative formation of the heterobimetallic compound Au(IDipp)(μ_2_‐C≡P)Ni(^Me^I^*i*^Pr)_2_ (**2**; Scheme 2).

**Scheme 2:**
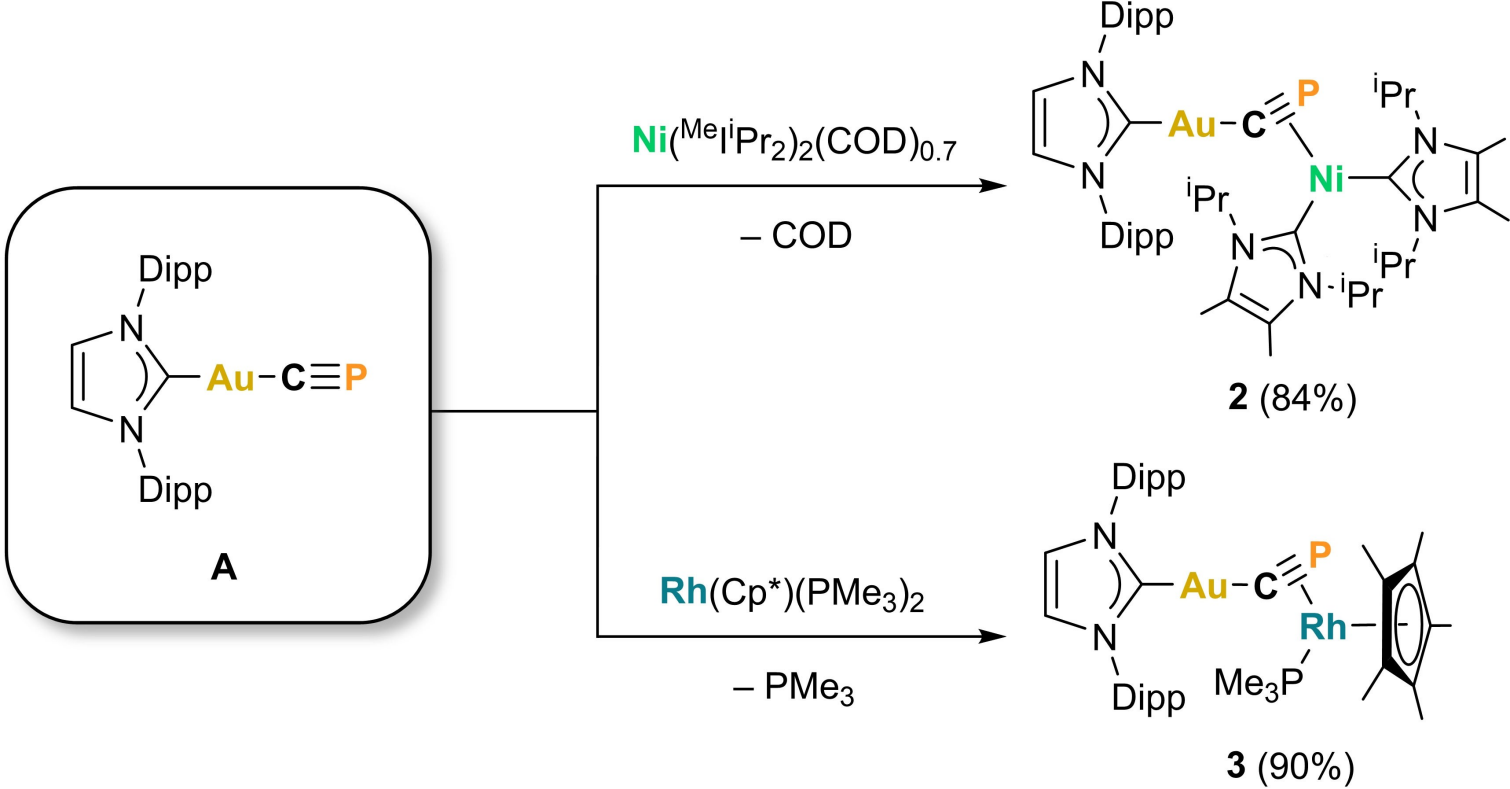
Reactivity of **A** toward electron‐rich transition metals.

Compound **2** exhibits a single resonance in its ^31^P{^1^H} NMR spectrum at 246.0 ppm, as well as a doublet in its ^13^C{^1^H} spectrum at 280.3 ppm (^1^ *J*_C−P_=105 Hz) corresponding to the μ_2_‐cyaphido carbon. The ^1^H NMR spectrum of **2** exhibits two sets of signals for two inequivalent ^Me^I^*i*^Pr carbene ligands on nickel. The C≡P stretching frequency was measured to be 1125 cm^−1^ by dispersive Raman spectroscopy (Figure S8), significantly lower than the Raman C≡P stretch for **A** (1350 cm^−1^; Figure S21), and indicative of a weakening of the C≡P bond upon coordination to a second metal. Crystals of **2** were obtained from diffusion of hexane into a concentrated benzene solution, allowing its structure to be confirmed by single crystal X‐ray diffraction (Figure 2). The solid‐state structure of **2** reveals η^1^ : η^2^ coordination of the bridging cyaphide ligand, with a bent Au1‐C1‐P1 bond angle of 146.3(2)° (cf. **A**: 178.0(4)°) and Au1−C1 bond length of 1.992(4) Å (cf. **A**: 1.972(6) Å). The nickel binds to the cyaphide ion closer to the carbon atom with a Ni1−C1 bond length of 1.919(4) Å and Ni1−P1 bond length of 2.201(1) Å. The C1−P1 bond length is 1.642(4) Å, lying between the predicted values for carbon‐phosphorus triple (1.54 Å) and double bonds (1.69 Å);^[23]^ in comparison to **A**, it is significantly elongated (**A**: 1.552(6) Å), in line with its reduced vibrational frequency. DFT calculations (ZORA‐ωB97X‐D3/{ZORA‐def2‐TZVP, SARC‐ZORA‐TZVP(Au)}) show that the η^1^ : η^2^‐cyaphido coordination mode of **2** is 29.3 kcal mol^−1^ lower in energy than the η^1^ : η^1^‐isomer (S32).

**Figure 2:**
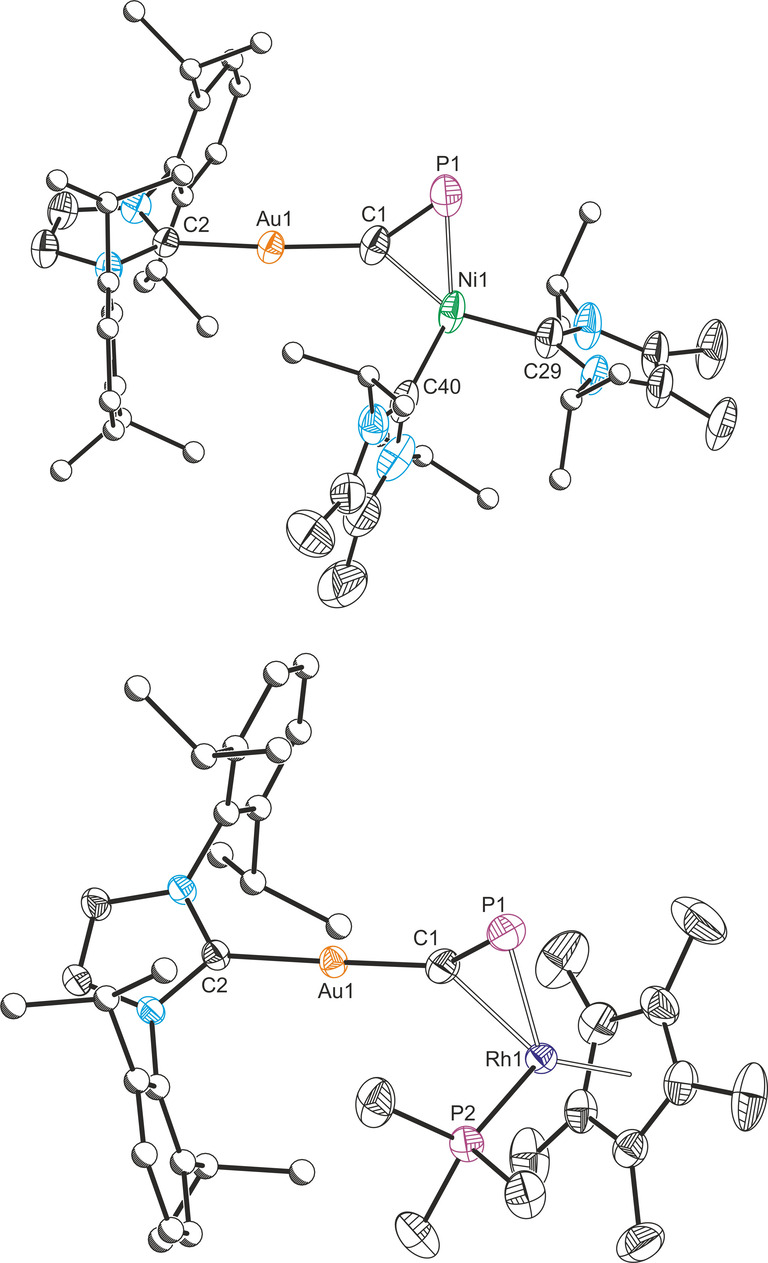
Solid‐state structures of **2** (top) and **3** (bottom). Anisotropic displacement ellipsoids set at 50 % probability. Hydrogen atoms omitted for clarity. Carbon atoms of Dipp and ^*i*^Pr groups displayed as spheres of arbitrary radius. Selected bond lengths [Å] and angles [°] **2**: Au1−C1 1.992(4), Au1−C2 2.024(3), C1−P1 1.642(4), C1−Ni1 1.919(3), P1−Ni1 2.2013(14), Ni1−C29 1.920(3), Ni1−C40 1.922(4); C1‐Au1‐C2 175.62(13), Au1‐C1‐P1 146.3(2), C1‐Ni1‐P1 46.34(12); **3**: Au1−C1 1.976(4), Au1−C2 2.026(4), C1−P1 1.631(4), C1−Rh1 2.119(4), P1−Rh1 2.371(2), Rh1−P2 2.209(1), Rh1−Cp*(cent.) 1.911; C1‐Au1‐C2 174.52(16), Au1‐C1‐P1 151.4(3), C1‐Rh1‐P1 42.12(12).

Compound **2** shows that the cyaphide ligand is capable of coordinating to electron‐rich metals through its π‐system. Encouraged by this finding, we sought to apply this protocol to another transition metal. The rhodium(I) piano‐stool complex Rh(Cp*)(PMe_3_)_2_ is known to react as a metal‐centered Lewis base,^[24]^ therefore we reasoned that it too would be an appropriate precursor to bridging cyaphido complexes. Heating a mixture of **A** and Rh(Cp*)(PMe_3_)_2_ at 80 °C overnight results in the liberation of trimethylphosphine (observed by ^31^P{^1^H} NMR spectroscopy), and the formation of the gold(I)/rhodium(I) bridging cyaphide complex Au(IDipp)(μ_2_−C≡P)Rh(Cp*)(PMe_3_) (**3**; Scheme 2). **3** exhibits two resonances in its ^31^P{^1^H} NMR spectrum, a doublet of doublets at 94.7 ppm (^1^ *J*_P−Rh_=34 Hz, ^2^ *J*_P−P_=5 Hz) and a doublet of doublets at −5.0 ppm (^1^ *J*_P−Rh_=209 Hz, ^2^ *J*_P−P_=5 Hz), corresponding to the μ_2_‐cyaphido ligand and trimethylphosphino ligand, respectively. The dispersive Raman spectrum shows a C≡P bond stretching frequency of 1175 cm^−1^ (Figure S13), slightly higher than for **2**, but once again much lower than the non‐bridging C≡P stretch in **A**. **3** can be crystallized as orange blocks from slow evaporation of hexane solutions at room temperature. X‐ray diffraction reveals the same η^1^ : η^2^ cyaphide coordination mode as **2** (Figure 2). The Au1‐C1‐P1 bond angle is slightly larger than **2**, at 151.4(2)°, and the Au1−C1 bond length is 1.975(4) Å. The Rh1−C1 bond length is 2.119(4) Å and the Rh1−P1 bond length is 2.371(1) Å. As with **2**, the C1−P1 bond length is elongated (1.631(4) Å), in line with the lower frequency of the cyaphide stretching mode.

The crystal structures of **2** and **3** reveal that the cyaphide phosphorus atom should still be sterically accessible for further reactivity with electrophiles. This was probed by adding **3** to W(CO)_5_(THF),^[25]^ which immediately resulted in a color change from bright yellow to red and formation of the heterotrimetallic complex Au(IDipp)(μ_3_‐C≡P)[Rh(Cp*)(PMe_3_)][W(CO)_5_] (**4**; Figure 3). An analogous reaction between **2** and W(CO)_5_(THF) gave rise to an intractable mixture of products as determined by ^31^P NMR spectroscopy. The ^31^P{^1^H} NMR spectrum of **4** reveals a doublet of doublets with ^183^W satellites (48.5 ppm, ^1^ *J*_P−W_=175 Hz, ^1^ *J*_P−Rh_=62 Hz, ^2^ *J*_P−P_=13 Hz) corresponding to the μ_3_‐cyaphido ligand, and another doublet of doublets at −3.9 ppm (^1^ *J*_P−Rh_=194 Hz, ^2^ *J*_P−P_=13 Hz) arising from the trimethylphosphine ligand. The IR spectrum shows a set of bands between 1881 and 2059 cm^−1^ corresponding to carbonyl stretching modes (Figure S18), several of which are also visible in the Raman spectrum between 1883 and 2062 cm^−1^ (Figure S19). The C≡P stretching mode appears at 1186 cm^−1^ in the Raman spectrum, slightly higher than that of **3**. Red crystals of **4** were obtained from a concentrated hexane solution at 5 °C, allowing for the determination of its structure by X‐ray crystallography. The cyaphide ion in **4** bridges the three metal centers in a η^1^ : η^2^ : η^1^ (σ+π+σ) coordination mode (Figure 3 inset). The Rh1−C1 distance is 2.159(4) Å and the Rh1−P1 distance is 2.297(2) Å, representing a slight shift of the rhodium centre closer to phosphorus atom. The C1−P1 bond length (1.605(4) Å), is slightly shorter than that of **3** (1.631(4) Å). This is in line with the moderate increase of the ν_CP_ stretching frequency on coordinating the W(CO)_5_ moiety to the phosphorus lone pair. These data suggest an increase in bond order on going from **3** to **4**, which is consistent with an increase in the polarization of the C−P bond. This can be observed in the increase of the ^31^P−^103^Rh coupling constant of the cyaphide phosphorus atom on coordination of W(CO)_5_ (34 to 62 Hz).

**Figure 3:**
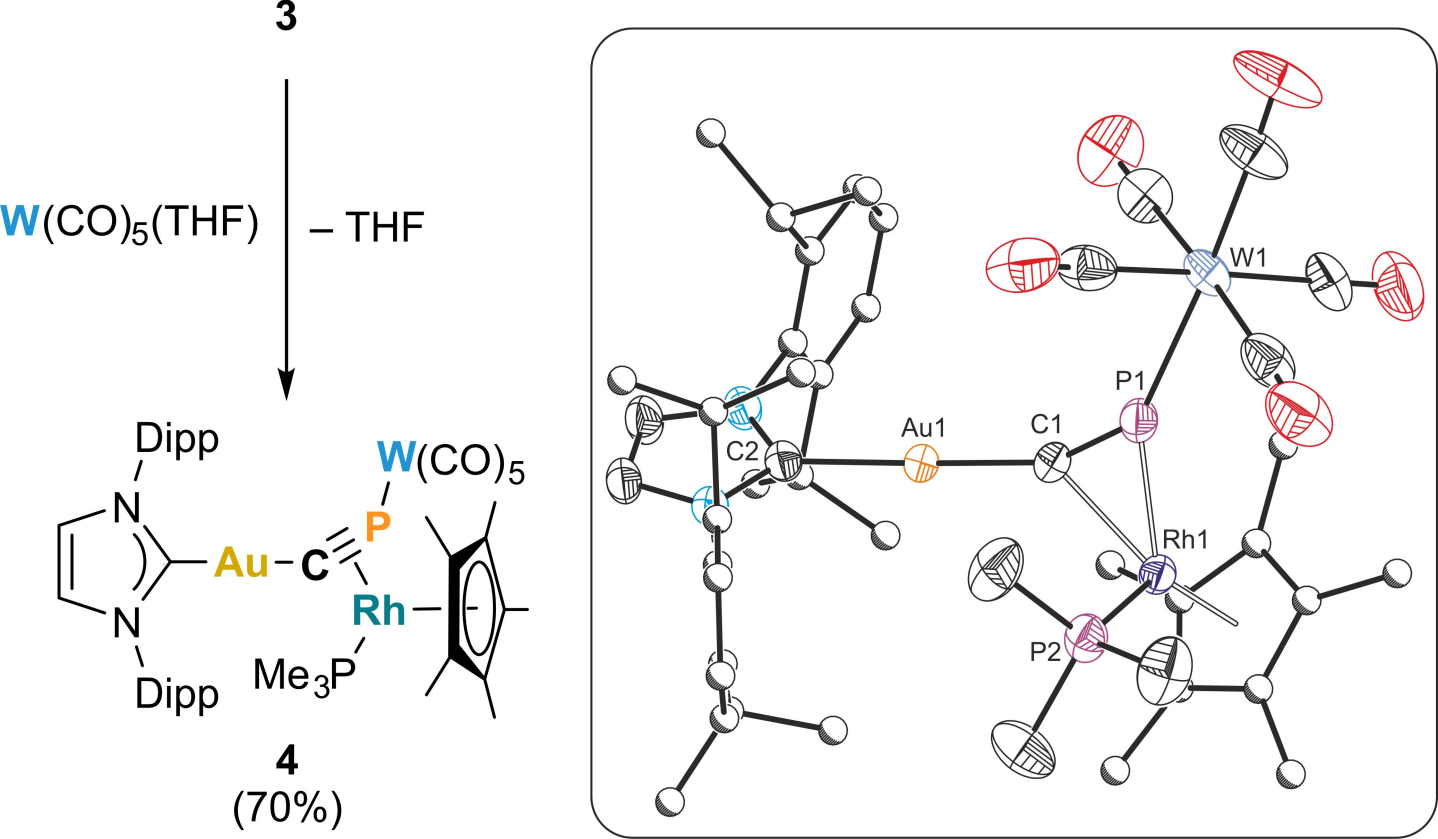
Synthesis of the heterotrimetallic bridging cyaphide complex **4** (left). Solid‐state structure of **4** (right). Anisotropic displacement ellipsoids set at 50 % probability. Hydrogens omitted for clarity. Carbon atoms of Dipp and Cp* groups displayed as spheres of arbitrary radius. Selected bond lengths [Å] and angles [°]: Au1−C1 1.991(4), Au1−C2 2.025(4), C1−P1 1.605(4), C1−Rh1 2.159(4), P1−Rh1 2.297(2), P1−W1 2.499(1), Rh1−P2 2.233(2), Rh1−Cp*(cent.) 1.903; C1‐Au1‐C2 174.7(2), Au1‐C1‐P1 155.4(3), C1‐P1‐W1 144.57(16), C1‐Rh1‐P1 42.09(11).

In order to better understand the electronic structure of the bridging cyaphido ligand, **2**–**4** were investigated computationally at the ZORA‐ωB97X‐D3/{ZORA‐def2‐TZVP, SARC‐ZORA‐TZVP(Rh, W, Au)} level of theory (see the Supporting Information for further details).[^26^, ^27^, ^28^] A fragment‐based analysis was performed to deconvolute the M−(η^2^−C≡P) bonding interactions. Using the ETS‐NOCV energy decomposition scheme,[^29^, ^30^] the orbital contributions to the total interaction energy of each M−(η^2^−C≡P) bond were separated into σ donation and π back‐donation components. For all three complexes **2**–**4**, the largest contribution to the M−(η^2^−C≡P) bond was found to be π back‐donation from Ni/Rh into a cyaphide π* antibonding orbital (Figure 4). In all three cases, a secondary contribution of σ donation from a cyaphide π bonding orbital to Ni/Rh was found to be the only other major interaction. This shows that the cyaphide ion behaves primarily as a π acceptor ligand in the side‐on η^2^ coordination mode. The degree of computed π back‐bonding character in **2**–**4** correlates well with observed trends in their solid‐state bond metrics and vibrational spectra (Figure S27–S29). **2**, which exhibits the longest C−P bond length, smallest Au‐C‐P bond angle, and lowest C≡P stretching frequency, was calculated to have the highest degree of π back‐bonding to the cyaphide ligand.

**Figure 4:**
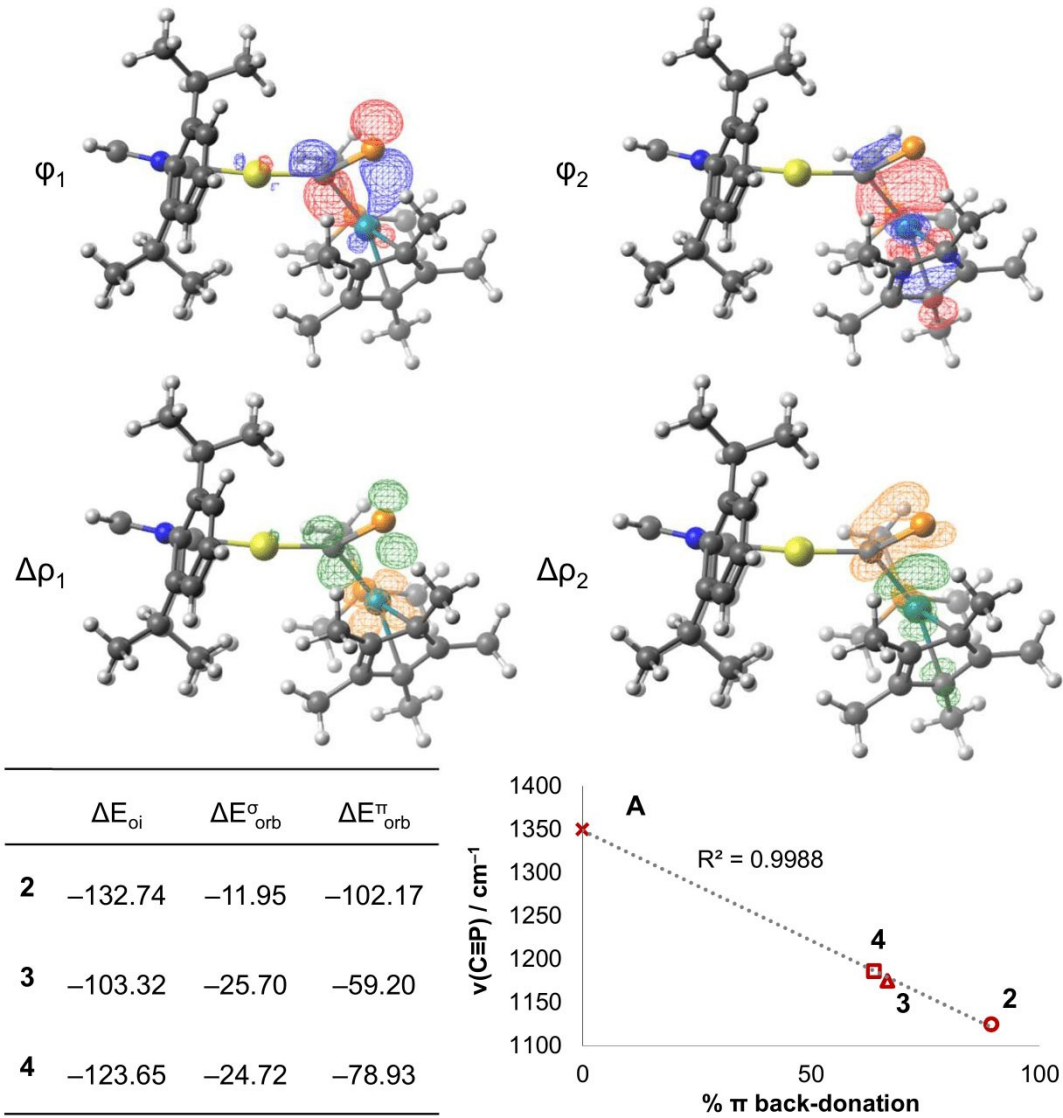
Isosurface plots of the first two NOCVs (ϕ_1_ and ϕ_2_, ν>0) of **3**, and their difference densities (Δ*ρ*_1_ and Δ*ρ*_2_, green: Δ*ρ*>0; orange: Δ*ρ*<0), corresponding to π back‐donation and σ donation, respectively (top). ETS‐NOCV analysis of the orbital interaction contribution to the M−(η^2^−C≡P) bonds in **2**–**4**, with energies in kcal mol^−1^ (bottom left). Correlation of calculated π back‐donation character in **A**, **2**, **3**, and **4** with their C≡P vibrational frequencies (bottom right).

We have shown that the cyaphide ion can be used as a bridging ligand in multimetallic transition metal complexes, and have demonstrated that such systems are accessible through the reaction of a terminal cyaphide complex with electron‐rich metal precursors. This reactivity reflects the preference of the cyaphide ion to act as a π‐acceptor in the side‐on η^2^ coordination mode, in sharp contrast to the end‐on η^1^ coordination typically observed for its lighter congener, cyanide. This undoubtedly has implications for prospective cyaphide‐containing materials, for example PBAs, for which the nature of bridging cyaphido ligands will have a profound impact on their structure and properties.

## Conflict of interest

The authors declare no conflict of interest.

## Supporting information

As a service to our authors and readers, this journal provides supporting information supplied by the authors. Such materials are peer reviewed and may be re‐organized for online delivery, but are not copy‐edited or typeset. Technical support issues arising from supporting information (other than missing files) should be addressed to the authors.

Supporting Information

Supporting Information

Supporting Information

## Data Availability

The data that support the findings of this study are available in the Supporting Information of this article.
