# Supplementary material for: Revealing the Role of the Cyaphide Ion as a Bridging Ligand in Heterometallic Complexes
Source: Angew Chem Int Ed Engl. 2022 Jul 6;61(33):e202206783. doi: 10.1002/anie.202206783 (PMC9546431; doi:10.1002/anie.202206783)

checkCIF/PLATON report

You have not supplied any structure factors. As a result the full set of tests cannot be run.

THIS REPORT IS FOR GUIDANCE ONLY. IF USED AS PART OF A REVIEW PROCEDURE FOR PUBLICATION, IT SHOULD NOT REPLACE THE EXPERTISE OF AN EXPERIENCED CRYSTALLOGRAPHIC REFEREE.

No syntax errors found. CIF dictionary Interpreting this report

Datablock: ESY041_2

Bond precision: C-C = 0.0065 A Wavelength=1.54184

Cell:	a=12.9001(2)	b=14.2097(2)	c=16.5212(2)
	alpha=67.537(1)	beta=83.160(1)	gamma=64.777(1)
Temperature:	150 K		

	Calculated	Reported
Volume	2528.36(7)	2528.37(7)
Space group	P -1	P -1
Hall group	-P 1	-P 1
Moiety formula	C46 H36 Au B F15 N3, C7 H8	C46 H36 Au B F15 N3, C7 H8
Sum formula	C53 H44 Au B F15 N3	C53 H44 Au B F15 N3
Mr	1215.69	1215.69
Dx,g cm-3	1.597	1.597
Z	2	2
Mu (mm-1)	6.306	6.306
F000	1204.0	1204.0
F000'	1199.36	
h,k,lmax	16,17,20	16,17,20
Nref	10602	10499
Tmin,Tmax	0.312,0.321	0.858,1.000
Tmin'	0.200	

Correction method= # Reported T Limits: Tmin=0.858 Tmax=1.000
AbsCorr = MULTI-SCAN

Data completeness= 0.990 Theta(max)= 76.263

R(reflections)= 0.0253(10407) wR2(reflections)= 0.0618(10499)

S = 1.040 Npar= 718

The following ALERTS were generated. Each ALERT has the format
test-name_ALERT_alert-type_alert-level.
Click on the hyperlinks for more details of the test.

Alert level B

```
PLAT220_ALERT_2_B NonSolvent   Resd 1  C   Ueq(max)/Ueq(min) Range        6.2 Ratio
PLAT230_ALERT_2_B Hirshfeld Test Diff for    C8        --C9       .      15.2 s.u.
PLAT250_ALERT_2_B Large U3/U1 Ratio for Average U(i,j) Tensor ....        4.2 Note
PLAT331_ALERT_2_B Small Aver Phenyl C-C Dist C2T       --C7T      .      1.36 Ang.
```

Alert level C

```
PLAT213_ALERT_2_C Atom C15             has ADP max/min Ratio .....        3.1 prolat
PLAT222_ALERT_3_C NonSolvent Resd 1  H   Uiso(max)/Uiso(min) Range        4.4 Ratio
PLAT223_ALERT_4_C Solv./Anion  Resd 3  H   Ueq(max)/Ueq(min) Range        4.5 Ratio
PLAT230_ALERT_2_C Hirshfeld Test Diff for    C9        --C10      .       5.5 s.u.
PLAT230_ALERT_2_C Hirshfeld Test Diff for    C18       --C23      .       6.5 s.u.
PLAT230_ALERT_2_C Hirshfeld Test Diff for    C19       --C20      .       6.8 s.u.
PLAT230_ALERT_2_C Hirshfeld Test Diff for    C22       --C26      .       5.7 s.u.
PLAT241_ALERT_2_C High   'MainMol' Ueq as Compared to Neighbors of         C9 Check
PLAT242_ALERT_2_C Low    'MainMol' Ueq as Compared to Neighbors of        C11 Check
PLAT242_ALERT_2_C Low    'MainMol' Ueq as Compared to Neighbors of        C14 Check
PLAT242_ALERT_2_C Low    'MainMol' Ueq as Compared to Neighbors of        C23 Check
PLAT242_ALERT_2_C Low    'MainMol' Ueq as Compared to Neighbors of        C26 Check
PLAT250_ALERT_2_C Large U3/U1 Ratio for Average U(i,j) Tensor ....        3.7 Note
PLAT260_ALERT_2_C Large Average Ueq of Residue Including      C1S      0.107 Check
PLAT260_ALERT_2_C Large Average Ueq of Residue Including      C1T      0.118 Check
PLAT331_ALERT_2_C Small Aver Phenyl C-C Dist C2S       --C7S      .      1.37 Ang.
```

Alert level G

```
PLAT002_ALERT_2_G Number of Distance or Angle Restraints on AtSite         14 Note
PLAT154_ALERT_1_G The s.u.'s on the Cell Angles are Equal ..(Note)      0.001 Degree
PLAT175_ALERT_4_G The CIF-Embedded .res File Contains SAME Records          1 Report
PLAT232_ALERT_2_G Hirshfeld Test Diff (M-X)  Au1       --C1       .       6.9 s.u.
PLAT300_ALERT_4_G Atom Site Occupancy of C1S       Constrained at         0.5 Check
PLAT300_ALERT_4_G Atom Site Occupancy of C2S       Constrained at         0.5 Check
PLAT300_ALERT_4_G Atom Site Occupancy of C3S       Constrained at         0.5 Check
PLAT300_ALERT_4_G Atom Site Occupancy of C4S       Constrained at         0.5 Check
PLAT300_ALERT_4_G Atom Site Occupancy of C5S       Constrained at         0.5 Check
PLAT300_ALERT_4_G Atom Site Occupancy of C6S       Constrained at         0.5 Check
PLAT300_ALERT_4_G Atom Site Occupancy of C7S       Constrained at         0.5 Check
PLAT300_ALERT_4_G Atom Site Occupancy of H1S1      Constrained at         0.5 Check
PLAT300_ALERT_4_G Atom Site Occupancy of H1S2      Constrained at         0.5 Check
PLAT300_ALERT_4_G Atom Site Occupancy of H1S3      Constrained at         0.5 Check
PLAT300_ALERT_4_G Atom Site Occupancy of H3S       Constrained at         0.5 Check
PLAT300_ALERT_4_G Atom Site Occupancy of H4S       Constrained at         0.5 Check
PLAT300_ALERT_4_G Atom Site Occupancy of H5S       Constrained at         0.5 Check
PLAT300_ALERT_4_G Atom Site Occupancy of H6S       Constrained at         0.5 Check
PLAT300_ALERT_4_G Atom Site Occupancy of H7S       Constrained at         0.5 Check
PLAT300_ALERT_4_G Atom Site Occupancy of C1T       Constrained at         0.5 Check
PLAT300_ALERT_4_G Atom Site Occupancy of C2T       Constrained at         0.5 Check
PLAT300_ALERT_4_G Atom Site Occupancy of C3T       Constrained at         0.5 Check
PLAT300_ALERT_4_G Atom Site Occupancy of C4T       Constrained at         0.5 Check
```

```
PLAT300_ALERT_4_G Atom Site Occupancy of C5T        Constrained at        0.5 Check
PLAT300_ALERT_4_G Atom Site Occupancy of C6T        Constrained at        0.5 Check
PLAT300_ALERT_4_G Atom Site Occupancy of C7T        Constrained at        0.5 Check
PLAT300_ALERT_4_G Atom Site Occupancy of H3T        Constrained at        0.5 Check
PLAT300_ALERT_4_G Atom Site Occupancy of H1T1       Constrained at        0.5 Check
PLAT300_ALERT_4_G Atom Site Occupancy of H4T        Constrained at        0.5 Check
PLAT300_ALERT_4_G Atom Site Occupancy of H1T2       Constrained at        0.5 Check
PLAT300_ALERT_4_G Atom Site Occupancy of H5T        Constrained at        0.5 Check
PLAT300_ALERT_4_G Atom Site Occupancy of H1T3       Constrained at        0.5 Check
PLAT300_ALERT_4_G Atom Site Occupancy of H6T        Constrained at        0.5 Check
PLAT300_ALERT_4_G Atom Site Occupancy of H7T        Constrained at        0.5 Check
PLAT302_ALERT_4_G Anion/Solvent/Minor-Residue Disorder (Resd  2  )       100% Note
PLAT302_ALERT_4_G Anion/Solvent/Minor-Residue Disorder (Resd  3  )       100% Note
PLAT304_ALERT_4_G Non-Integer Number of Atoms in ..... (Resd  2  )       7.50 Check
PLAT304_ALERT_4_G Non-Integer Number of Atoms in ..... (Resd  3  )       7.50 Check
PLAT380_ALERT_4_G Incorrectly? Oriented X(sp2)-Methyl Moiety .....        C1S Check
PLAT380_ALERT_4_G Incorrectly? Oriented X(sp2)-Methyl Moiety .....        C1T Check
PLAT413_ALERT_2_G Short Inter XH3 .. XHn     H1S1     ..H28C     .       2.06 Ang.
                                                  -x,1-y,-z  =      2_565 Check
PLAT720_ALERT_4_G Number of Unusual/Non-Standard Labels ..........          6 Note
PLAT789_ALERT_4_G Atoms with Negative _atom_site_disorder_group  #         30 Check
PLAT860_ALERT_3_G Number of Least-Squares Restraints .............         15 Note
```

```
   0 ALERT level A = Most likely a serious problem - resolve or explain
   4 ALERT level B = A potentially serious problem, consider carefully
  16 ALERT level C = Check. Ensure it is not caused by an omission or oversight
  44 ALERT level G = General information/check it is not something unexpected

   1 ALERT type 1 CIF construction/syntax error, inconsistent or missing data
  21 ALERT type 2 Indicator that the structure model may be wrong or deficient
   2 ALERT type 3 Indicator that the structure quality may be low
  40 ALERT type 4 Improvement, methodology, query or suggestion
   0 ALERT type 5 Informative message, check
```

Datablock: ESY296

```
Bond precision:    C-C = 0.0060 A            Wavelength=1.54184

Cell:              a=10.2343(1)      b=29.7930(2)        c=20.1214(1)
                   alpha=90          beta=95.889(1)      gamma=90
Temperature:       150 K
```

	Calculated	Reported
Volume	6102.85(8)	6102.85(8)
Space group	P 21/c	P 21/c
Hall group	-P 2ybc	-P 2ybc
Moiety formula	C50 H76 Au N6 Ni P, 2(C6 H6)	C50 H76 Au N6 Ni P, 2(C6 H6)
Sum formula	C62 H88 Au N6 Ni P	C62 H88 Au N6 Ni P
Mr	1204.02	1204.03
Dx,g cm-3	1.310	1.310
Z	4	4
Mu (mm-1)	5.388	5.388
F000	2496.0	2496.0
F000'	2472.41	
h,k,lmax	12,37,25	12,37,25
Nref	12795	12731
Tmin,Tmax	0.436,0.650	0.800,1.000
Tmin'	0.330	

Correction method= # Reported T Limits: Tmin=0.800 Tmax=1.000
AbsCorr = MULTI-SCAN

Data completeness= 0.995 Theta(max)= 76.393

R(reflections)= 0.0333(11234) wR2(reflections)= 0.0891(12731)

S = 1.056 Npar= 648

The following ALERTS were generated. Each ALERT has the format
test-name_ALERT_alert-type_alert-level.
Click on the hyperlinks for more details of the test.

Alert level C

```
PLAT094_ALERT_2_C Ratio of Maximum / Minimum Residual Density ....       3.39 Report
PLAT213_ALERT_2_C Atom C45              has ADP max/min Ratio .....       3.1 prolat
PLAT220_ALERT_2_C NonSolvent   Resd 1  C   Ueq(max)/Ueq(min) Range        5.0 Ratio
PLAT222_ALERT_3_C NonSolvent Resd 1  H   Uiso(max)/Uiso(min) Range        4.2 Ratio
PLAT241_ALERT_2_C High   'MainMol' Ueq as Compared to Neighbors of        C45 Check
PLAT242_ALERT_2_C Low    'MainMol' Ueq as Compared to Neighbors of         N6 Check
PLAT244_ALERT_4_C Low    'Solvent' Ueq as Compared to Neighbors of        C6T Check
PLAT250_ALERT_2_C Large U3/U1 Ratio for Average U(i,j) Tensor ....        3.3 Note
PLAT250_ALERT_2_C Large U3/U1 Ratio for Average U(i,j) Tensor ....        2.1 Note
PLAT250_ALERT_2_C Large U3/U1 Ratio for Average U(i,j) Tensor ....        2.3 Note
PLAT260_ALERT_2_C Large Average Ueq of Residue Including       C1R      0.115 Check
PLAT260_ALERT_2_C Large Average Ueq of Residue Including       C1T      0.127 Check
PLAT260_ALERT_2_C Large Average Ueq of Residue Including       C1S      0.229 Check
PLAT331_ALERT_2_C Small Aver Phenyl C-C Dist C1S      --C6S      .       1.37 Ang.
PLAT360_ALERT_2_C Short  C(sp3)-C(sp3) Bond  C45      - C46      .       1.34 Ang.
PLAT412_ALERT_2_C Short Intra XH3 .. XHn     H32A     ..H35B     .       1.85 Ang.
```

```
                                                       x,y,z  =      1_555 Check
PLAT412_ALERT_2_C Short Intra XH3 .. XHn     H33A     ..H39B     .       1.83 Ang.
                                                       x,y,z  =      1_555 Check
PLAT412_ALERT_2_C Short Intra XH3 .. XHn     H45      ..H46C     .       1.88 Ang.
                                                       x,y,z  =      1_555 Check
```

Alert level G

```
PLAT003_ALERT_2_G Number of Uiso or Uij Restrained non-H Atoms ...         18 Report
PLAT083_ALERT_2_G SHELXL Second Parameter in WGHT  Unusually Large       9.73 Why ?
PLAT142_ALERT_4_G s.u. on b - Axis Small or Missing .............     0.00020 Ang.
PLAT143_ALERT_4_G s.u. on c - Axis Small or Missing .............     0.00010 Ang.
PLAT177_ALERT_4_G The CIF-Embedded .res File Contains DELU Records          3 Report
PLAT178_ALERT_4_G The CIF-Embedded .res File Contains SIMU Records          3 Report
PLAT302_ALERT_4_G Anion/Solvent/Minor-Residue Disorder (Resd  2  )       100% Note
PLAT302_ALERT_4_G Anion/Solvent/Minor-Residue Disorder (Resd  4  )       100% Note
PLAT304_ALERT_4_G Non-Integer Number of Atoms in ..... (Resd  2  )       6.91 Check
PLAT304_ALERT_4_G Non-Integer Number of Atoms in ..... (Resd  4  )       5.09 Check
PLAT343_ALERT_2_G Unusual sp?      Angle Range in Main Residue for         C1 Check
PLAT343_ALERT_2_G Unusual sp3      Angle Range in Main Residue for        C45 Check
PLAT380_ALERT_4_G Incorrectly? Oriented X(sp2)-Methyl Moiety .....        C32 Check
PLAT380_ALERT_4_G Incorrectly? Oriented X(sp2)-Methyl Moiety .....        C33 Check
PLAT380_ALERT_4_G Incorrectly? Oriented X(sp2)-Methyl Moiety .....        C43 Check
PLAT380_ALERT_4_G Incorrectly? Oriented X(sp2)-Methyl Moiety .....        C44 Check
PLAT860_ALERT_3_G Number of Least-Squares Restraints ............         144 Note
```

---

   0 **ALERT level A** = Most likely a serious problem - resolve or explain
   0 **ALERT level B** = A potentially serious problem, consider carefully
  18 **ALERT level C** = Check. Ensure it is not caused by an omission or oversight
  17 **ALERT level G** = General information/check it is not something unexpected

```
   0 ALERT type 1 CIF construction/syntax error, inconsistent or missing data
  20 ALERT type 2 Indicator that the structure model may be wrong or deficient
   2 ALERT type 3 Indicator that the structure quality may be low
  13 ALERT type 4 Improvement, methodology, query or suggestion
   0 ALERT type 5 Informative message, check
```

Datablock: ESY238

Bond precision: C-C = 0.0082 A   Wavelength=1.54184

Cell:	a=17.2262(1)	b=20.7164(2)	c=27.5711(2)
	alpha=90	beta=90	gamma=90
Temperature:	150 K		

	Calculated	Reported
Volume	9839.16(13)	9839.16(13)
Space group	P b c a	P b c a
Hall group	-P 2ac 2ab	-P 2ac 2ab
Moiety formula	C41 H60 Au N2 P2 Rh, C6 H14	C41 H60 Au N2 P2 Rh, C6 H14
Sum formula	C47 H74 Au N2 P2 Rh	C47 H74 Au N2 P2 Rh
Mr	1028.90	1028.89
Dx,g cm-3	1.389	1.389
Z	8	8
Mu (mm-1)	9.072	9.072
F000	4192.0	4192.0
F000'	4169.77	
h,k,lmax	21,26,34	21,26,34
Nref	10356	10313
Tmin,Tmax	0.170,0.195	0.385,1.000
Tmin'	0.076	

Correction method= # Reported T Limits: Tmin=0.385 Tmax=1.000
AbsCorr = MULTI-SCAN

Data completeness= 0.996 Theta(max)= 76.590

R(reflections)= 0.0370(9548) wR2(reflections)= 0.0938(10313)

S = 1.030 Npar= 494

The following ALERTS were generated. Each ALERT has the format
test-name_ALERT_alert-type_alert-level.
Click on the hyperlinks for more details of the test.

Alert level C

```
PLAT220_ALERT_2_C NonSolvent   Resd 1  C   Ueq(max)/Ueq(min) Range        3.5 Ratio
PLAT242_ALERT_2_C Low    'MainMol' Ueq as Compared to Neighbors of        C23 Check
PLAT244_ALERT_4_C Low    'Solvent' Ueq as Compared to Neighbors of        C2S Check
PLAT244_ALERT_4_C Low    'Solvent' Ueq as Compared to Neighbors of        C5S Check
PLAT250_ALERT_2_C Large U3/U1 Ratio for Average U(i,j) Tensor ....        2.9 Note
PLAT260_ALERT_2_C Large Average Ueq of Residue Including      C1S      0.121 Check
PLAT342_ALERT_3_C Low Bond Precision on  C-C Bonds ...............   0.00817 Ang.
PLAT360_ALERT_2_C Short  C(sp3)-C(sp3) Bond  C1S      - C2S      .       1.42 Ang.
```

Alert level G

```
PLAT083_ALERT_2_G SHELXL Second Parameter in WGHT  Unusually Large      42.11 Why ?
PLAT142_ALERT_4_G s.u. on b - Axis Small or Missing .............   0.00020 Ang.
PLAT143_ALERT_4_G s.u. on c - Axis Small or Missing .............   0.00020 Ang.
PLAT232_ALERT_2_G Hirshfeld Test Diff (M-X)  Rh1      --P1       .        5.8 s.u.
PLAT343_ALERT_2_G Unusual sp?     Angle Range in Main Residue for         C1 Check
PLAT720_ALERT_4_G Number of Unusual/Non-Standard Labels ..........         14 Note
```

0 **ALERT level A** = Most likely a serious problem - resolve or explain
0 **ALERT level B** = A potentially serious problem, consider carefully
8 **ALERT level C** = Check. Ensure it is not caused by an omission or oversight
6 **ALERT level G** = General information/check it is not something unexpected

0 ALERT type 1 CIF construction/syntax error, inconsistent or missing data
8 ALERT type 2 Indicator that the structure model may be wrong or deficient
1 ALERT type 3 Indicator that the structure quality may be low
5 ALERT type 4 Improvement, methodology, query or suggestion
0 ALERT type 5 Informative message, check

Datablock: ESY220528

Bond precision: C-C = 0.0094 A   Wavelength=1.54184

Cell:	a=20.4976(2)	b=10.5365(1)	c=25.2448(2)
	alpha=90	beta=97.291(1)	gamma=90
Temperature:	150 K		

	Calculated	Reported
Volume	5408.11(9)	5408.11(9)
Space group	P 21/c	P 21/c
Hall group	-P 2ybc	-P 2ybc
Moiety formula	C46 H60 Au N2 O5 P2 Rh W, 0.5(C6 H14)	C46 H60 Au N2 O5 P2 Rh W, 0.5(C6 H14)
Sum formula	C49 H67 Au N2 O5 P2 Rh W	C49 H67 Au N2 O5 P2 Rh W
Mr	1309.71	1309.71
Dx,g cm-3	1.609	1.609
Z	4	4
Mu (mm-1)	12.172	12.172
F000	2572.0	2572.0
F000'	2540.36	
h,k,lmax	25,13,31	25,13,31
Nref	11307	11265
Tmin,Tmax	0.102,0.296	0.294,1.000
Tmin'	0.028	

Correction method= # Reported T Limits: Tmin=0.294 Tmax=1.000
AbsCorr = MULTI-SCAN

Data completeness= 0.996   Theta(max)= 76.158

R(reflections)= 0.0322(9980)   wR2(reflections)= 0.0838(11265)

S = 1.092   Npar= 566

The following ALERTS were generated. Each ALERT has the format
test-name_ALERT_alert-type_alert-level.
Click on the hyperlinks for more details of the test.

Alert level C

```
PLAT220_ALERT_2_C NonSolvent   Resd 1  C   Ueq(max)/Ueq(min) Range        4.2 Ratio
PLAT222_ALERT_3_C NonSolvent Resd 1  H   Uiso(max)/Uiso(min) Range        4.2 Ratio
PLAT242_ALERT_2_C Low    'MainMol' Ueq as Compared to Neighbors of         P2 Check
PLAT242_ALERT_2_C Low    'MainMol' Ueq as Compared to Neighbors of        C11 Check
PLAT242_ALERT_2_C Low    'MainMol' Ueq as Compared to Neighbors of        C14 Check
PLAT242_ALERT_2_C Low    'MainMol' Ueq as Compared to Neighbors of        C23 Check
PLAT243_ALERT_4_C High   'Solvent' Ueq as Compared to Neighbors of        C2S Check
PLAT260_ALERT_2_C Large Average Ueq of Residue Including       C1S      0.278 Check
PLAT342_ALERT_3_C Low Bond Precision on  C-C Bonds ...............    0.00937 Ang.
```

Alert level G

```
PLAT002_ALERT_2_G Number of Distance or Angle Restraints on AtSite          3 Note
PLAT003_ALERT_2_G Number of Uiso or Uij Restrained non-H Atoms ...          3 Report
PLAT083_ALERT_2_G SHELXL Second Parameter in WGHT  Unusually Large       9.93 Why ?
PLAT142_ALERT_4_G s.u. on b - Axis Small or Missing .............    0.00010 Ang.
PLAT143_ALERT_4_G s.u. on c - Axis Small or Missing .............    0.00020 Ang.
PLAT172_ALERT_4_G The CIF-Embedded .res File Contains DFIX Records          3 Report
PLAT186_ALERT_4_G The CIF-Embedded .res File Contains ISOR Records          1 Report
PLAT343_ALERT_2_G Unusual sp?      Angle Range in Main Residue for         C1 Check
PLAT380_ALERT_4_G Incorrectly? Oriented X(sp2)-Methyl Moiety .....        C36 Check
PLAT720_ALERT_4_G Number of Unusual/Non-Standard Labels ..........          7 Note
PLAT860_ALERT_3_G Number of Least-Squares Restraints ............          21 Note
```

0 **ALERT level A** = Most likely a serious problem - resolve or explain
0 **ALERT level B** = A potentially serious problem, consider carefully
9 **ALERT level C** = Check. Ensure it is not caused by an omission or oversight
11 **ALERT level G** = General information/check it is not something unexpected

0 ALERT type 1 CIF construction/syntax error, inconsistent or missing data
10 ALERT type 2 Indicator that the structure model may be wrong or deficient
3 ALERT type 3 Indicator that the structure quality may be low
7 ALERT type 4 Improvement, methodology, query or suggestion
0 ALERT type 5 Informative message, check

It is advisable to attempt to resolve as many as possible of the alerts in all categories. Often the minor alerts point to easily fixed oversights, errors and omissions in your CIF or refinement strategy, so attention to these fine details can be worthwhile. In order to resolve some of the more serious problems it may be necessary to carry out additional measurements or structure refinements. However, the purpose of your study may justify the reported deviations and the more serious of these should normally be commented upon in the discussion or experimental section of a paper or in the "special_details" fields of the CIF. checkCIF was carefully designed to identify outliers and unusual parameters, but every test has its limitations and alerts that are not important in a particular case may appear. Conversely, the absence of alerts does not guarantee there are no aspects of the results needing attention. It is up to the individual to critically assess their own results and, if necessary, seek expert advice.

Publication of your CIF in IUCr journals

A basic structural check has been run on your CIF. These basic checks will be run on all CIFs submitted for publication in IUCr journals (*Acta Crystallographica*, *Journal of Applied Crystallography*, *Journal of Synchrotron Radiation*); however, if you intend to submit to *Acta Crystallographica Section C* or *E* or *IUCrData*, you should make sure that full publication checks are run on the final version of your CIF prior to submission.

Publication of your CIF in other journals

Please refer to the *Notes for Authors* of the relevant journal for any special instructions relating to CIF submission.

PLATON version of 18/05/2022; check.def file version of 17/05/2022

Datablock ESY041_2 - ellipsoid plot

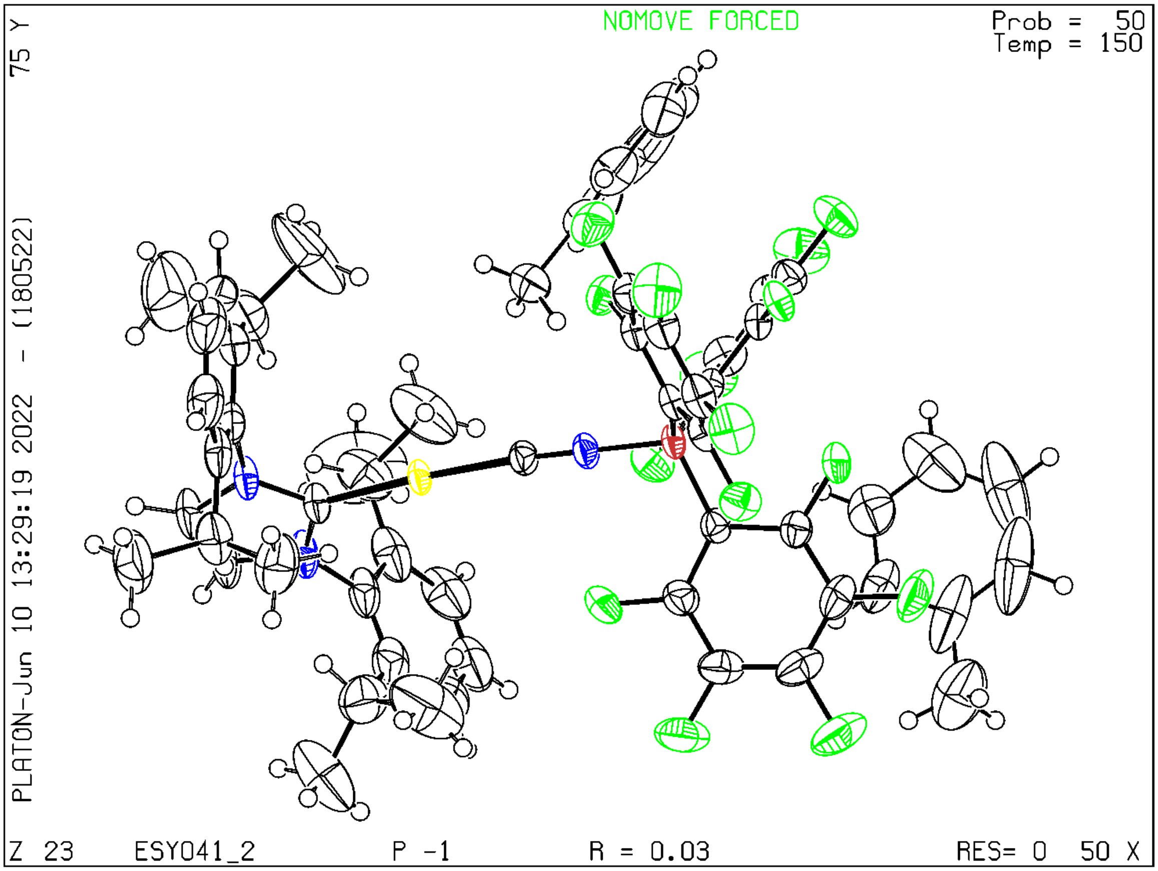

Datablock ESY296 - ellipsoid plot

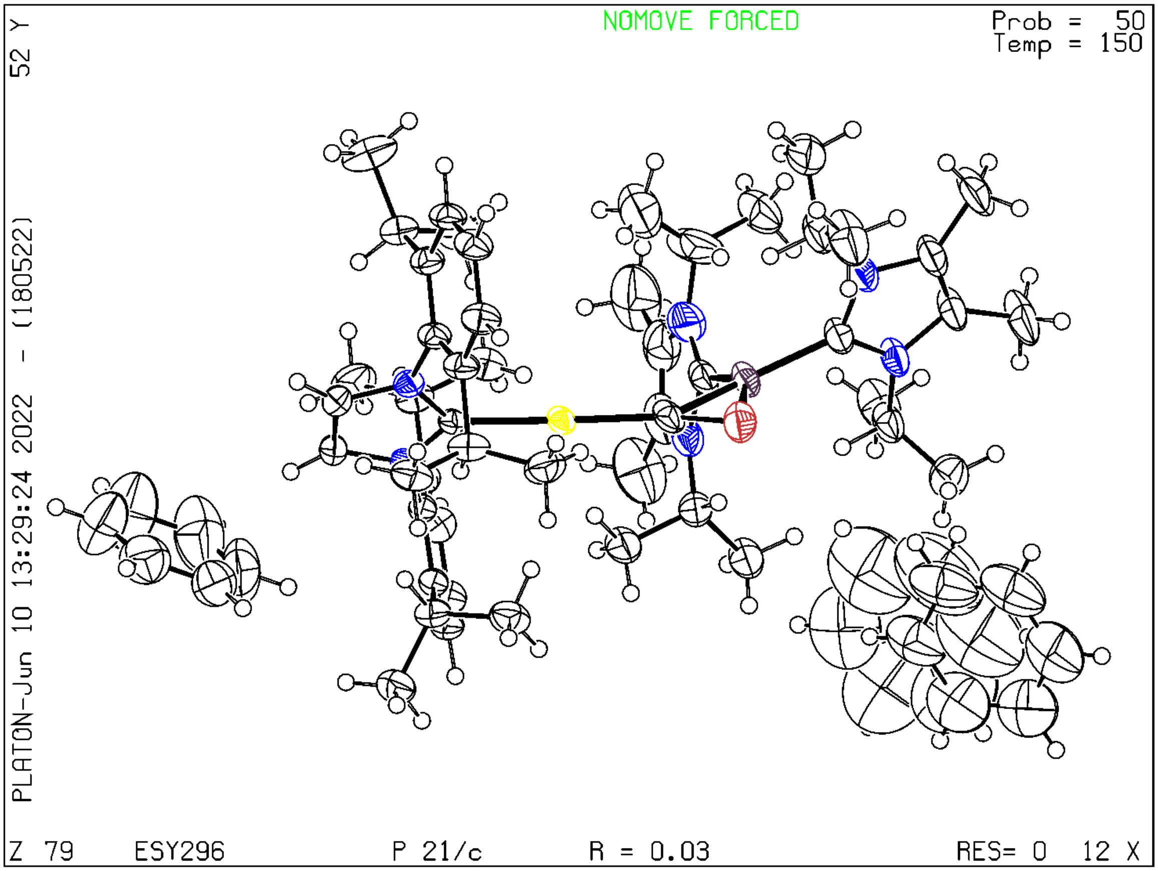

Datablock ESY238 - ellipsoid plot

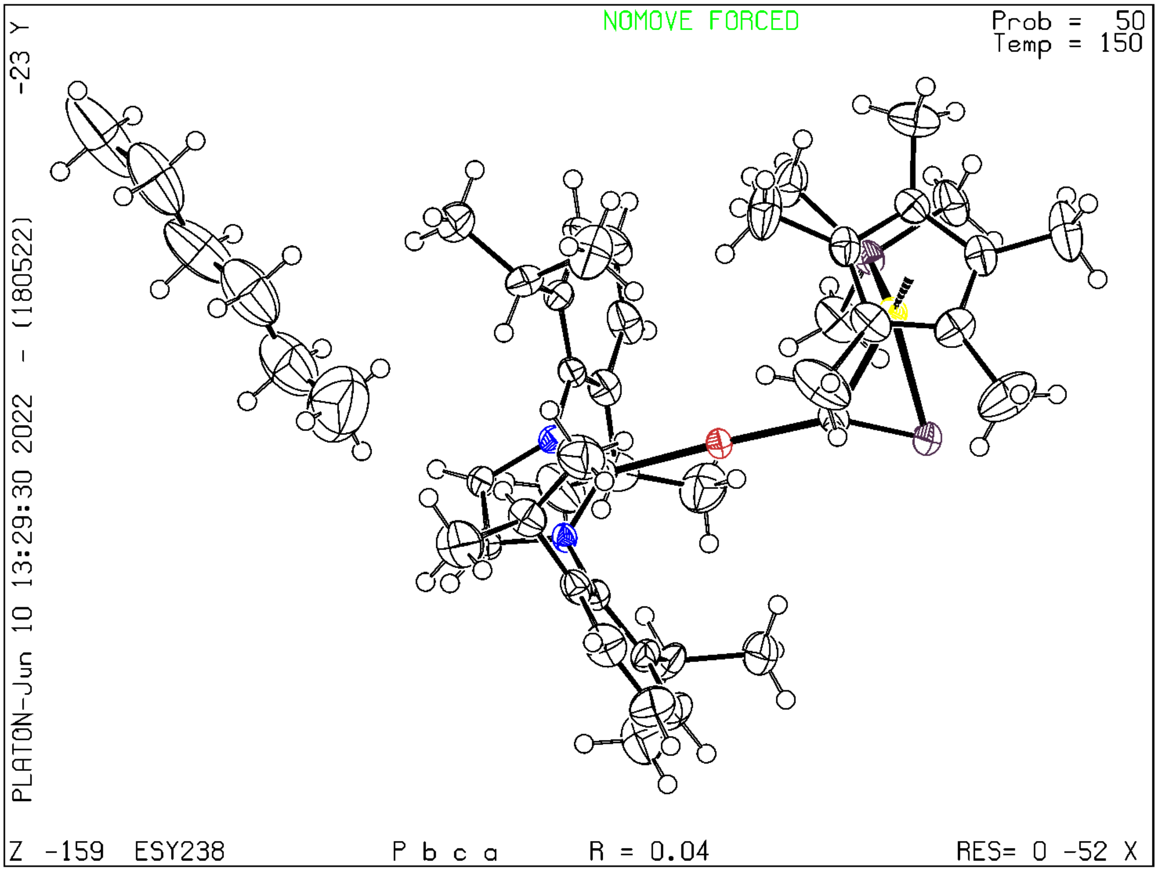

Datablock ESY220528 - ellipsoid plot

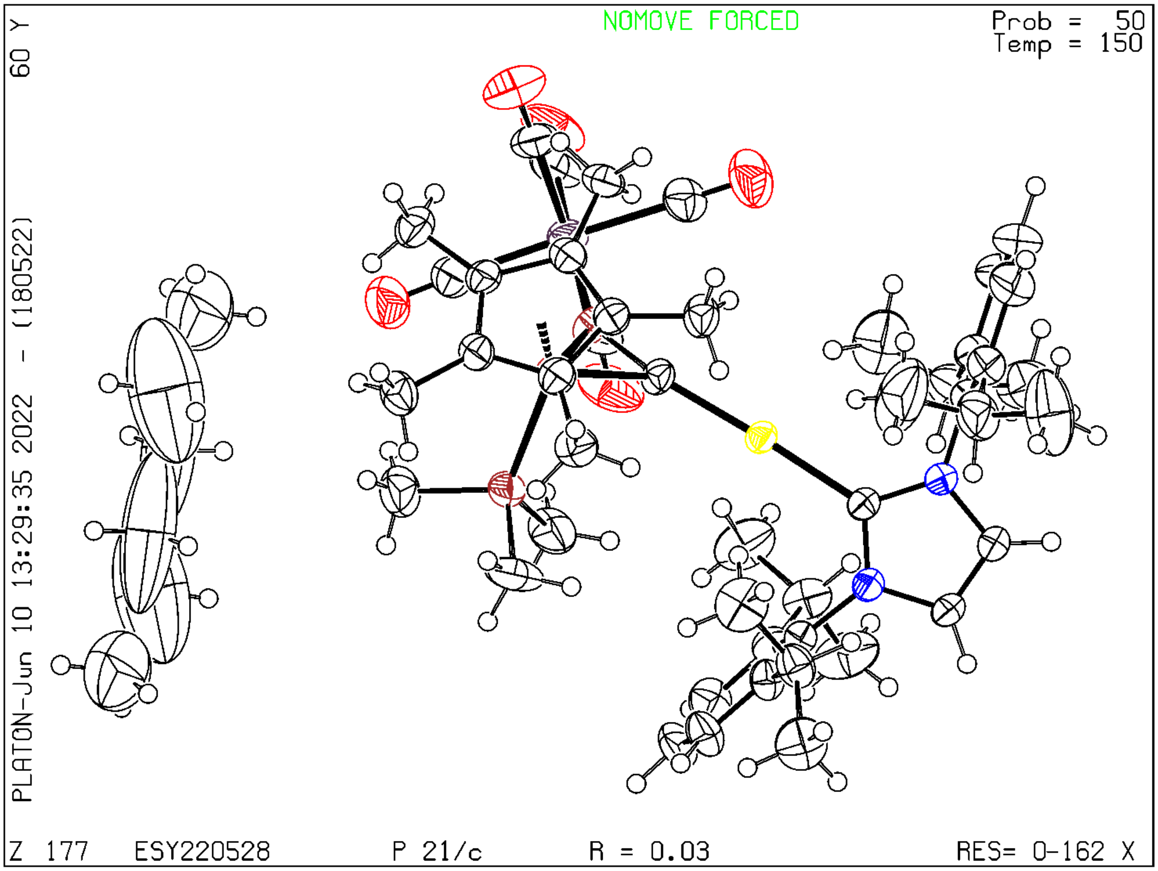

Supplement: Supplementary file 1 — Supporting Information [file ANIE-61-0-s003.pdf]
