# Supplementary material for: Revealing the Role of the Cyaphide Ion as a Bridging Ligand in Heterometallic Complexes
Source: Angew Chem Int Ed Engl. 2022 Jul 6;61(33):e202206783. doi: 10.1002/anie.202206783 (PMC9546431; doi:10.1002/anie.202206783)
Supplement: Supplementary file 3 — Supporting Information [file ANIE-61-0-s002.pdf]

Angewandte Chemie
Eine Zeitschrift der Gesellschaft Deutscher Chemiker

Supporting Information

Revealing the Role of the Cyaphide Ion as a Bridging Ligand in Heterometallic Complexes

*E. S. Yang, J. M. Goicoechea**

Supporting Information

Contents

1. Experimental section
    1.1. General synthetic methods
    1.2. Synthesis of reported compounds
    1.3. Additional characterization
2. Single crystal X-ray diffraction data
3. Computational details
    3.1 General computational methods
    3.2 Energy decomposition analysis
    3.3 Data analysis
    3.4 Other computational results
    3.5 XYZ coordinates
4. References

1. Experimental section

1.1 General experimental methods

Synthetic methods:

All reactions and product manipulations were carried out using standard Schlenk-line techniques under an inert atmosphere of argon, or in a dinitrogen filled glovebox (MBraun UNIlab glovebox maintained at < 0.1 ppm H_2O and < 0.1 ppm O_2). Au(IDipp)(C≡P),[1] Rh(Cp*)$(PMe_3)_2$,[2] $W(CO)_5$(THF),[3] and Au(IDipp)(CN)[4] were synthesized according to previously reported procedures. $(^{Me}I^{i}Pr_2)_2$Ni(COD) and $[(^{Me}I_{i}Pr_2)_2Ni]_2$(μ-COD) were synthesized as a 7:3 mixture according to a previously reported procedure,[5] and its stoichiometry determined by ^{1}H NMR spectroscopy. Tungsten hexacarbonyl (Sigma Aldrich), was used as received. Hexane (Sigma Aldrich HPLC grade) and toluene (Sigma Aldrich HPLC grade) were purified using an MBraun SPS-800 solvent system. THF (Sigma Aldrich HPLC grade) was distilled over sodium/benzophenone. C_6D_6 (Aldrich, 99.5%) was degassed and dried over CaH_2. CD_2Cl_2 (Aldrich, 99.5%) was degassed and dried over activated 3 Å molecular sieves. All dry solvents were stored under argon in gas-tight ampoules over activated 3 Å molecular sieves.

Characterization techniques:

NMR spectra were acquired on a Bruker AVIII 400 MHz NMR spectrometer (^{1}H 400 MHz, ^{11}B 128 MHz, ^{19}F 377 MHz, ^{31}P 162 MHz) or a Bruker Avance NEO 600 MHz NMR spectrometer with a broadband helium cryoprobe (^{1}H 600 MHz, ^{13}C 151 MHz). ^{1}H and ^{13}C NMR spectra were referenced to the most downfield solvent resonance (^{1}H NMR C_6D_6: δ = 7.16 ppm, CD_2Cl_2: δ = 5.32 ppm; ^{13}C NMR C_6D_6: δ = 128.06 ppm). ^{11}B NMR spectra were externally referenced to $BF_3{\cdot}Et_2O$ in C_6D_6. ^{19}F NMR spectra were externally referenced to $CFCl_3$. ^{31}P NMR spectra were externally referenced to an 85% solution of H_3PO_4 in H_2O. Infrared spectra were acquired on a Thermo Scientific iS5 FTIR spectrometer using an iD3 ATR stage. Raman spectra were acquired on a Thermo Scientific DXR3 SmartRaman spectrometer using a 785 nm laser. Elemental analyses were carried out by Elemental Microanalyses Ltd. (Devon, U.K.). Samples (approx. 5 mg) were submitted in flame sealed glass tubes.

1.2 Synthesis of reported compounds

1.2.1 Synthesis of Au(IDipp)(μ$_2$-CN)B(C_6F_5)$_3$ (1)

Au(IDipp)(CN) (18 mg, 0.03 mmol) and B(C_6F_5)$_3$ (15 mg, 0.03 mmol) were dissolved in dichloromethane (1 mL), and the reaction mixture stirred for 15 min. The solvent was removed under vacuum, and the resulting residue was washed with hexane then crystallized from toluene by slow evaporation, affording Au(IDipp)(μ$_2$-CN)B(C_6F_5)$_3$ (**1**) as colorless crystals (27 mg, 0.02 mmol, 82% yield). Anal. Calcd. (%) for $C_{46}H_{36}AuBF_{15}N_3$: C, 49.17; H, 3.23; N, 3.74. Found: 50.14; H, 3.17; N, 3.26.

^{1}H NMR (600 MHz, C_6D_6): δ(ppm) 7.10 (t, $^3J_{H\text{–}H}$ = 7.8 Hz, 2H; Dipp *para*-C**H**), 6.92 (d, $^3J_{H\text{–}H}$ = 7.8 Hz, 4H; Dipp *meta*-C**H**), 6.11 (s, 2H; IDipp C**H**), 2.25 (sept, $^3J_{H\text{–}H}$ = 6.9 Hz, 4H; Dipp C**H**(CH_3)$_2$), 1.21 (d, $^3J_{H\text{–}H}$ = 6.9 Hz, 12H; Dipp CH(C**H**$_3$)$_2$), 0.94 (d, $^3J_{H\text{–}H}$ = 6.9 Hz, 12H; Dipp CH(C**H**$_3$)$_2$).

^{13}C{^{1}H} NMR (151 MHz, C_6D_6): δ(ppm) 183.06 (IDipp [HCN(Dipp)]$_2$**C**Au), 152.68 (μ$_2$-**C**≡N), 148.64 (bd, $^1J_{C\text{–}F}$ = 242.2 Hz; C_6F_5 *ortho*-**C**), 145.40 (Dipp *ortho*-**C**), 140.26 (bd, $^1J_{C\text{–}F}$ = 249.7 Hz; C_6F_5 *para*-**C**), 137.50 (bd, $^1J_{C\text{–}F}$ = 248.9 Hz; C_6F_5 *meta*-**C**), 133.01 (Dipp *ipso*-**C**), 131.73 (Dipp *para*-**C**), 124.69 (Dipp *meta*-**C**), 123.74 (IDipp [H**C**N(Dipp)]$_2$CAu), 117.93 (b, C_6F_5 *ipso*-**C**), 29.01 (Dipp **C**H(CH_3)$_2$), 24.56 (Dipp CH(**C**H$_3$)$_2$), 23.77 (Dipp CH(**C**H$_3$)$_2$).

^{11}B{^{1}H} NMR (128 MHz, C_6D_6): δ(ppm) −12.70 (bs, **B**(C_6F_5)$_3$).

^{19}F{^{1}H} NMR (377 MHz, C_6D_6): δ(ppm) −133.94 (dd, $^3J_{F\text{–}F}$ = 23.7 Hz, $^4J_{F\text{–}F}$ = 8.7 Hz, C_6F_5 *ortho*-**F**), −158.37 (t, $^3J_{F\text{–}F}$ = 20.8 Hz, C_6F_5 para-**F**), −164.84 (m, C_6F_5 *meta*-**F**).

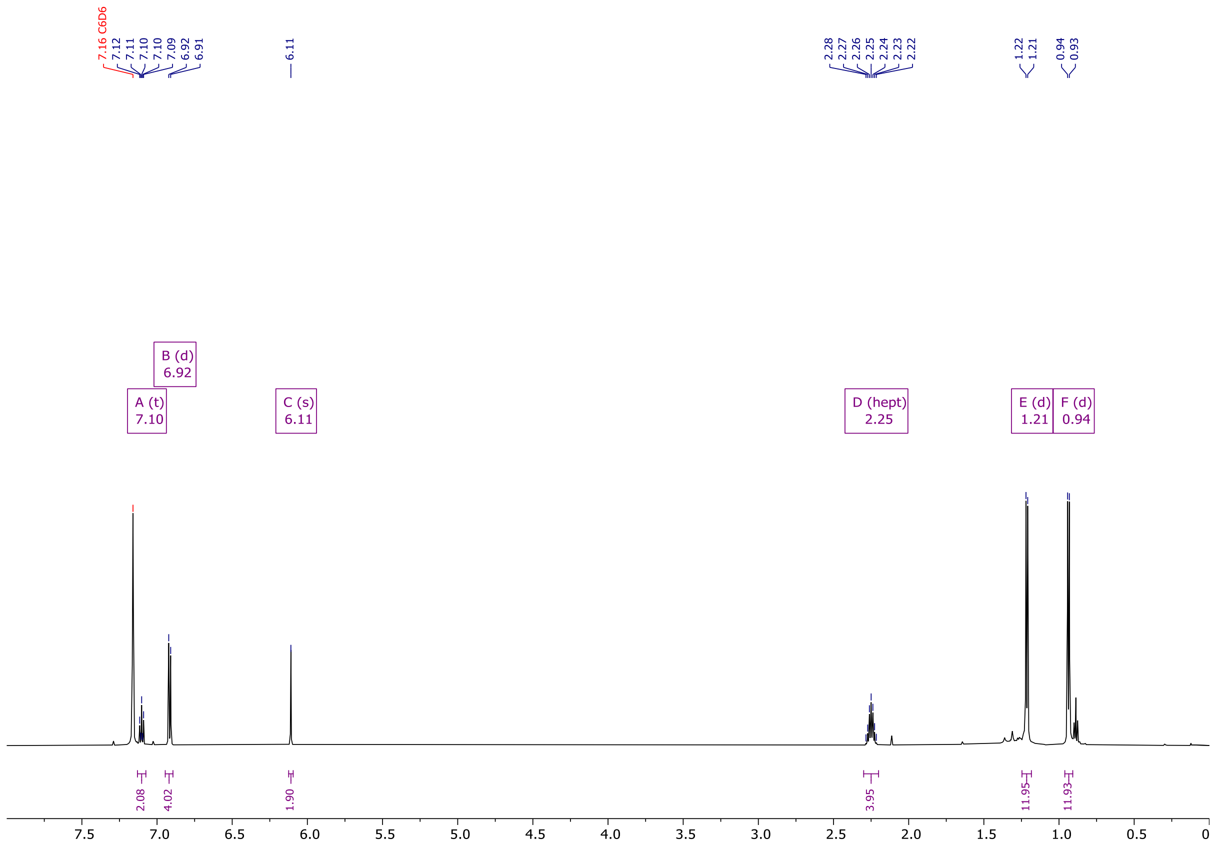

Figure S1. ${}^{1}H$ NMR spectrum of **1** in C_6D_6.

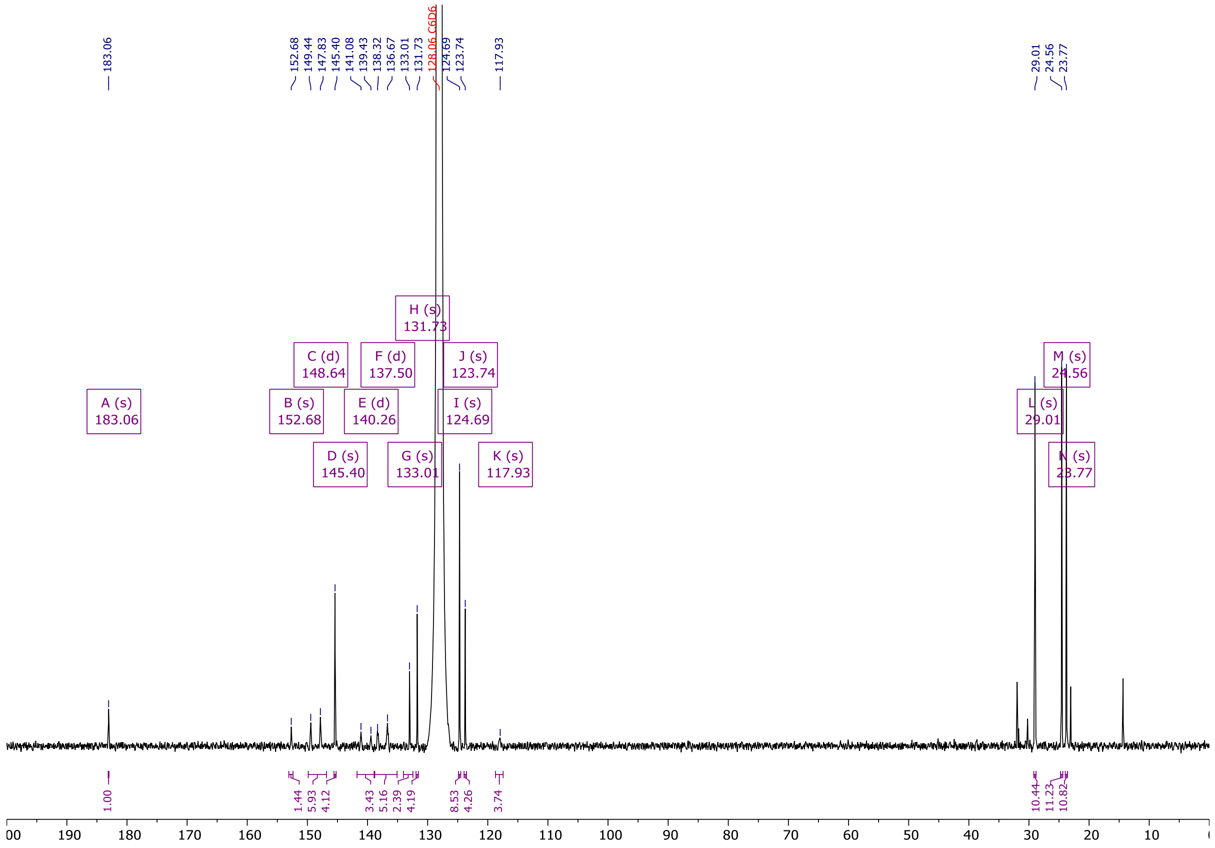

Figure S2. ${}^{13}C\{{}^{1}H\}$ NMR spectrum of **1** in C_6D_6.

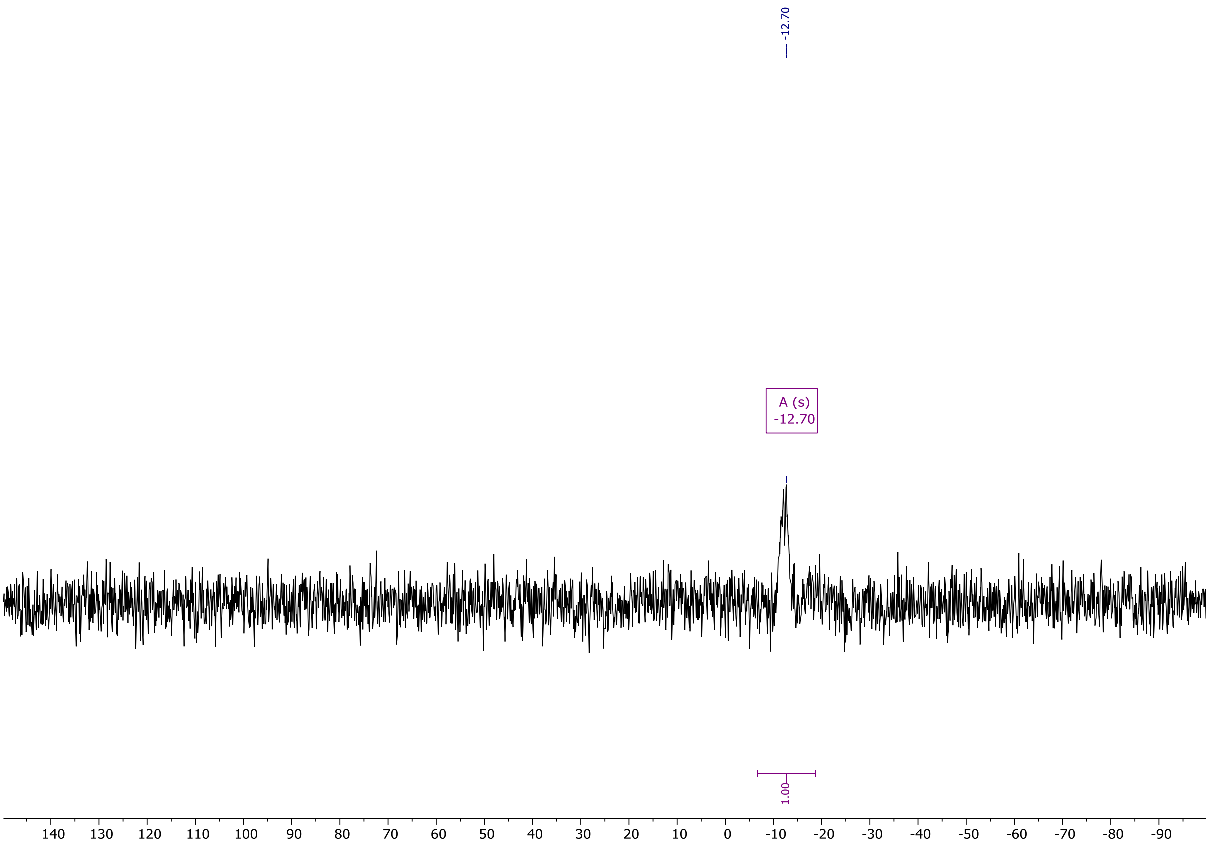

Figure S3. $^{11}B\{^1H\}$ NMR spectrum of **1** in C_6D_6.

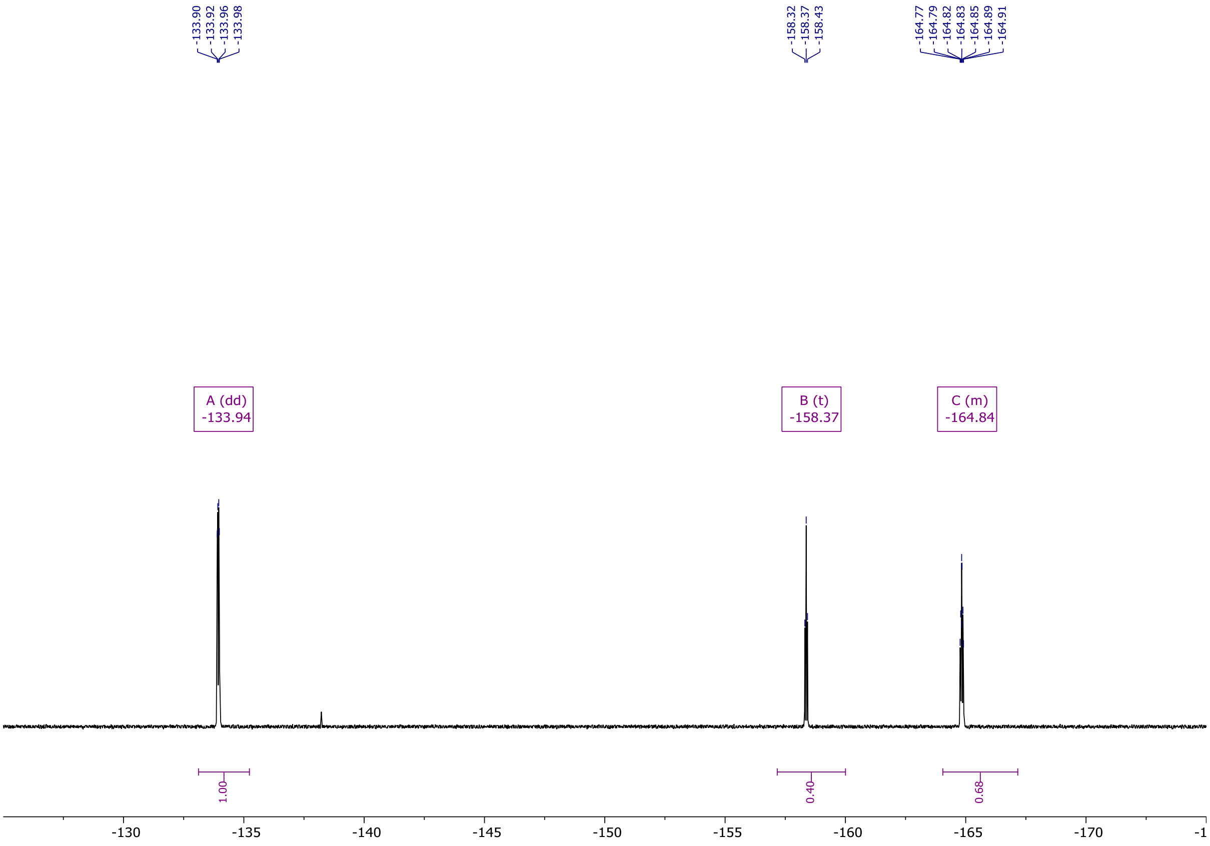

Figure S4. $^{19}F\{^1H\}$ NMR spectrum of **1** in C_6D_6.

1.2.2 Synthesis of Au(IDipp)(μ_2-C≡P)Ni(^{Me}I^{i}Pr$_2$)$_2$ (2)

Au(IDipp)(C≡P) (**A**) (50 mg, 0.08 mmol) and a 7:3 mixture of Ni(^{Me}I^{i}Pr$_2$)$_2$(COD) and {Ni(^{Me}I^{i}Pr$_2$)$_2$Ni}$_2$(μ_2-COD) (41 mg, 0.08 mmol [Ni(^{Me}I^{i}Pr$_2$)$_2$]) were dissolved in benzene (5 mL), immediately resulting in a brown-black solution. The reaction mixture was stirred for 1 h then filtered to remove any particulates. All volatiles were then removed under vacuum by lyophilization, affording Au(IDipp)(μ_2-C≡P)Ni(^{Me}I^{i}Pr$_2$)$_2$ (**2**) as a brown powder (70 mg, 0.07 mmol, 84% yield). Crystals suitable for X-ray diffraction were obtained by slow evaporation of a concentrated benzene/hexane solution. Anal. Calcd. (%) for $C_{50}H_{76}AuN_6NiP$: C, 57.31; H, 7.31; N, 8.02. Found: 57.84; H, 6.79; N, 6.91.

^{1}H NMR (400 MHz, C_6D_6): δ(ppm) 7.13 (t, $^3J_{H–H}$ = 7.4 Hz, 2H; Dipp *para*-C**H**), 7.05 (d, $^3J_{H–H}$ = 7.4 Hz, 4H; Dipp *meta*-C**H**), 6.33 (s, 2H; IDipp C**H**), 5.87 (sept, $^3J_{H–H}$ = 7.0 Hz, 2H; ^{Me}I^{i}Pr$_2$ C**H**(CH$_3$)$_2$), 5.82 (sept, $^3J_{H–H}$ = 7.0 Hz, 2H; ^{Me}I^{i}Pr$_2$ C**H**(CH$_3$)$_2$), 2.79 (sept, $^3J_{H–H}$ = 6.8 Hz, 4H; Dipp C**H**(CH$_3$)$_2$), 1.99 (s, 6H; ^{Me}I^{i}Pr$_2$ C(C**H$_3$**)), 1.85 (s, 6H; ^{Me}I^{i}Pr$_2$ C(C**H$_3$**)), 1.51 (d, $^3J_{H–H}$ = 6.8 Hz, 12H; Dipp CH(C**H$_3$**)$_2$), 1.22 (d, $^3J_{H–H}$ = 7.0 Hz, 16H; Dipp CH(C**H$_3$**)$_2$), 1.14 (d, $^3J_{H–H}$ = 7.0 Hz, 12H; ^{Me}I^{i}Pr$_2$ CH(C**H$_3$**)$_2$), 1.12 (d, $^3J_{H–H}$ = 7.0 Hz, 12H; ^{Me}I^{i}Pr$_2$ CH(C**H$_3$**)$_2$).

^{13}C{^{1}H} NMR (151 MHz, C_6D_6): δ(ppm) 280.32 (d, $^1J_{C–P}$ = 105.1 Hz; μ_2-**C**≡P), 207.84 (d, $^2J_{C–P}$ = 23.7 Hz; ^{Me}I^{i}Pr$_2$ [H$_3$CCN(^{i}Pr)]$_2$**C**Ni), 198.46 (IDipp [HCN(Dipp)]$_2$**C**Au), 145.50 (Dipp *ortho*-**C**), 135.51 (Dipp *ipso*-**C**), 129.70 (Dipp *para*-**C**), 123.64 (Dipp *meta*-**C**), 123.33 (^{Me}I^{i}Pr$_2$ [H$_3$C**C**N(^{i}Pr)]$_2$CNi), 121.99 (^{Me}I^{i}Pr$_2$ [H$_3$C**C**N(^{i}Pr)]$_2$CNi), 121.88 (IDipp [H**C**N(Dipp)]$_2$CAu), 51.31 (^{Me}I^{i}Pr$_2$ **C**H(CH$_3$)$_2$), 50.93 (^{Me}I^{i}Pr$_2$ **C**H(CH$_3$)$_2$), 28.60 (Dipp **C**H(CH$_3$)$_2$), 24.28 (Dipp CH(**C**H$_3$)$_2$), 23.81 (^{Me}I^{i}Pr$_2$ CH(**C**H$_3$)$_2$), 22.26 (Dipp CH(**C**H$_3$)$_2$), 21.57 (^{Me}I^{i}Pr$_2$ CH(**C**H$_3$)$_2$), 10.26 (^{Me}I^{i}Pr$_2$ [H$_3$**C**CN(^{i}Pr)]$_2$CNi), 10.22 (^{Me}I^{i}Pr$_2$ [H$_3$**C**CN(^{i}Pr)]$_2$CNi).

^{31}P{^{1}H} NMR (162 MHz, C_6D_6): δ(ppm) 246.02 (s, μ_2-C≡**P**).

Raman: 1125 cm^{-1} (mw, *v*(C≡P)).

UV-vis: λ_{max} (nm) 317.

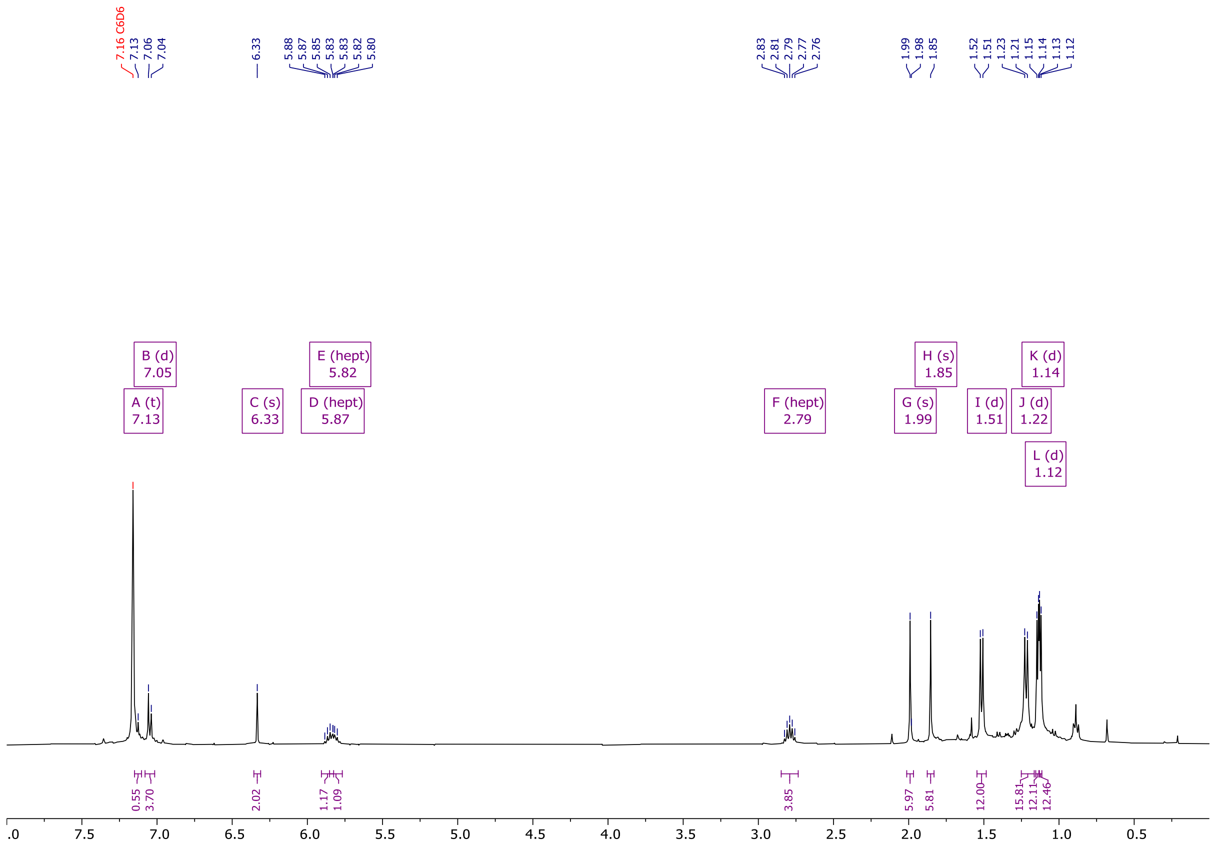

Figure S5. ^{1}H NMR spectrum of **2** in C_6D_6.

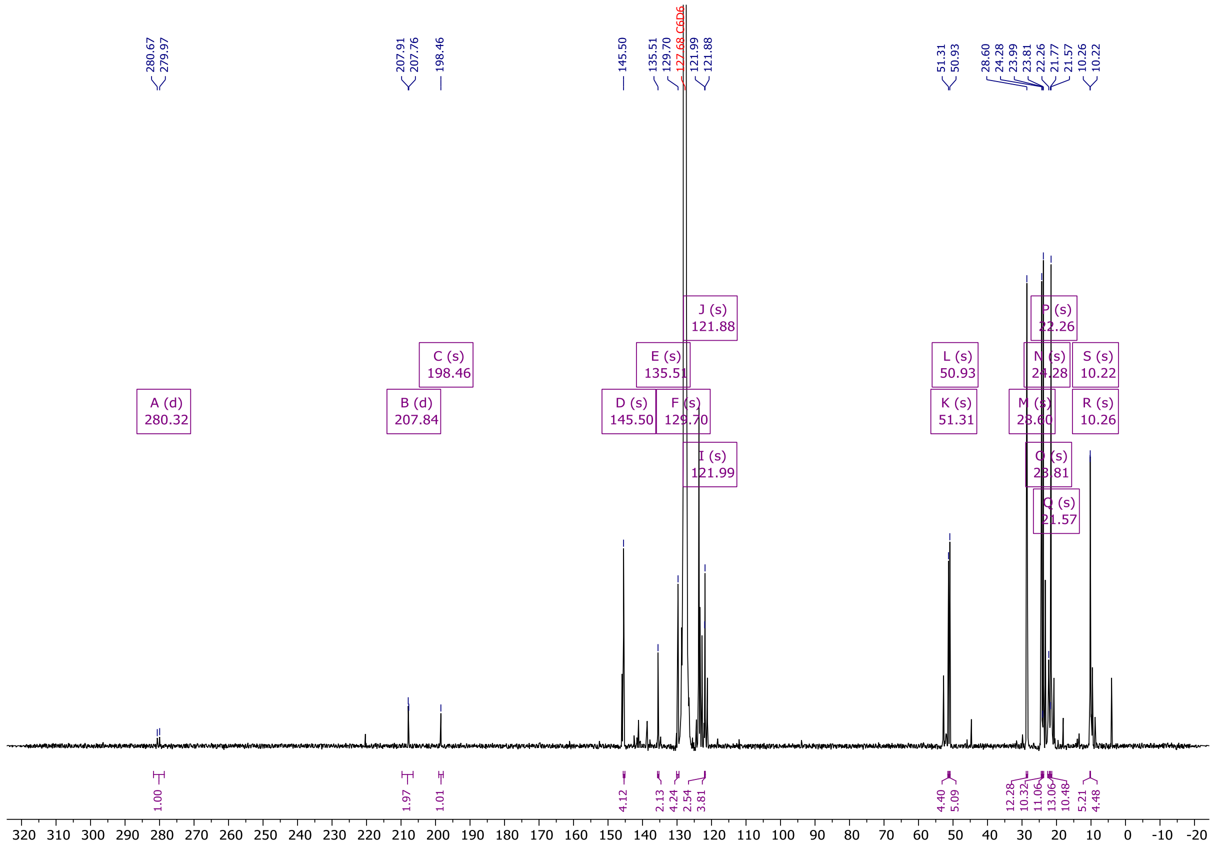

Figure S6. $^{13}C\{^{1}H\}$ NMR spectrum of **2** in C_6D_6.

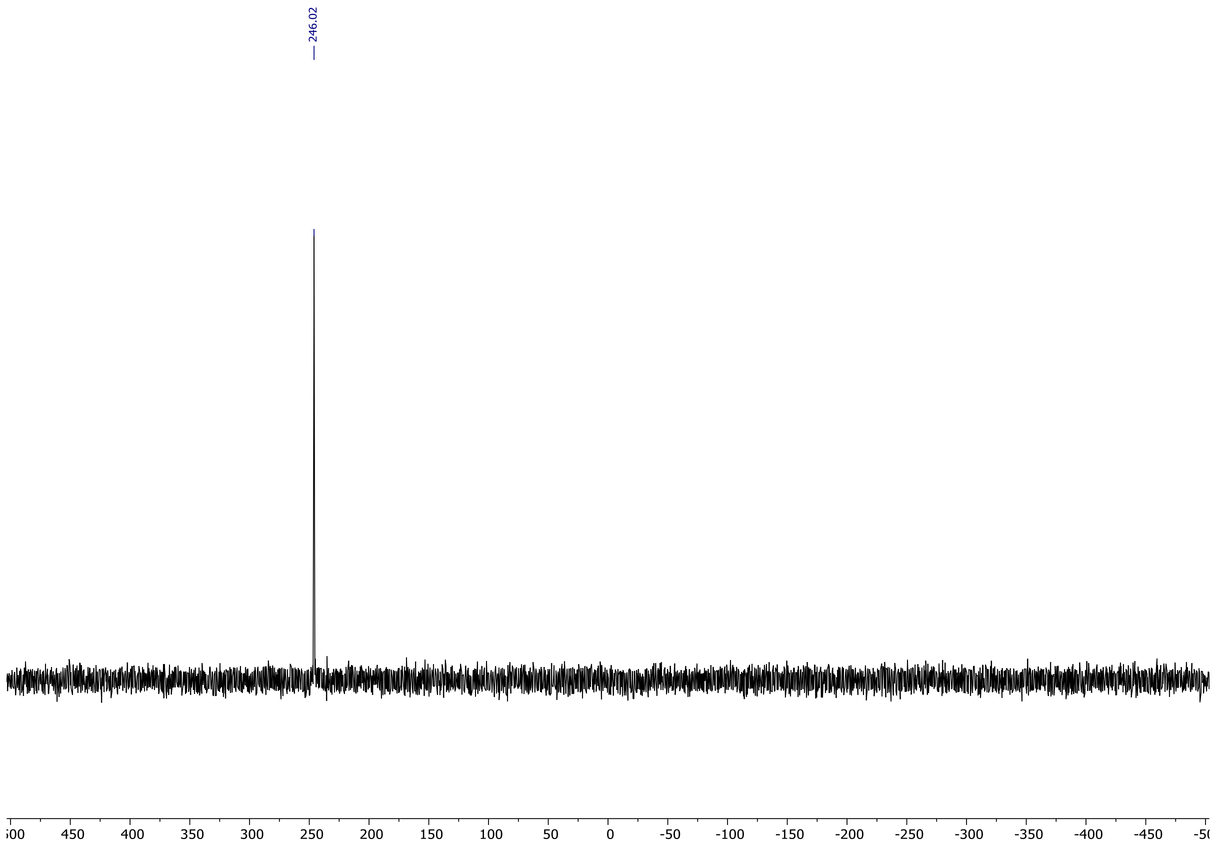

Figure S7. $^{31}P\{^1H\}$ NMR spectrum of **2** in C_6D_6.

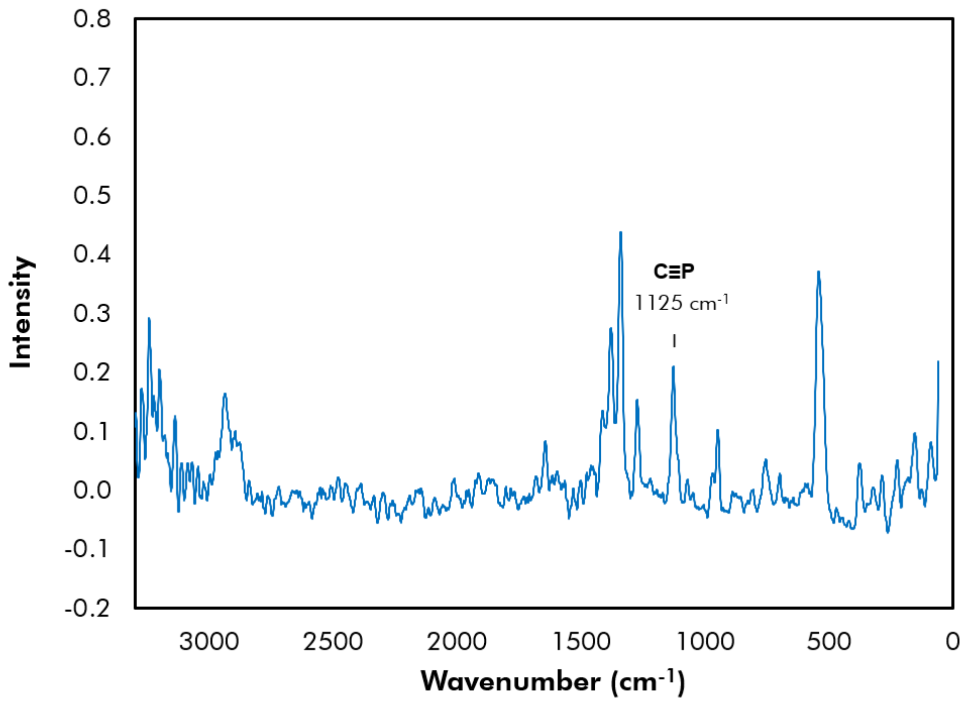

Figure S8. Dispersive Raman spectrum of **2**.

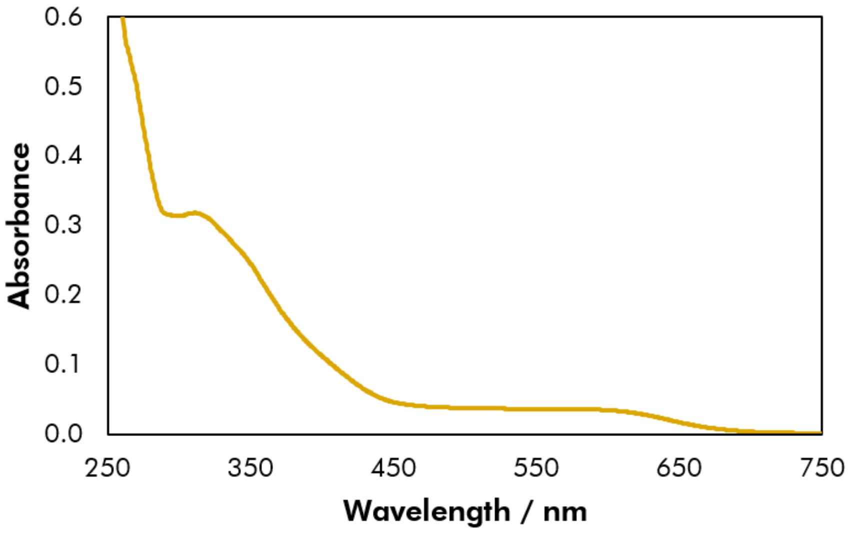

Figure S9. UV-visible absorption spectrum of **2**.

1.2.3 Synthesis of Au(IDipp)(μ$_2$-C≡P)Rh(Cp*)(PMe$_3$) (3)

A (100 mg, 0.16 mmol) and Rh(Cp*)(PMe$_3$)$_2$ (62 mg, 0.16 mmol) were dissolved in toluene (10 mL). The reaction mixture was heated at 60 °C overnight, resulting in an orange-red solution. All volatiles were removed under vacuum and the residue lyophilized from benzene (10 mL) to give Au(IDipp)(μ$_2$-C≡P)Rh(Cp*)(PMe$_3$) (**3**) as an orange-red powder suitable for further synthesis (135 mg, 0.14 mmol, 90% yield). Compositionally pure crystals suitable for X-ray diffraction can be obtained by slow evaporation of a concentrated benzene/hexane solution. Anal. Calcd. (%) for $C_{41}H_{60}AuN_2P_2Rh{\cdot}C_6H_6$: C, 55.30; H, 6.52; N, 2.74. Found: 54.65; H, 5.90; N, 2.71.

^{1}H NMR (600 MHz, C$_6$D$_6$): δ(ppm) 7.20 (dd, $^3J_{H–H}$ = 7.8 Hz, 2H; Dipp *para*-C**H**), 7.08 (dd, $^3J_{H–H}$ = 7.8 Hz, 2H; Dipp *meta*-C**H**), 7.08 (dd, $^3J_{H–H}$ = 7.8 Hz, 2H; Dipp *meta*-C**H**), 6.35 (s, 2H; IDipp C**H**), 2.70 (sept, $^3J_{H–H}$ = 6.8 Hz, 2H; Dipp C**H**(CH$_3$)$_2$), 2.66 (sept, $^3J_{H–H}$ = 6.8 Hz, 2H; Dipp C**H**(CH$_3$)$_2$), 1.89 (d, $^3J_{H–Rh}$ = 2.1 Hz, 15H; Cp* C$_5$(C**H**$_3$)$_5$), 1.55 (d, $^3J_{H–H}$ = 6.8 Hz, 6H; Dipp CH(C**H**$_3$)$_2$), 1.53 (d, $^3J_{H–H}$ = 6.8 Hz, 6H; Dipp CH(C**H**$_3$)$_2$), 1.11 (d, J = 6.8 Hz, 12H; Dipp CH(C**H**$_3$)$_2$), 0.89 (dd, $^2J_{H–P}$ = 8.6 Hz, $^3J_{H–Rh}$ = 0.9 Hz, 9H; PMe$_3$ P(C**H**$_3$)$_3$).

^{13}C{^{1}H} NMR (151 MHz, C$_6$D$_6$): δ(ppm) 195.55 (d, $^3J_{C–P}$ = 4.0 Hz; IDipp [HCN(Dipp)]$_2$**C**Au), 145.91 (Dipp *ortho*-**C**), 145.86 (Dipp *ortho*-**C**), 135.26 (Dipp *ipso*-**C**), 130.47 (Dipp *para*-**C**), 124.22 (Dipp *meta*-**C**), 124.15 (Dipp *meta*-**C**), 122.63 (IDipp [H**C**N(Dipp)]$_2$CAu), 96.12 (dd, $^1J_{C–Rh}$ = 3.8 Hz, $^2J_{C–P}$ = 3.8 Hz; Cp* **C**$_5$(CH$_3$)$_5$), 29.08 (Dipp **C**H(CH$_3$)$_2$), 24.79 (s, (Dipp CH(**C**H$_3$)$_2$)), 24.70 (s, (Dipp CH(**C**H$_3$)$_2$)), 24.16 (Dipp CH(**C**H$_3$)$_2$), 19.50 (d, $^1J_{C–P}$ = 27.6 Hz, P(**C**H$_3$)$_3$), 10.92 (Cp* C$_5$(**C**H$_3$)$_5$).

^{31}P{^{1}H} NMR (162 MHz, C_6D_6): δ(ppm) 94.66 (dd, $^{1}J_{P–Rh}$ = 33.8 Hz, $^{2}J_{P–P}$ = 4.7 Hz; μ$_2$-C≡**P**), –5.02 (dd, $^{1}J_{P–Rh}$ = 209.0 Hz, $^{2}J_{P–P}$ = 4.7 Hz; **P**Me$_3$).

Raman: 1175 cm^{-1} (m, *v*(C≡P)).

UV-vis: λ_{max} (nm) 372, 411.

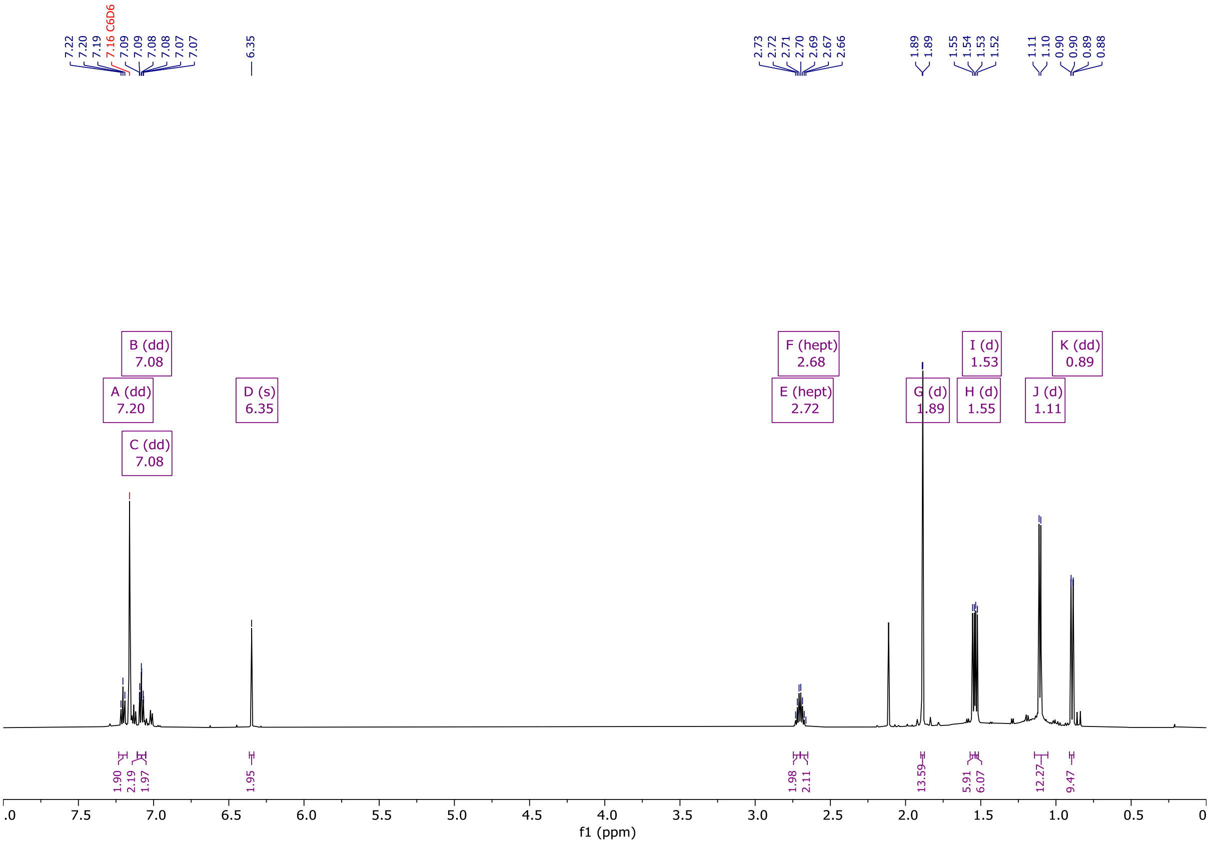

Figure S10. ^{1}H NMR spectrum of **3** in C_6D_6.

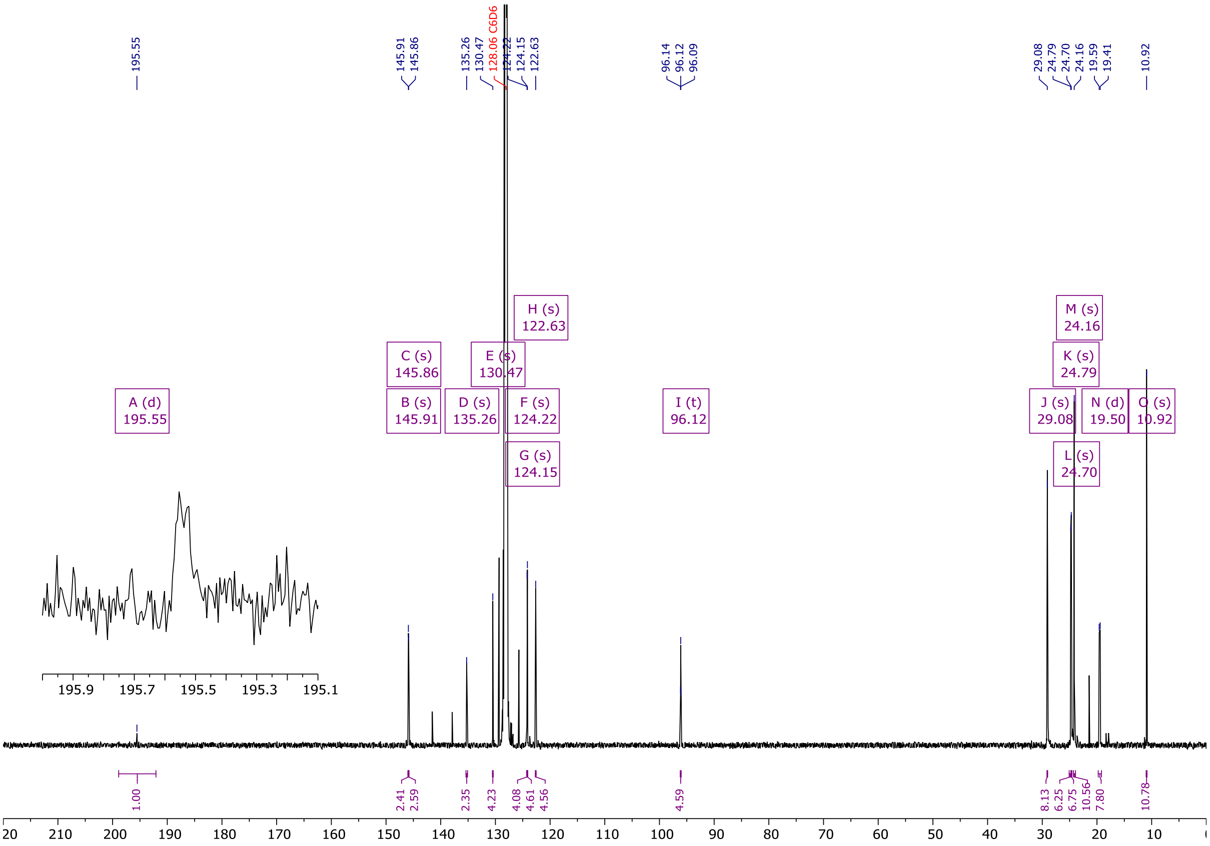

Figure S11. $^{13}C\{^1H\}$ NMR spectrum of **3** in C_6D_6.

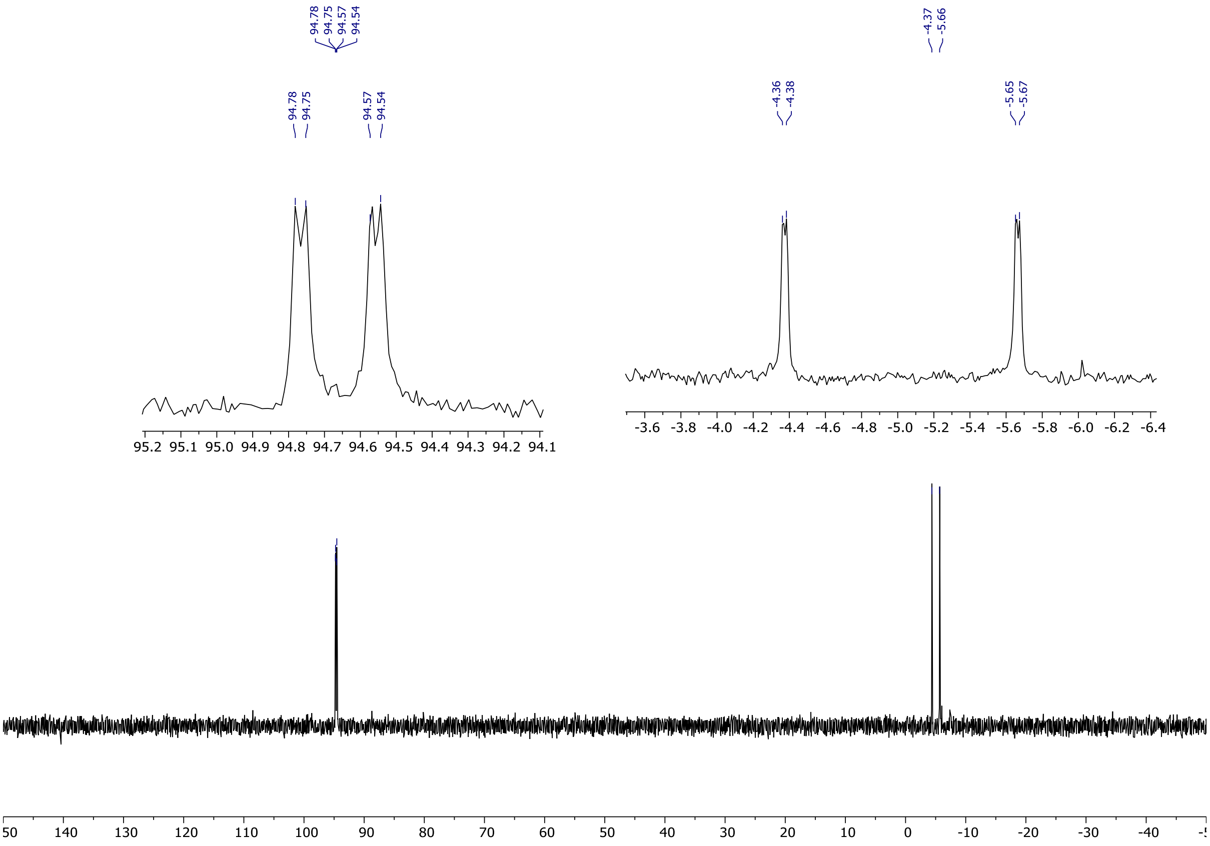

Figure S12. $^{31}P\{^1H\}$ NMR spectrum **3** in C_6D_6.

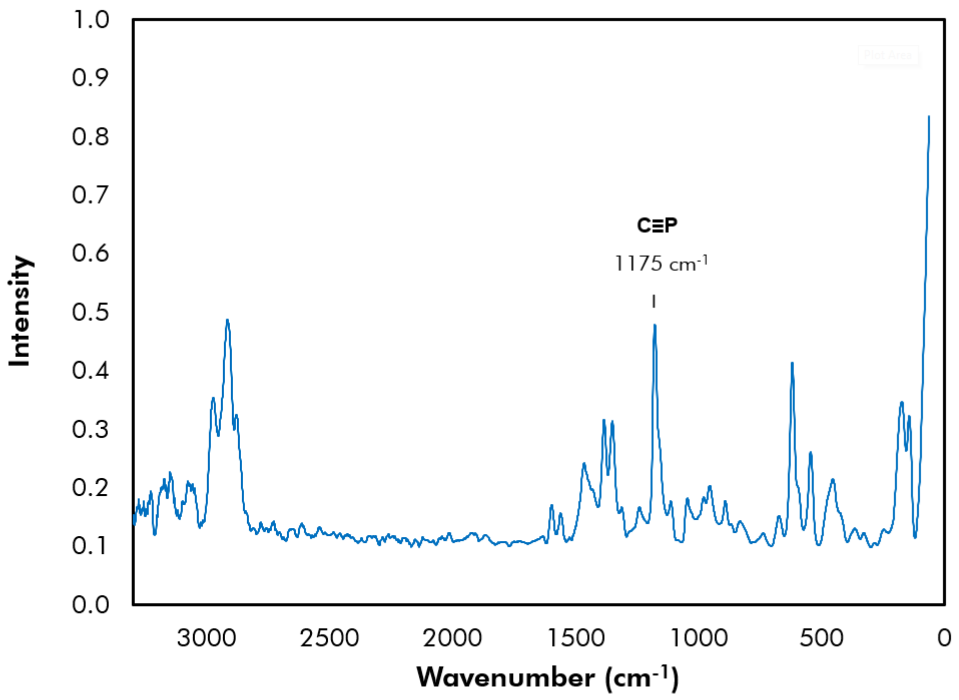

Figure S13. Dispersive Raman spectrum of **3**.

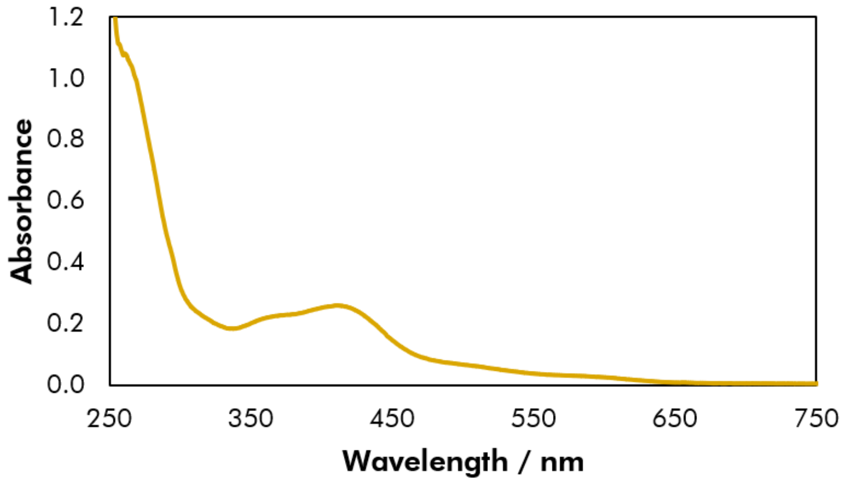

Figure S14. UV-visible absorption spectrum of **3**.

1.2.4 Synthesis of Au(IDipp)(μ_3-C≡P)[Rh(Cp*)(PMe$_3$)][W(CO)$_5$] (4)

W(CO)$_5$(THF) (approx. 21 mg, 0.05 mmol) was generated in situ by degassing a THF (0.5 mL) solution of W(CO)$_6$ (19 mg, 0.05 mmol) in a quartz flask, then irradiating with a 125 W mercury lamp for 4 h under a static vacuum. The resulting bright yellow solution was degassed to remove liberated CO, then cooled to –35 °C. The solution was then added dropwise to a solution of **3** (51 mg, 0.05 mmol) in THF (0.5 mL) at –35 °C. The reaction

mixture was allowed to warm to room temperature and stirred for 2 h. All volatiles were then removed under vacuum, and the resulting orange-red residue extracted with hexane (10 mL). The mixture was filtered to remove any particulates, then concentrated by slow evaporation. Storage at –35 °C for 3 days afforded red crystals of Au(IDipp)(μ_3-C≡P)[Rh(Cp*)(PMe_3)][W$(CO)_5$] (**4**). The supernatant was decanted, concentrated further by slow evaporation, then stored at –35 °C for 3 days to afford a second crop (Combined yield: 48 mg, 0.04 mmol, 70% yield).

^{1}H NMR (600 MHz, C_6D_6): δ(ppm) 7.21 (t, $^3J_{H–H}$ = 7.8 Hz, 2H; Dipp *para*-C**H**), 7.08 (dd, $^3J_{H–H}$ = 7.8 Hz, $^4J_{H–H}$ = 1.4 Hz, 2H; Dipp *meta*-C**H**), 7.06 (dd, $^3J_{HH}$ = 7.8 Hz, $^4J_{H–H}$ = 1.4 Hz, 2H; Dipp *meta*-C**H**), 6.30 (s, 2H; IDipp C**H**), 2.63 (sept, $^3J_{H–H}$ = 6.8 Hz, 2H; Dipp C**H**(CH_3)$_2$), 2.59 (sept, $^3J_{H–H}$ = 6.8 Hz, 2H; Dipp C**H**(CH_3)$_2$), 1.72 (d, $^3J_{H–Rh}$ = 2.4 Hz, 15H; Cp* C_5(C**H**$_3$)$_5$), 1.49 (d, $^3J_{H–H}$ = 6.8 Hz, 6H; Dipp CH(C**H**$_3$)$_2$), 1.47 (d, $^3J_{H–H}$ = 6.8 Hz, 6H, Dipp CH(C**H**$_3$)$_2$), 1.10 (d, $^3J_{H–H}$ = 6.8 Hz, 6H; Dipp CH(C**H**$_3$)$_2$), 1.09 (d, $^3J_{H–H}$ = 6.8 Hz, 6H; Dipp CH(C**H**$_3$)$_2$), 0.84 (dd, $^2J_{H–P}$ = 8.9 Hz, $^3J_{H–Rh}$ = 0.9 Hz, 9H; PMe_3 P(C**H**$_3$)$_3$).

^{13}C{^{1}H} NMR (151 MHz, C_6D_6): δ(ppm) 202.52 (d, $^1J_{C–W}$ = 126.0 Hz, $^2J_{C–P}$ = 25.3 Hz; W(CO_5) *ax.* **C**O), 198.01 (d, $^2J_{C–P}$ = 6.9 Hz; W(CO_5) *eq.* **C**O), 193.94 (d, $^3J_{C–P}$ = 6.4 Hz; IDipp [HCN(Dipp)]$_2$**C**Au), 145.82 (Dipp *ortho*-**C**), 145.82 (Dipp *ortho*-**C**), 134.91 (Dipp *ipso*-**C**), 130.59 (Dipp *para*-**C**), 124.28 (Dipp *meta*-**C**), 124.19 (Dipp *meta*-**C**), 122.67 (IDipp [H**C**N(Dipp)]$_2$CAu), 97.43 (dd, $^2J_{C–P}$ = 4.0 Hz, $^1J_{C–Rh}$ = 4.0 Hz; Cp* **C**$_5$(CH_3)$_5$), 29.08 (Dipp **C**H(CH_3)$_2$), 29.05 (Dipp **C**H(CH_3)$_2$), 24.78 (Dipp CH(**C**H$_3$)$_2$), 24.76 (Dipp CH(**C**H$_3$)$_2$), 24.11 (Dipp CH(**C**H$_3$)$_2$), 24.05 (Dipp CH(**C**H$_3$)$_2$), 18.76 (d, $^1J_{C–P}$ = 29.1 Hz; PMe_3 P(**C**H$_3$)$_3$), 10.43 (Cp* C_5(**C**H$_3$)$_5$).

^{31}P{^{1}H} NMR (162 MHz, C_6D_6): δ(ppm) 48.54 (dd, $^1J_{P–W}$ = 174.7 Hz, $^1J_{P–Rh}$ = 61.9 Hz, $^2J_{P–P}$ = 13.4 Hz; μ_3-C≡**P**), –3.86 (dd $^1J_{P–Rh}$ = 193.6 Hz, $^2J_{P–P}$ = 13.4 Hz; **P**Me$_3$).

ATR-FTIR: 2059 cm^{-1} (m, *v*(C≡O) $A_1^{(1)}$), 1972 cm^{-1} (w, *v*(C≡O) B_1), 1928 cm^{-1} (s, *v*(C≡O) E), 1881 cm^{-1} (m, *v*(C≡O) $A_1^{(2)}$).

Raman: 2062 cm^{-1} (m, *v*(C≡O) $A_1^{(1)}$), 1977 cm^{-1} (m, *v*(C≡O) B_1), 1883 cm^{-1} (m, *v*(C≡O) $A_1^{(2)}$), 1186 cm^{-1} (s, v(C≡P)).

UV-vis: λ_{max} (nm) 379, 414.

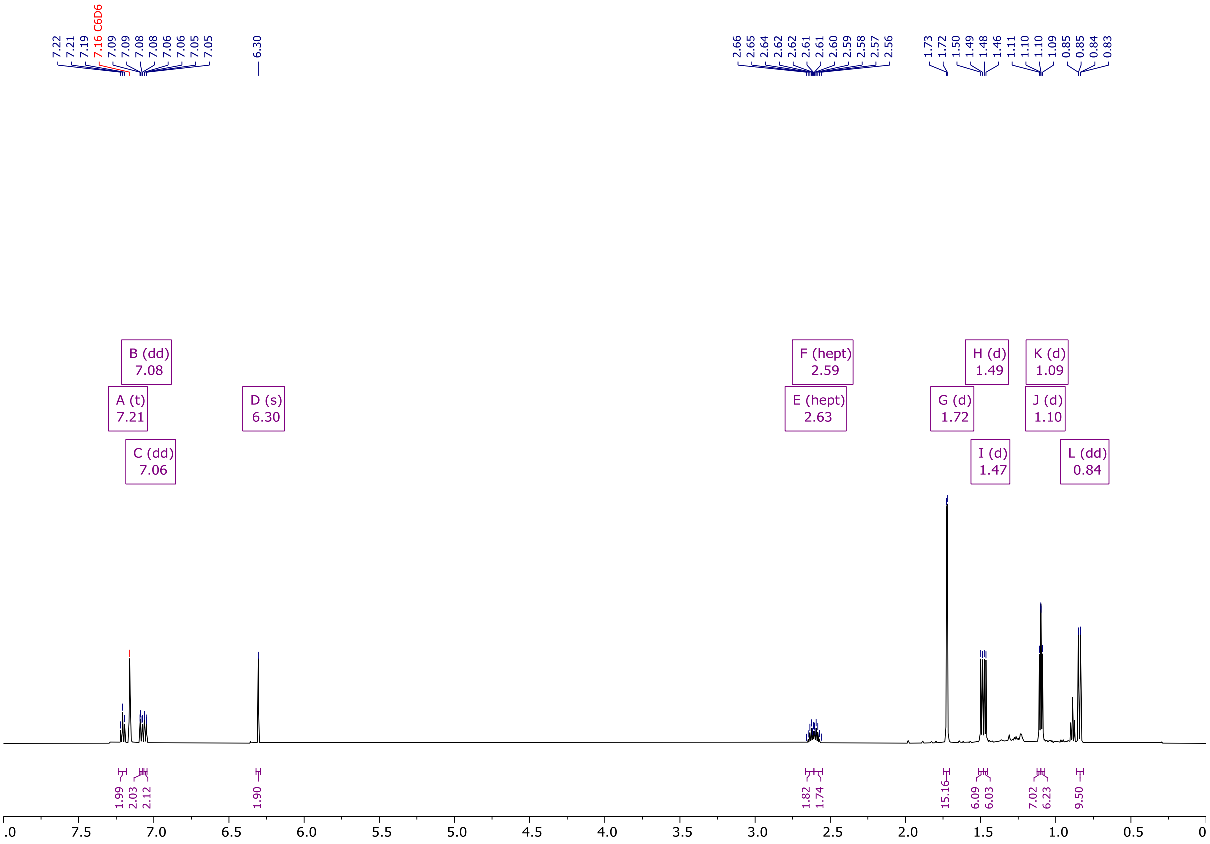

Figure S15. ^{1}H NMR spectrum of **4** in C_6D_6.

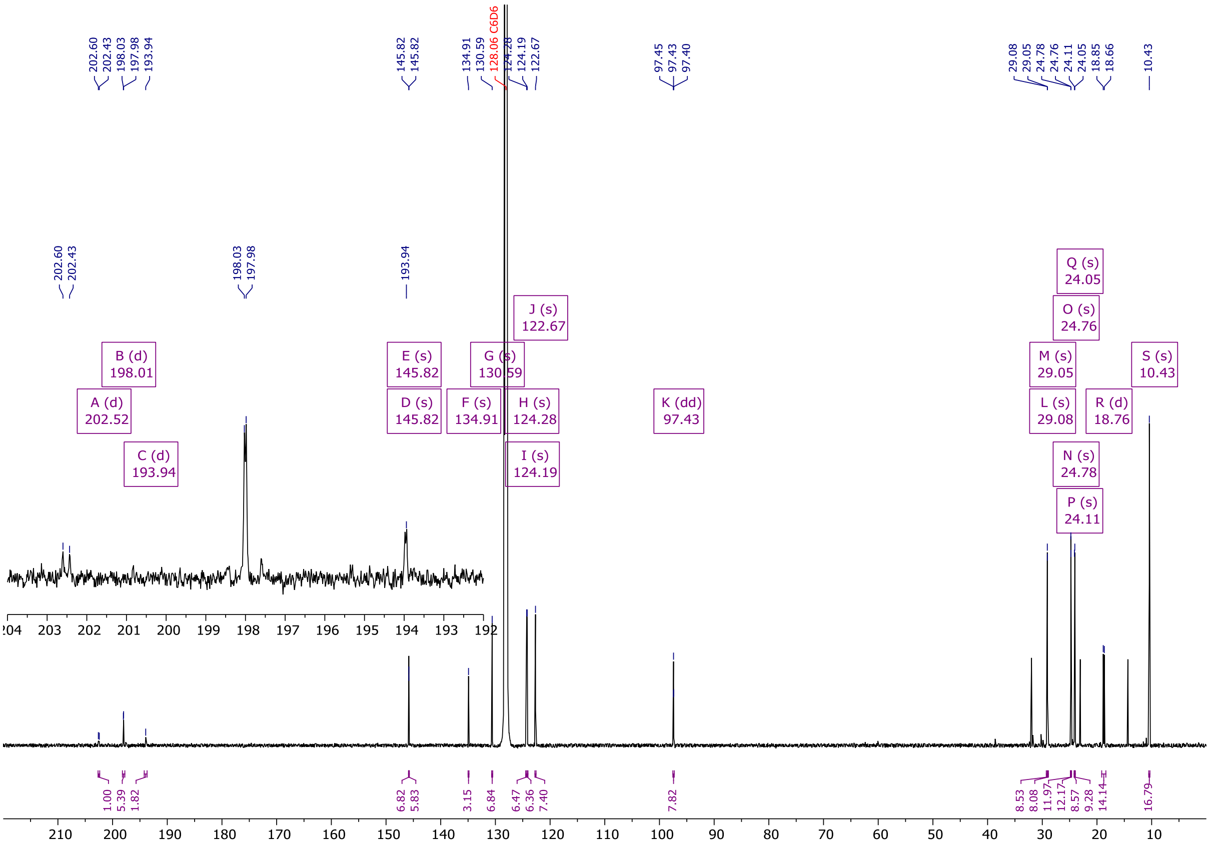

Figure S16. $^{13}C\{^{1}H\}$ NMR spectrum of **4** in C_6D_6.

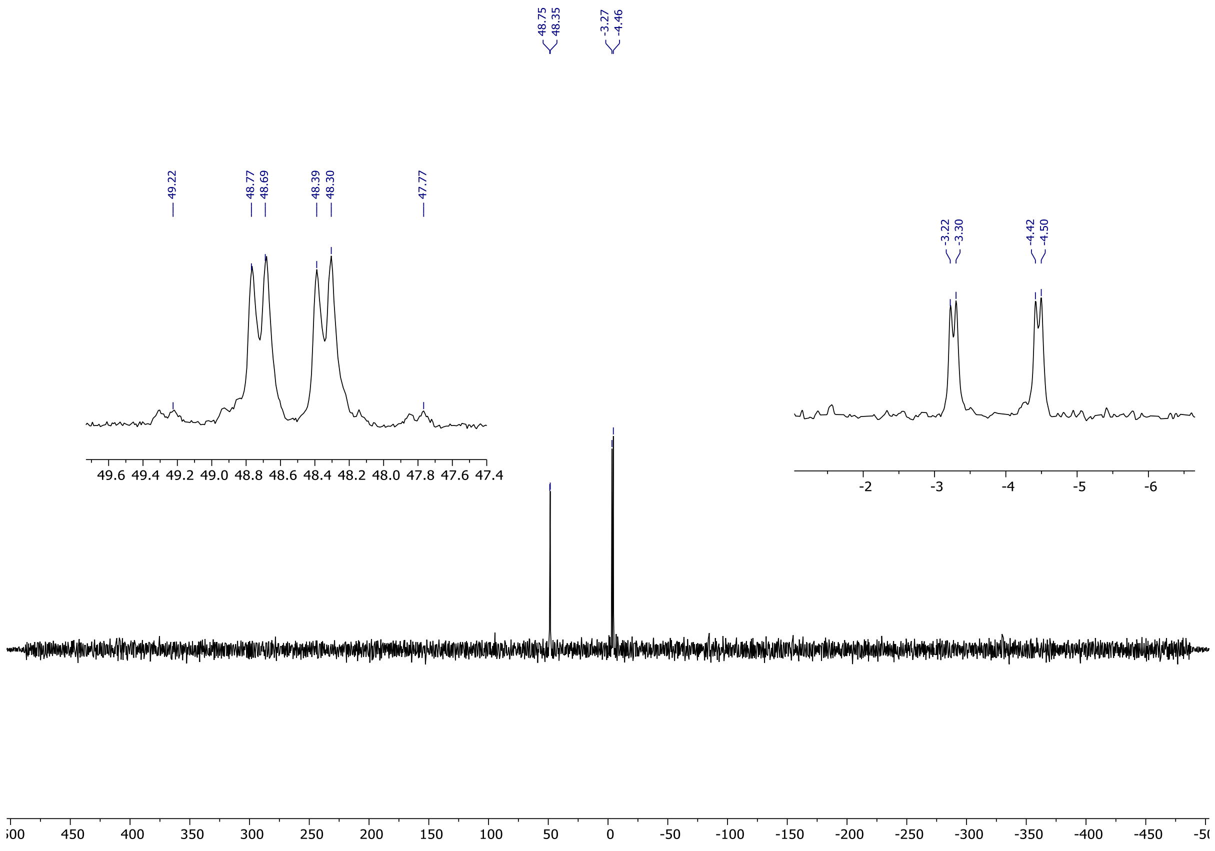

Figure S17. $^{31}P\{^{1}H\}$ NMR spectrum of **4** in C_6D_6.

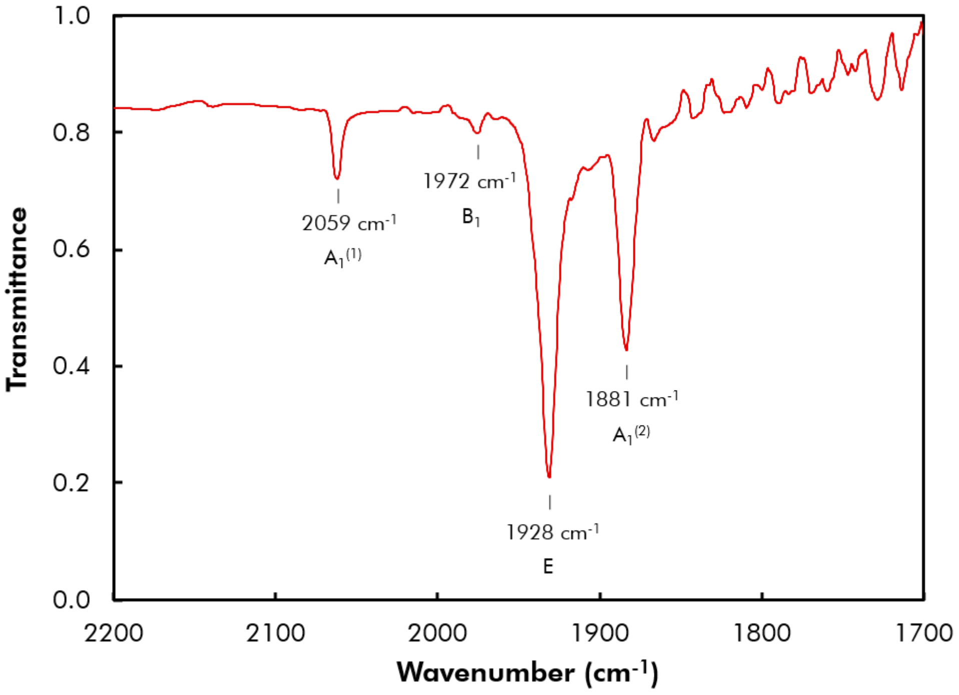

Figure S18. ATR-FTIR spectrum of **4.**

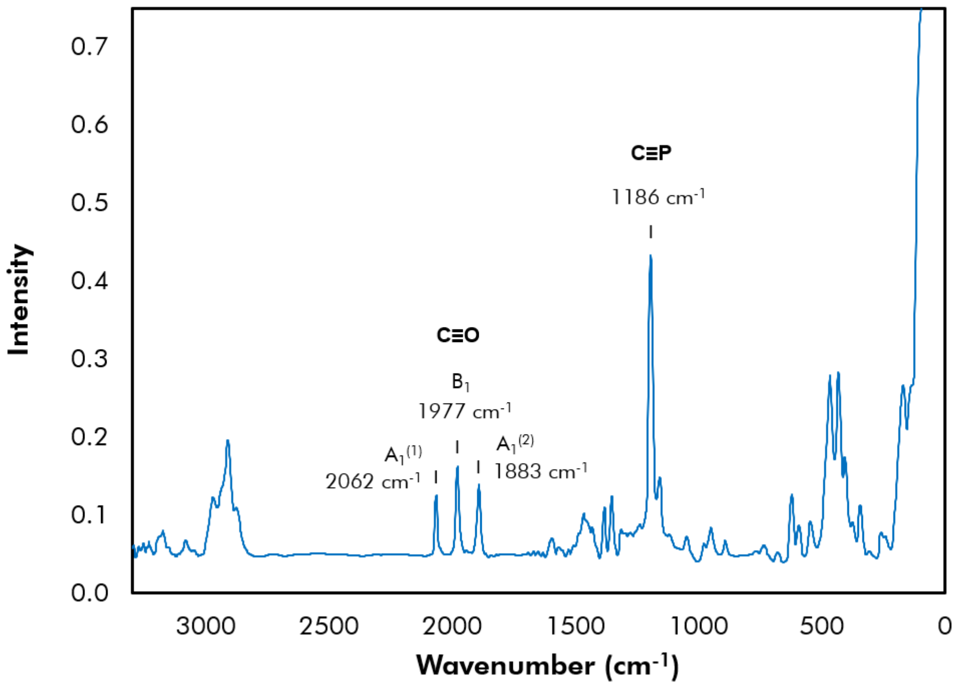

Figure S19. Dispersive Raman spectrum of **4**.

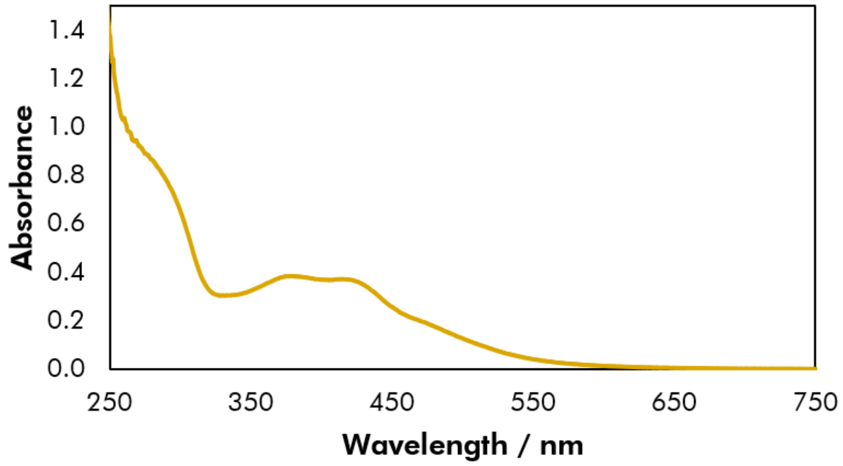

Figure S20. UV-visible absorption spectrum of **4**.

1.3 Additional characterization

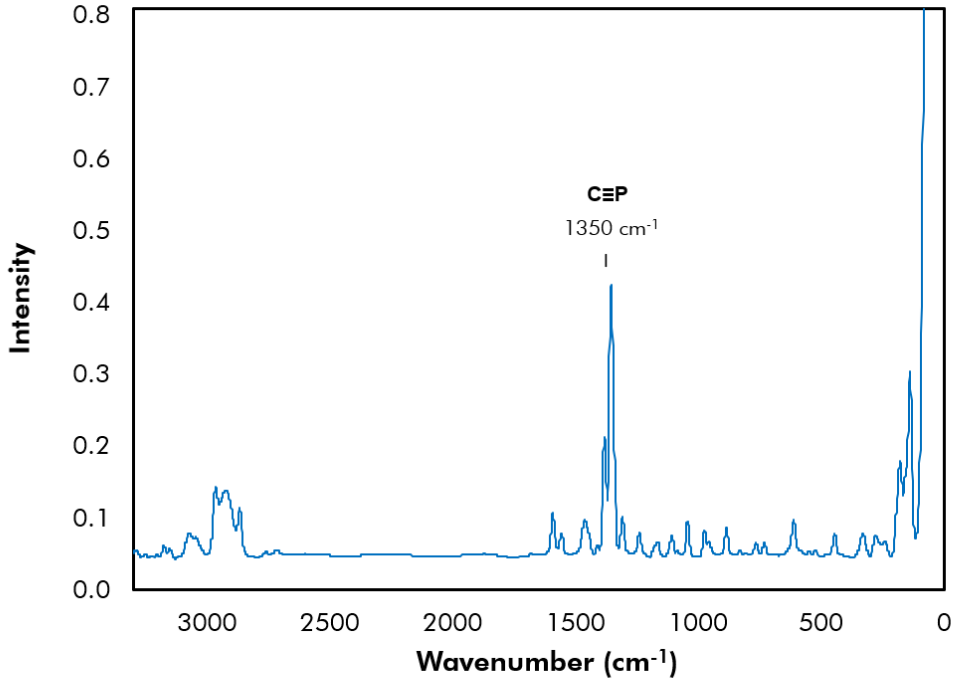

Figure S21. Dispersive Raman spectrum of **A**.

2. Single crystal X-ray diffraction data

Single-crystal X-ray diffraction data were collected using an Oxford Diffraction Supernova dual-source diffractometer equipped with a 135 mm Atlas CCD area detector. Crystals were selected under Paratone-N oil, mounted on micromount loops and quench-cooled using an Oxford Cryosystems open flow N_2 cooling device. Data were collected at 150 K using mirror monochromated Cu Kα (λ = 1.54184 Å) radiation and processed using the CrysAlisPro package, including unit cell parameter refinement and inter-frame scaling (which was carried out using SCALE3 ABSPACK within CrysAlisPro).[6] Structures were subsequently solved using direct methods.[7]

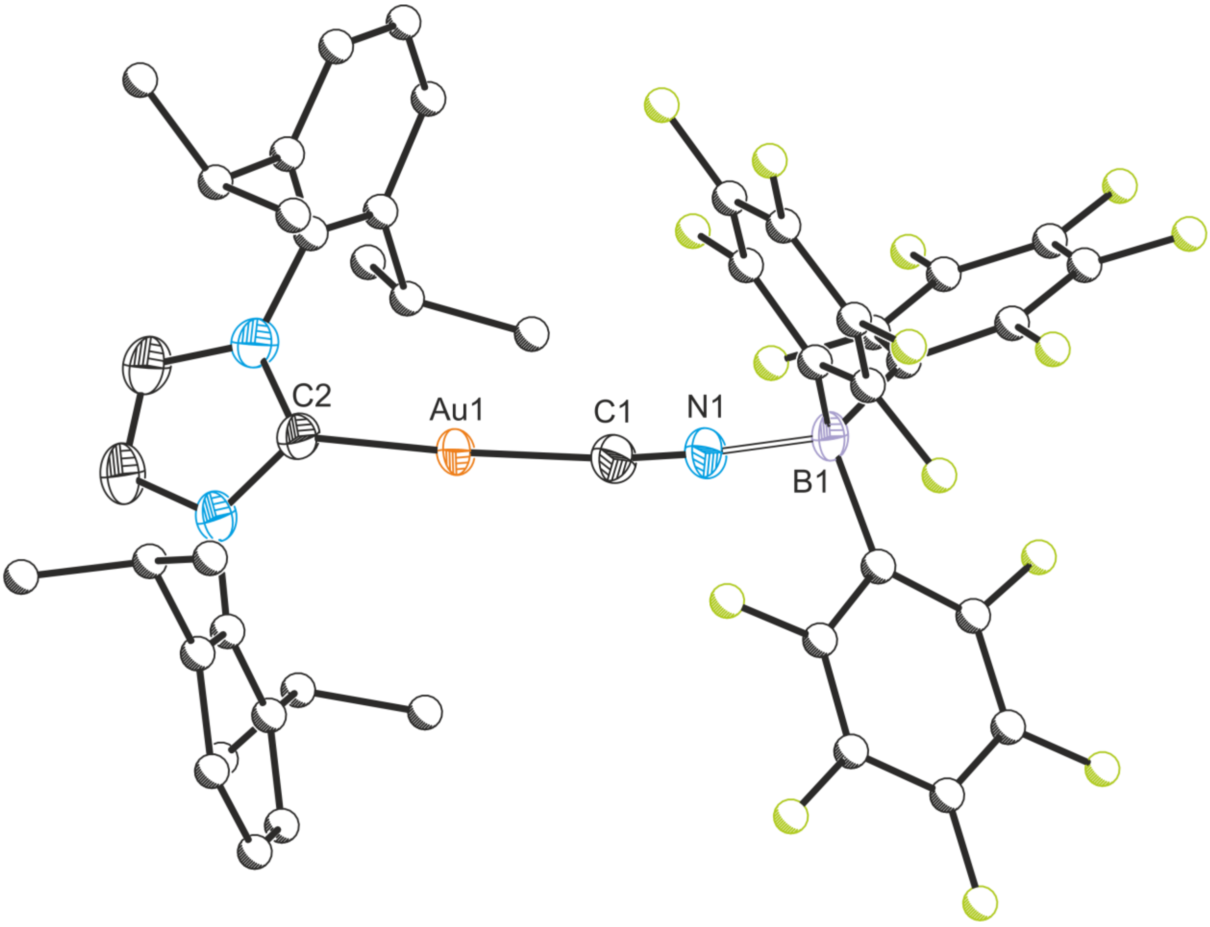

Figure S22. Solid-state structure of **1**. Anisotropic displacement ellipsoids set at 50% probability. Hydrogens omitted for clarity. Atoms of Dipp and C_6F_5 groups displayed as spheres of arbitrary radius. Selected bond lengths (Å) and angles (°): Au1−C1 1.976(3), Au1−C2 2.005(2), C1−N1 1.140(3), N1−B1 1.566(3); C1-Au1-C2 176.28(10), Au1-C1-N1 174.1(2), C1-N1-B1 175.7(3).

Table S1. Selected X-ray data collection and refinement parameters for **1**·tol, **2**·2C_6H_6, **3**·hex and **4**·0.5hex.

	**1**·tol	**2**·2C_6H_6	**3**·hex	**4**·0.5hex
Formula	$C_{53}H_{44}AuBF_{15}N_3$	$C_{62}H_{88}AuN_6NiP$	$C_{47}H_{74}AuN_2P_2Rh$	$C_{49}H_{67}AuN_2O_5P_2RhW$
CCDC	2171578	2171579	2171580	2171581
Fw [g mol^{-1}]	1215.69	1204.03	1028.89	1309.71
Crystal system	triclinic	monoclinic	orthorhombic	monoclinic
Space group	*P*−1	*P*2$_1$/*c*	*Pbca*	*P*2$_1$/*c*
a (Å)	12.9001(2)	10.2343(1)	17.2262(1)	20.4976(2)
b (Å)	14.2097(2)	29.7930(2)	20.7164(2)	10.5365(1)
c (Å)	16.5212(2)	20.1214(1)	27.5711(2)	25.2448(2)
α (°)	67.537(1)	90	90	90
β (°)	83.160(1)	95.889(1)	90	97.291(1)
γ (°)	64.777(1)	90	90	90
V (Å^3)	2528.37(7)	6102.85(8)	9839.16(13)	5408.11(9)
Z	2	4	8	4
Radiation, λ (Å)	Cu Kα, 1.54184	Cu Kα, 1.54184	Cu Kα, 1.54184	Cu Kα, 1.54184
Temp (K)	150(2)	150(2)	150(2)	150(2)
ρ_{calc} (g cm^{-3})	1.597	1.310	1.389	1.609
μ (mm^{-1})	6.306	5.388	9.072	12.172
Reflections collected	63325	147019	90393	67962
Independent reflections	10499	12731	10313	11265
Parameters	718	648	494	566
R(int)	0.0292	0.0477	0.0483	0.0447
R1/wR2,[a] I ≥ 2σI (%)	2.53/6.17	3.33/8.54	3.70/9.02	3.22/8.38
R1/wR2,[a] all data (%)	2.55/6.18	3.86/8.91	4.04/9.38	3.88/8.38
GOF	1.040	1.056	1.030	1.092

[a] $R1 = [\Sigma||F_o| - |F_c||]/\Sigma|F_o|$; $wR2 = \{[\Sigma w[(F_o)^2 - (F_c)^2]^2]/[\Sigma w(F_o^2)^2]\}^{1/2}$; $w = [\sigma^2(F_o)^2 + (AP)^2 + BP]^{-1}$, where $P = [(F_o)^2 + 2(F_c)^2]/3$ and the A and B values are 0.0278 and 3.53 for **1**·tol, 0.0433 and 9.73 for **2**·2C_6H_6, 0.0420 and 42.11 for **3**·hex, and 0.0396 and 9.93 for **4**·0.5hex.

3. Computational details

3.1. General computational methods

Density functional theory (DFT) and coupled-cluster theory calculations were performed using the ORCA 5.0.2 software package.[8–10] All methods were used as implemented. Geometries were optimized using the PBEh-3c method and the def2-mSVP basis set.[11] Analytical frequency calculations were carried out to verify all geometries were true minima (NIMAG = 0). Single point calculations on all compounds and fragments were performed using the ωB97X-D3 functional[12] and the Resolution of Identity approximation (RIJCOSX),[13,14] and corrected for relativistic effects using the zeroth order regular approximation (ZORA). The segmented all-electron relativistically contracted basis set SARC-ZORA-TZVP was used for all heavy atoms (Rh, W, and Au), and the relativistically contracted triple-zeta basis set ZORA-def2-TZVP was used for all other atoms, along with the SARC/J auxiliary basis set.[15,16] For HCN and HCP, calculations were performed using the coupled-cluster singles and doubles method with perturbative triples correction (CCSD(T)), and the triple zeta atomic natural orbitals basis set ano-pVTZ.[17] Natural Bond Order analysis and Natural Population Analysis were carried out using the NBO 7.0 program.[18] Energy decomposition analysis was carried out using the ETS-NOCV approach[19,20] as implemented in ORCA 5.0.2, with difference densities generated by Multiwfn 3.8.[21]

3.2. Energy decomposition analysis

3.2.1 M–(η²-(C≡P))

Table S2. Energy decomposition analysis of M–(η²-(C≡P)) interaction energies in **2**, **3**, and **4**. Energies in kcal/mol.

	ΔE_{tot}	ΔE_{steric}	ΔE_{disp}	ΔE_{oi}	ΔE^{σ}_{orb}	ΔE^{π}_{orb}
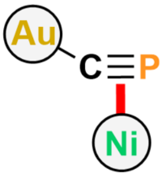 (**2**)	–84.99	123.12	–75.38	–132.74	–11.95	–102.17
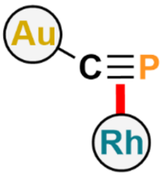 (**3**)	–62.72	98.51	–57.92	–103.32	–25.70	–59.20
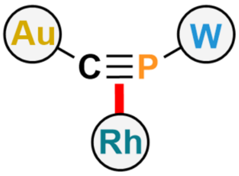 (**4**)	–71.82	121.34	–69.52	–123.65	–24.72	–78.93

$\Delta E_{tot} = \Delta E_{steric} + \Delta E_{disp} + \Delta E_{oi} = \Delta E_{steric} + \Delta E_{disp} + \Sigma\Delta E^{n}_{orb}$

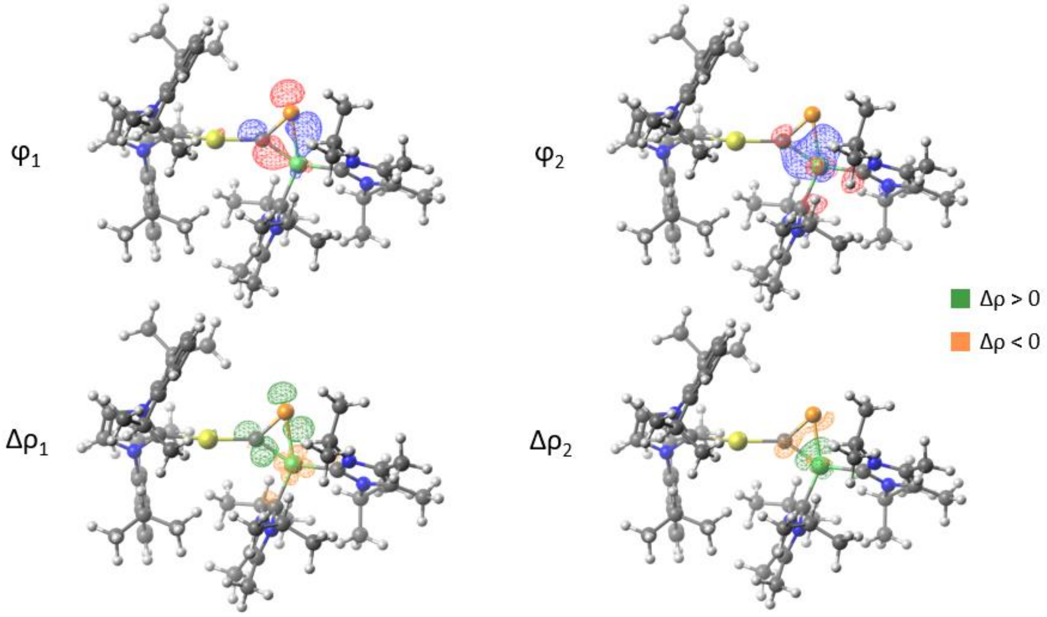

Figure S23. Contours of the two NOCVs with the largest eigenvalues, φ_1 and φ_2, (v > 0), as well as their contributions to the deformation density, $\Delta\rho_1$ and $\Delta\rho_2$, for the Ni–(η^2-(C≡P)) interaction in **2**. φ_1 corresponds to π back-donation and φ_2 corresponds to σ donation.

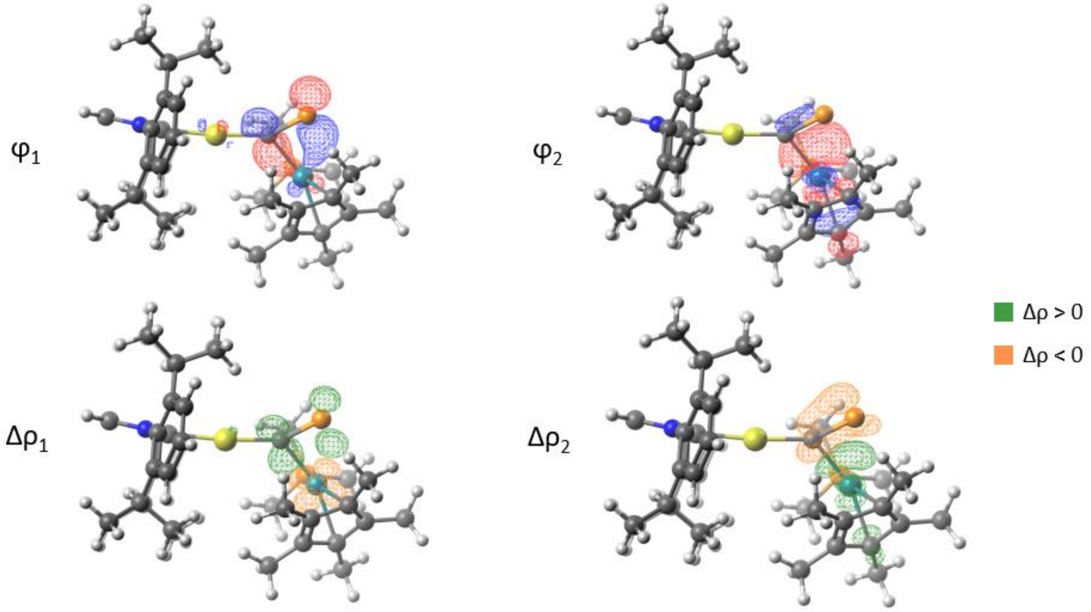

Figure S24. Contours of the two NOCVs with the largest eigenvalues, φ_1 and φ_2, (v > 0), as well as their contributions to the deformation density, $\Delta\rho_1$ and $\Delta\rho_2$, for the Rh–(η^2-(C≡P)) interaction in **3**. φ_1 corresponds to π back-donation and φ_2 corresponds to σ donation.

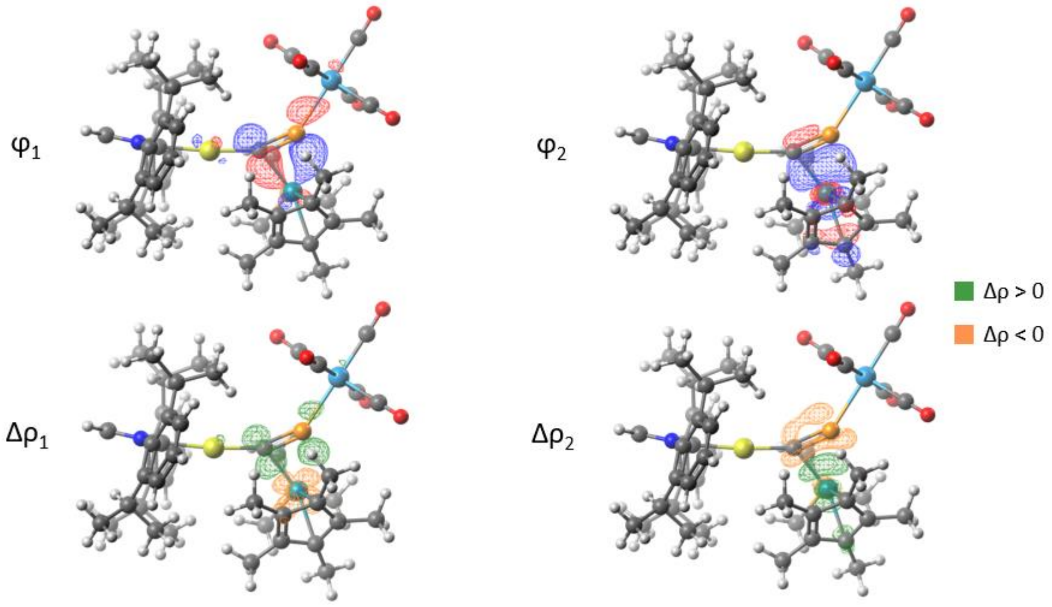

Figure S25. Contours of the two NOCVs with the largest eigenvalues, φ_1 and φ_2, ($v > 0$), as well as their contributions to the deformation density, $\Delta\rho_1$ and $\Delta\rho_2$, for the Rh–(η^2-(C≡P)) interaction in **4**. φ_1 corresponds to π back-donation and φ_2 corresponds to σ donation.

3.3.2 L–W(CO)$_5$

Table S3. Energy decomposition analysis of L–W(CO)$_5$ interaction energies. Energies in kcal/mol.

L =	ΔE_{tot}	ΔE_{steric}	ΔE_{disp}	ΔE_{oi}	ΔE^{σ}_{orb}	ΔE^{π}_{orb}
**3**	–37.73	83.13	–74.33	–46.53	–30.54	–11.50
PF_3	–44.16	22.52	–4.64	–62.03	–29.67	–30.27
PH_3	–39.7387	8.98	–4.44	–44.28	–28.46	–14.03
PMe_3	–50.50	6.19	–8.85	–47.85	–32.54	–12.29
PPh_3	–44.19	22.82	–20.51	–46.49	–29.70	–12.37
PCA	–40.28	13.44	–6.55	–47.17	–29.21	–15.38
PCO^-	-55.25	–3.40	–4.32	–47.54	–34.60	–

$\Delta E_{tot} = \Delta E_{steric} + \Delta E_{disp} + \Delta E_{oi} = \Delta E_{steric} + \Delta E_{disp} + \Sigma\Delta E^{n}_{orb}$

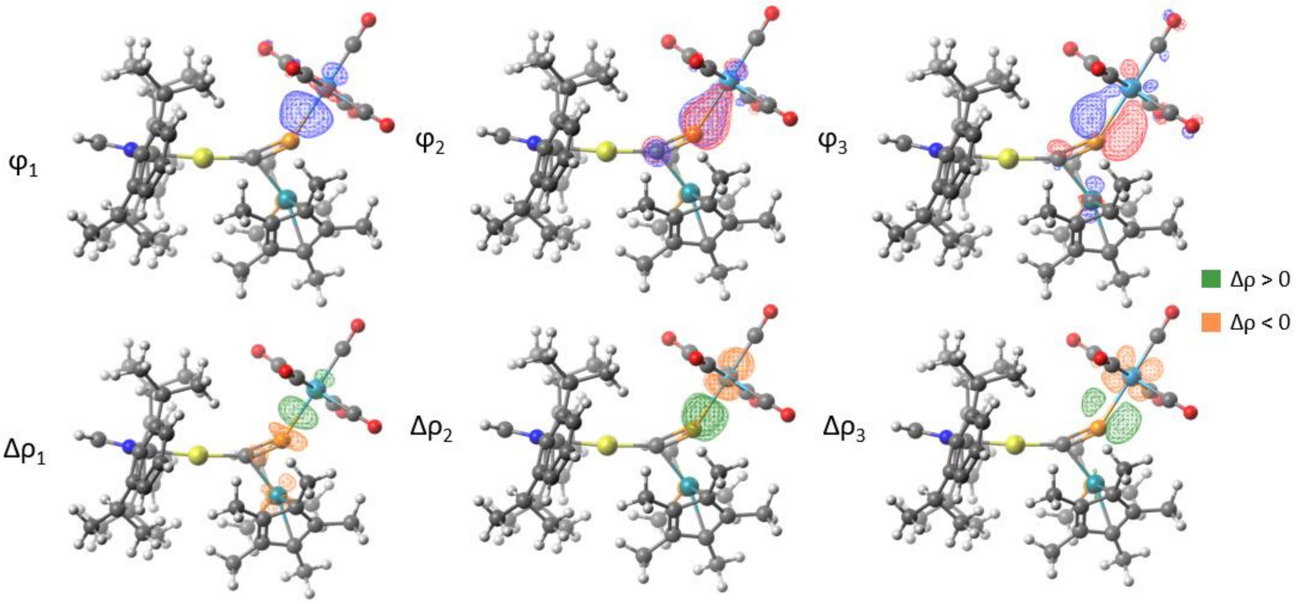

Figure S26. Contours of the three NOCVs with the largest eigenvalues, φ_1, φ_2, and φ_3, (v > 0), as well as their contributions to the deformation density, $\Delta\rho_1$, $\Delta\rho_2$, and $\Delta\rho_3$, for the (κP-(C≡P))–W interaction in **4**. φ_1 corresponds to σ donation, whereas φ_2 and φ_3 correspond to π back-donation.

3.3. Data analysis

3.3.1 M–(η²-(C≡P))

Table S4. (C≡P) Raman stretching frequencies (in cm^{-1}), C–P bond lengths (in Å), Au–C–P bond angles (in degrees), and calculated σ donation and π back-donation contributions to the M–(η^2-(C≡P)) interaction (as percentages of the total interaction energy) for **A**, **2**, **3**, and **4**.

	ν(C≡P)	C–P	∠Au–C–P	%(ΔE^{σ}_{orb})	%(ΔE^{π}_{orb})
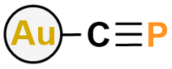 (**A**)	1350	1.552	178.0	0	0
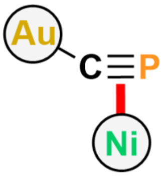 (**2**)	1125	1.642	146.3	9.00	89.53
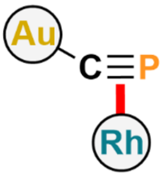 (**3**)	1175	1.631	151.4	24.87	66.73
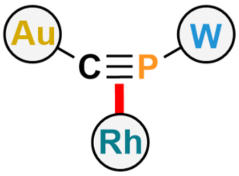 (**4**)	1186	1.605	155.4	20.00	63.83

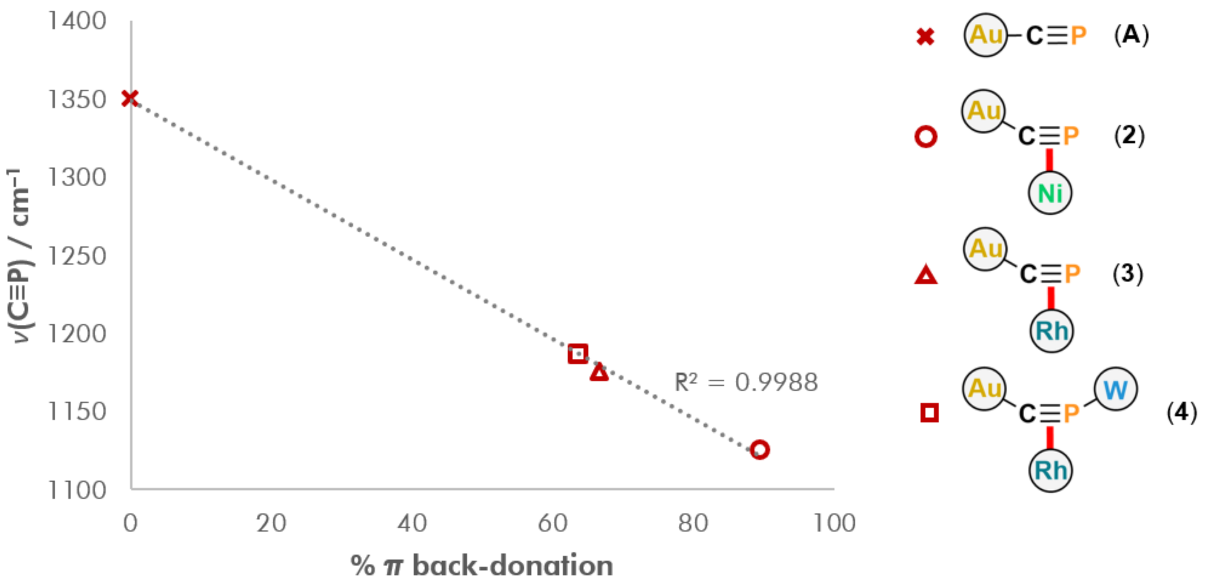

Figure S27. Correlation of the calculated π back-donation contributions to the M–(η^2-(C≡P)) interaction with experimentally determined (C≡P) Raman stretching frequencies for **A**, **2**, **3**, and **4**.

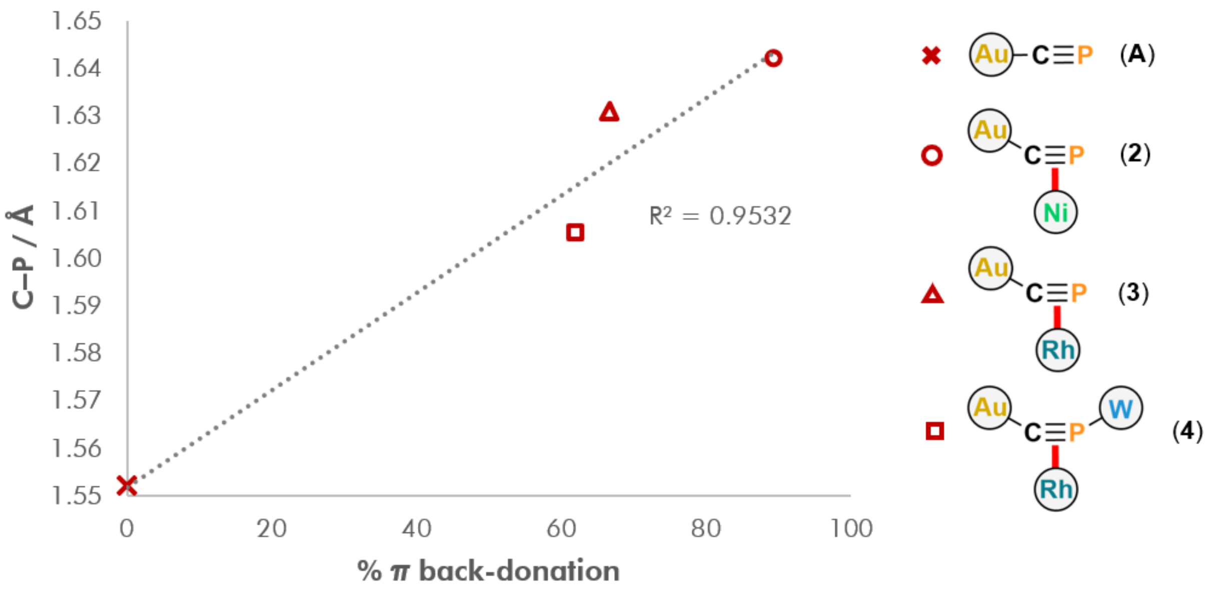

Figure S28. Correlation of the calculated π back-donation contributions to the M–(η^2-(C≡P)) interaction with experimentally determined C–P bond lengths for **A**, **2**, **3**, and **4**.

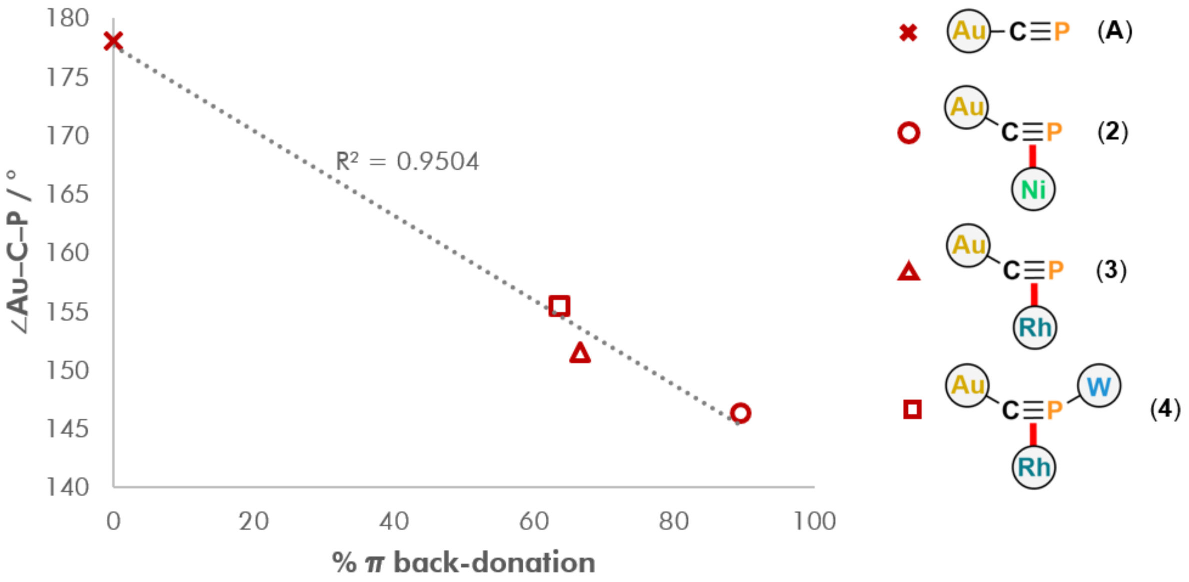

Figure S29. Correlation of the calculated π back-donation contributions to the M–(η^2-(C≡P)) interaction with experimentally determined Au–C–P bond angles for **A**, **2**, **3**, and **4**.

3.3.2 L–W(CO)$_5$

Table S5. Reported (C≡O) IR stretching modes (in cm^{-1}) and $^{1}J_{PW}$ NMR coupling constants (in Hz), as well as calculated σ donation and π back-donation contributions to the L–W(CO)$_5$ interaction (as percentages of the total interaction energy) for **3**, and literature known phosphines.

L=	v(CO) $A_1^{(1)}$	v(CO) B_1	v(CO) E	$^{1}J_{P–W}$	%(ΔE^{σ}_{orb})	%(ΔE^{π}_{orb})
**3**	2059	1972	1928	175	72.65	27.35
PCO^-	2052	1960	1906	52	–	–
PBu_3	2070	1976	1934	227	–	–
PMe_3	2071	–	1937	227	72.58	27.42
$PPhBu_2$	2071	1975	1937	235	–	–
$PPh_2{}^tBu$	2072	1979	1937	240	–	–
PPh_2Bu	2073	1979	1938	250	–	–
$PPh_2{}^iPr$	2074	1979	1937	240	–	–
PPh_2Et	2073	1979	1938	240	–	–
PPh_2Me	2073	1979	1939	245	–	–
PPh_3	2075	1980	1942	280	70.60	29.40
PCA	2077	–	–	–	65.52	33.03
$P(OBu)_3$	2077	1977	1944	390	–	–
$P(OEt)_3$	2078	1976	1945	391	–	–
PH_3	2080	1981	1952	–	66.97	33.03
$P(OMe)_3$	2081	1980	1952	398	–	–
$P(OPh)_3$	2083	–	1958	415	–	–
PBr_3	2092	1991	1981	398	–	–
PCl_3	2094	1994	1980	426	–	–
PF_3	2103	–	1983	485	49.50	50.50

3.4. Other computational analyses

3.4.1 Electronic structure of Au(IDipp)(C≡P) (A) and Au(IDipp)(C≡N) (B)

The canonical molecular orbitals of the gold(I) cyaphide and cyanide complexes **A** and **B** are heavily delocalized, making interpretation of the cyaphido and cyanido ligand orbitals difficult. NBO analysis was used to localize the frontier molecular orbitals onto the –C≡P and –C≡N fragments. The cyaphide π bonding NBOs (2.00 e⁻, –8.33 eV; 1.99 e⁻, –8.33 eV) are higher in energy than the cyanide π bonding NBOs (2.00 e⁻, –10.06 eV; 2.00 e⁻, –10.06 eV). Conversely, the cyaphide π* antibonding NBOs (0.03 e⁻, +3.60 eV; 0.03 e⁻, +3.62 eV) are appreciably lower in energy than the corresponding cyanide π* antibonding NBOs (0.02 e⁻, +6.67 eV; 0.02 e⁻, +6.70 eV). Compared to its relatively high energy π bonding NBOs, the cyaphide phosphorus lone pair NBO (1.98 e⁻, –12.36 eV) is substantially lower in energy. In contrast, the cyanide nitrogen lone pair NBO (1.97 e⁻, –11.70 eV) is much closer in energy to its π bonding NBOs.

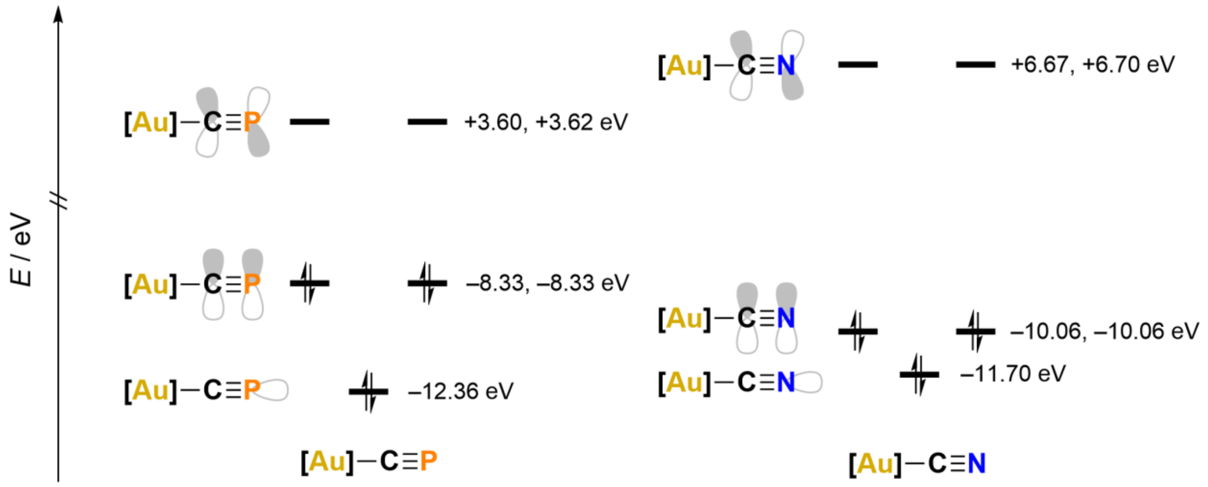

Figure S30. Energy level diagram for the frontier NBOs of the –C≡P and –C≡N fragments in **A** and **B**.

Due to the well-established analogy between gold and hydrogen,[22–25] the electronic structures of the HCP and HCN molecules were investigated using coupled-cluster theory to serve as far simpler model systems that do not require orbital localization methods. These results are broadly similar to the DFT/NBO analysis of **A** and **B**, showing a smaller HOMO–LUMO gap for HCP, and a larger difference in energy between the π bonding HOMO and the σ lone pair HOMO–1.

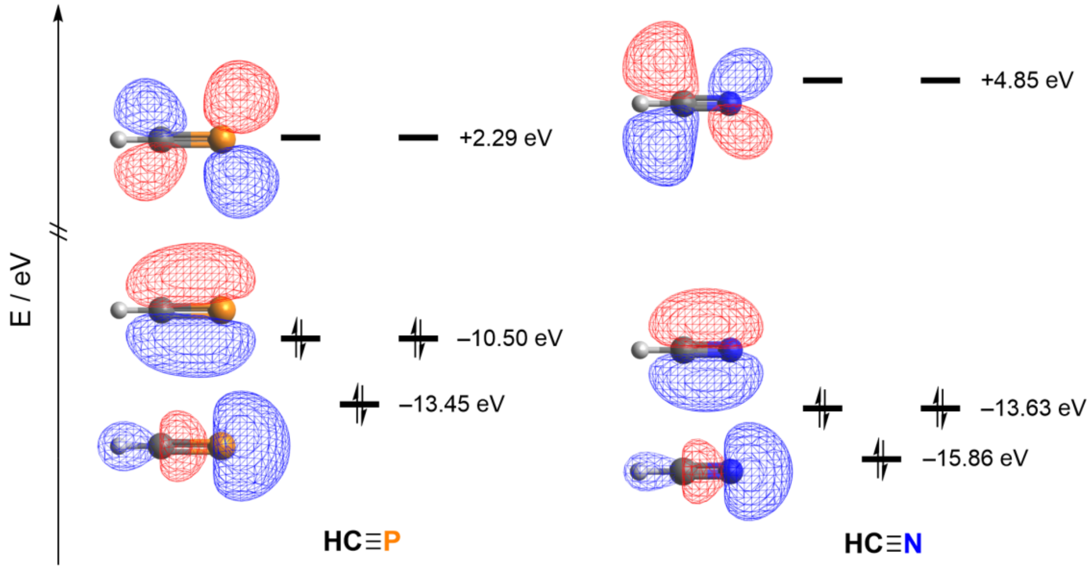

Figure S31. Energy level diagram for the frontier molecular orbitals of HCP and HCN.

3.4.1 Comparison of η^1:η^2-(μ-C≡P) and η^1:η^1-(μ-C≡P) coordination modes

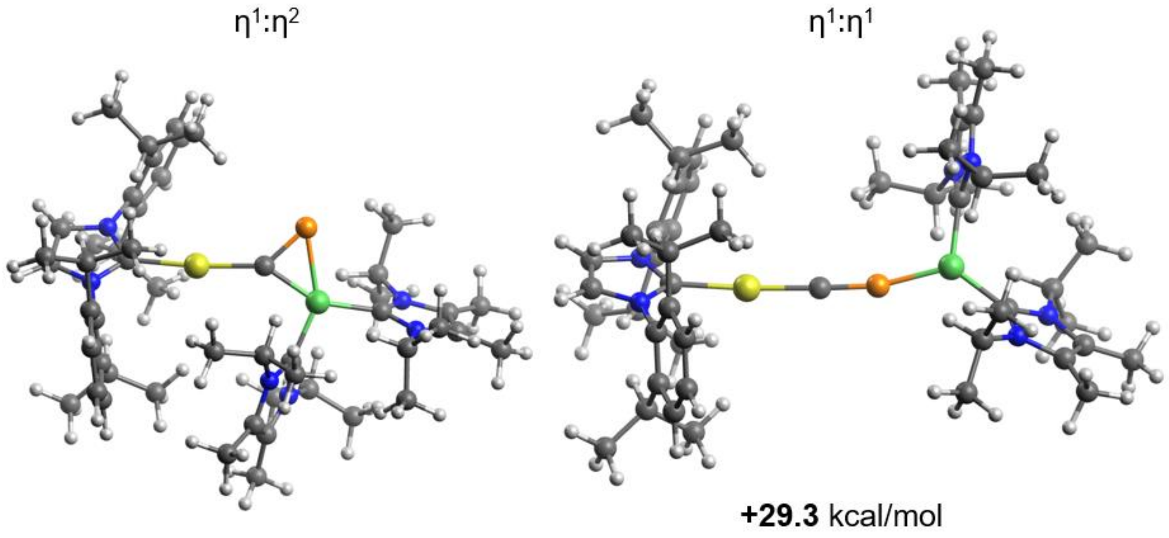

Figure S32. DFT optimized structures of **2** and its η^1:η^1-(μ-C≡P) isomer.

3.5. XYZ coordinates

Au(IDipp)(C≡P) (**A**)

Au	0.06694094260074	-0.14374016487139	0.01015739785581
C	0.09803054530780	-0.27293688821636	1.97942651796782
P	0.11975745759881	-0.36370884273651	3.53014973157349
N	1.07706909545065	0.05172703185345	-2.86948384717708
C	0.66215838564784	0.10252594087239	-4.18136057820324
C	-0.68656844105899	0.07362270054306	-4.15500963920177
N	-1.04745621167137	0.00600820278253	-2.82829547600554
C	0.03157818344293	-0.01017536894792	-2.02778927128707
H	1.35627353117796	0.15606065784698	-5.00205631508009
H	-1.41431359088410	0.09566733956503	-4.94746630861164
H	-1.56323460000553	4.18010240514616	-1.12146716510433
H	-2.59483048220160	4.34690831155226	-3.45643195509635
C	-2.09701071192356	3.24171454267765	-0.96158772652492
H	-1.50141915827094	2.62524361847136	-0.28730799939821
H	-1.36082183380190	2.37086723078191	-2.75223993449154
C	-3.13604950538522	3.42139103262479	-3.25552528459437
C	-2.33973340761480	2.53661526041403	-2.29666741128778
H	-3.03486473365062	3.47955444653307	-0.45669349444639
H	-4.10711878674556	3.69518832446932	-2.84051758781666
H	-3.31079762910806	2.92073675081036	-4.20851513733601
C	-3.02039724131309	1.20048150947882	-2.08797279113579
C	-2.38881023816230	-0.01850009401607	-2.34361982171577
H	-4.84710061787172	2.07662266926202	-1.38736494218420
C	-4.32325784081607	1.15383263408935	-1.60313128447843
C	-2.99909739190415	-1.25586705428071	-2.13157136055964
C	-4.95815476646555	-0.05678913968521	-1.38399914666326
C	-4.30267167149625	-1.24753722253292	-1.64610983225993
H	-5.97054397533234	-0.07245684580324	-1.00166515311375
H	-4.81066060496711	-2.18602782693275	-1.46323756615343
H	-1.72841934966705	-2.93554337483244	-0.34577287677381
C	-2.23891516708198	-3.44803977883750	-1.16152690084173

H	-3.23190452721601	-3.73315848086019	-0.81066728184016
H	-1.69138471304617	-4.36679419082962	-1.37738079756121
C	-2.30679594432980	-2.57195456787866	-2.41192080971568
H	-1.27606269001173	-2.37465985682491	-2.71425266626317
C	-2.98812049704752	-3.30604145916872	-3.56741853940169
H	-4.01148599366782	-3.58901106972549	-3.31480682015438
H	-2.44507570016136	-4.22039227302825	-3.81097948968029
H	-3.02738466309444	-2.68975225814533	-4.46660111157458
H	3.18489261724694	-2.58154521861706	-4.60190782708115
C	3.15723056458612	-3.19702131914993	-3.70179842058187
H	2.63546428181188	-4.12374457439942	-3.94475433281761
H	4.18576294165764	-3.45699064706302	-3.44626638752686
H	1.41846311244796	-2.31531957100261	-2.85476966764884
C	3.10398624376294	-1.13918551228637	-2.27288602839855
C	2.45457802257363	-2.47758372578574	-2.54997493515917
C	4.42267381192051	-1.08684545744142	-1.83325117334271
C	5.03912762319393	0.12495159268121	-1.57326079873532
H	4.97323044893848	-2.00730195913798	-1.68537650872141
H	6.06464870059040	0.14402334392257	-1.22792786241786
C	2.41436076275276	-3.34861355914389	-1.29456196353531
H	1.89741369401269	-4.28645222811912	-1.50437511156973
H	3.41643672668345	-3.59814432301182	-0.94192716664568
H	1.88753439274419	-2.84777447104825	-0.48192841715668
H	3.17422742090785	3.07366707711488	-4.28798792216837
H	2.43064846105248	4.46023071632599	-3.48957593287337
C	3.01468207889466	3.55427972375681	-3.32211204161618
H	3.98853827384688	3.86139068875921	-2.93800208385955
C	2.28909060644638	2.62595276089745	-2.34826412590498
H	1.30152839634167	2.42684962916600	-2.77058934065409
C	2.43658596615624	0.07573383594669	-2.43753432114054
C	3.02853601278292	1.31503685531569	-2.18464082243208
H	1.49441074914470	4.22302022173659	-1.12226442428601
C	2.06912387987498	3.30399370309742	-0.99555821556257

H	3.01498172708591	3.57085247277821	-0.52112196291542
C	4.34935400587027	1.31251897059103	-1.74850204780393
H	4.84562354766328	2.25131232521292	-1.53640442316349
H	1.52173947375620	2.65783479728355	-0.30844505794355

Au(IDipp)(η^1:η^2-μ-C≡P)Ni($^{Me}I^iPr_2$)$_2$ (**2**)

Au	-0.00360742988066	-0.11106169707624	-0.05472408743370
C	0.02385723686985	-0.25168144015977	1.92470043774743
P	0.89202766981939	0.39680575958645	3.17162811569137
Ni	-0.42703989760525	-1.34827787571541	3.38187438121120
N	1.31709093778269	0.38103856505876	-2.73483848910652
N	-0.76684277392477	0.11979256510180	-3.05818428575177
N	1.04195473582828	-2.71516659053540	5.53448485781366
N	-0.90883876738517	-2.17502953737554	6.21118471671358
N	-2.98543723041596	-2.07611860751049	2.10262218431097
N	-1.72338187276538	-3.78589843126265	2.29994787473258
C	0.16754975114289	0.09933629020216	-2.08586646194415
C	1.10481653351249	0.58673151036881	-4.08019345529100
H	1.90340189253170	0.82853797218507	-4.75953950333918
C	-0.21511281945793	0.41622796402072	-4.28772385480957
H	-0.80652385772110	0.48261746641231	-5.18396622753891
C	2.60060184234291	0.40659240811558	-2.10996467505736
C	2.99018575692697	1.54869810655028	-1.40782736927442
C	4.25642866378171	1.53989701801307	-0.83320190842188
H	4.59604798123259	2.40433982591502	-0.27812998058095
C	5.08571462031629	0.43995651021648	-0.94712712454161
H	6.06494434348603	0.45430027311002	-0.48677911226331
C	4.66453707160520	-0.68385761080949	-1.63529052714850
H	5.31972299977812	-1.54281372604323	-1.69915694145902
C	3.40983507474534	-0.72585135945630	-2.23095162048971
C	2.11168751895740	2.77432076894297	-1.27805441284240
H	1.09348216057890	2.50669662675516	-1.56405749803705
C	2.03831824346031	3.27851737900441	0.16198361454910

H	1.30891930406857	4.08643710408106	0.23525915531524
H	1.73230228801209	2.48925638110888	0.84960311707454
H	2.99335193275736	3.67868776995374	0.50726128149232
C	2.58754358771124	3.86819569132675	-2.23532239434855
H	1.93225736006436	4.73912968800578	-2.18057807626912
H	3.59831153578215	4.19993293033460	-1.99040290910819
H	2.59531508634306	3.52227294927806	-3.27017084434633
C	2.93712628348897	-1.99147750588901	-2.91531575268061
H	2.09078061409407	-1.75238859283980	-3.56296723008790
C	2.44251142511833	-2.99846835147759	-1.87386207797727
H	2.05767382924134	-3.89654275358692	-2.36140377049173
H	3.25236392033363	-3.29906385268177	-1.20730554844599
H	1.64901102265421	-2.57613145579602	-1.25607115454537
C	4.00931735784233	-2.60863943221483	-3.81155449819972
H	4.40122176938069	-1.88697000456574	-4.52895307685141
H	4.85177718243448	-2.99799138466355	-3.23878621402209
H	3.59317220736247	-3.44651101136090	-4.37219195889842
C	-2.15638037607599	-0.12180124609472	-2.85752914798097
C	-3.01119564705221	0.97688377918558	-2.74008450778635
C	-4.37141227078362	0.72099500636853	-2.60888072060651
H	-5.06459149911073	1.54655013750096	-2.50796354428027
C	-4.85441315193768	-0.57539307886625	-2.59438906232834
H	-5.91775675376619	-0.75341904030815	-2.49856047133027
C	-3.98105953894618	-1.64382285730166	-2.68775539781625
H	-4.36823016586643	-2.65485676823945	-2.66067367572121
C	-2.61078918721182	-1.44119034938162	-2.81512889724393
C	-2.50216923612898	2.40149385584778	-2.69845828341439
H	-1.44045829823516	2.40560462052395	-2.95215827276684
C	-2.62185760148180	2.96114332930134	-1.28020859800950
H	-2.23049419723103	3.97876507211054	-1.23584087526304
H	-3.66216329484612	2.98969048054554	-0.95074692436572
H	-2.06048402852377	2.35419705819239	-0.56900004879068
C	-3.20714484873107	3.29612205635257	-3.71659597942388

H	-2.76337386954535	4.29230335711676	-3.71155696163403
H	-3.12376359295318	2.89808061260899	-4.72851444722114
H	-4.26852388801132	3.41613032168388	-3.49457536167364
C	-1.68245813449113	-2.63389148699899	-2.87391102107285
H	-0.65638214653447	-2.27871204274256	-2.98010918141161
C	-1.74908376538752	-3.42298856490568	-1.56667161169055
H	-1.52165528074838	-2.79117409990799	-0.70768017329517
H	-2.73776691137016	-3.85752945892160	-1.40593121232545
H	-1.03029301841645	-4.24504206355633	-1.58011513186323
C	-1.98355593630031	-3.52129132855790	-4.08068812406238
H	-1.27126480161199	-4.34615441201673	-4.13196430642173
H	-2.98282456556580	-3.95659122725232	-4.02754688504490
H	-1.91638174933849	-2.96337180678286	-5.01524347030175
C	-0.09555861193170	-2.08871968009120	5.13047984772286
C	0.93927544452032	-3.17215419625393	6.83985424123782
C	-0.29924256252667	-2.83525675634127	7.26905916024564
C	2.00234378795691	-3.90518278767004	7.57761339032055
H	2.95926461536988	-3.38413667898921	7.55867007234837
H	1.72443084450033	-4.01476459213628	8.62401372065885
H	2.16760104574687	-4.90809821465271	7.18084256088651
C	-0.90932880916786	-3.07348260464264	8.60490252830883
H	-1.91307618579996	-3.49276975123980	8.54172162968619
H	-0.30805685265679	-3.78387617806567	9.16916799389628
H	-0.97270140114311	-2.16174671679301	9.20135795739730
C	2.24575609079033	-2.71525330557445	4.70640084300542
H	1.90053991514888	-2.37163878508672	3.73155335592276
C	2.85441029274296	-4.09974509030523	4.51625480868568
H	3.54220143167597	-4.07053173634689	3.67098226774843
H	3.42342963610276	-4.44400158864682	5.37904285909506
H	2.09019189758806	-4.84515876891089	4.29368578556495
C	3.25906314944113	-1.68837721222818	5.19120011274374
H	4.08383435815437	-1.62198496617658	4.48089787946124
H	2.79473966936531	-0.70493404425612	5.24892418304177

H	3.68124823463117	-1.93842262945976	6.16575513404473
C	-2.26957137153767	-1.66035432190724	6.16348728872719
H	-2.30016252964186	-1.10787502806169	5.22534475866551
C	-3.29296914506905	-2.78384086478845	6.07453236345151
H	-4.29880110791816	-2.37277002514680	5.97814866203792
H	-3.10334959763986	-3.40841086448559	5.20128547450108
H	-3.28964351179830	-3.42107169802287	6.95908642071568
C	-2.57722921585122	-0.66473774835093	7.27365864089968
H	-3.50812795502896	-0.14719847985563	7.03962238590054
H	-2.70880367148023	-1.13315955278556	8.24826771933281
H	-1.79136841506615	0.08626596136928	7.35111818558848
C	-1.76914883671929	-2.45673455869048	2.56960251078814
C	-2.88756191069196	-4.23029368134840	1.69106210457909
C	-3.68667643928727	-3.14495708266639	1.56585430117891
C	-3.14272427434386	-5.61903699390862	1.22516060795887
H	-2.90883542580572	-6.36852155954759	1.98139759022504
H	-4.19590444689732	-5.74250885567381	0.97748672550597
H	-2.57372572760584	-5.87053245271960	0.32802969701490
C	-5.02224924915042	-3.05958625821593	0.91780478171491
H	-5.74511888546615	-2.50181494614434	1.51229492702481
H	-4.96845522965148	-2.58302574961238	-0.06312198339043
H	-5.43545981815894	-4.05668083911583	0.77250273321995
C	-0.56507727080907	-4.58977661259921	2.66678145601453
H	0.17585769208106	-3.85038245274843	2.96209357254506
C	0.03209357719909	-5.35849875096770	1.49518069605433
H	1.02857457816766	-5.70735928356664	1.76878287808501
H	-0.54690961107642	-6.23697196981912	1.21181295929557
H	0.13541830767443	-4.71278139976971	0.62344614073969
C	-0.84695751270245	-5.46699146281044	3.87776156693476
H	-1.18644006910094	-4.86281652685848	4.71871390493050
H	-1.60087832011242	-6.22953019014359	3.67764166903319
H	0.06165371151666	-5.98497076837776	4.18778098870975
C	-3.44009090341562	-0.68970271862671	2.18853547271398

H	-2.55741069259838	-0.14557600545735	2.52279137921352
C	-3.83008429801091	-0.10242689483881	0.84089623136994
H	-3.87713183361541	0.98294487196848	0.93257884442152
H	-3.08188844822121	-0.33185362591365	0.08347269094485
H	-4.80390215761075	-0.43713107935883	0.48446652287873
C	-4.53761415849312	-0.50788831810125	3.22908418607676
H	-4.74171545241753	0.55471704001130	3.36472432697510
H	-5.47502680579036	-0.98538247583362	2.94062806081205
H	-4.23775889439230	-0.91467765077281	4.19291582468555

Au(IDipp)(η^1:η^1-μ-C≡P)Ni($^{Me}I^{i}Pr_2$)$_2$

Au	-0.02020637499893	-0.48656448620479	0.24851186949969
C	-0.32577674225775	-0.88195643885770	2.14217674123762
P	-0.59291397245466	-1.11497996826455	3.66012399944324
Ni	-0.69321698387250	-1.06023109184860	5.68439132131070
N	1.47952375493118	0.33687064559001	-2.27589952748670
N	-0.61458174074167	0.32419583247109	-2.63873672331080
N	1.79157401504144	0.28484094190022	6.45093121139038
N	0.08998123036518	1.49884644124344	6.89223273900794
N	-3.13891680503665	-2.16192462291688	6.89758589690666
N	-1.38629246632199	-2.89902870803603	7.87102378152191
C	0.30013959630590	0.05596532970728	-1.68529264050753
C	1.30694681761368	0.77486368912073	-3.57031541825021
H	2.13433310264330	1.04757699448634	-4.20153427766190
C	-0.02116588780204	0.76600398244071	-3.80090109005428
H	-0.59172172157074	1.03070626691123	-4.67361819045440
C	2.74343885317552	0.18906644041043	-1.63438681393706
C	3.21704650246325	1.22972883521558	-0.83340777137480

C	4.46070074848138	1.05999594016103	-0.23625192630357
H	4.86219808994997	1.84470044576598	0.39252865167681
C	5.18943650254852	-0.10060442900934	-0.42384584994070
H	6.15287023068446	-0.21546433499893	0.05542776718788
C	4.68565889541339	-1.12114873855189	-1.21034205551964
H	5.26021821701553	-2.03131956623124	-1.33015462080314
C	3.44877804906904	-0.99917784236962	-1.83208644210352
C	2.44092711895186	2.50737133797333	-0.60280146846069
H	1.45304590810200	2.40484968753972	-1.05518487685084
C	2.21820314034855	2.76800331711422	0.88673453022885
H	1.58532780044053	3.64629667091762	1.02183239410203
H	1.72428946495594	1.92351844536156	1.36733491765410
H	3.15616865985908	2.95751322963926	1.41155123372395
C	3.13489191810695	3.68962878246147	-1.28022020518016
H	2.55887794516351	4.60518928680966	-1.13816855291988
H	4.13064233348436	3.86022084627390	-0.86685188765949
H	3.24725242911543	3.52906758842892	-2.35331782646211
C	2.89065931205917	-2.15103965794708	-2.63908336663837
H	1.96446675700021	-1.83032019197699	-3.12005126938231
C	2.53452351648512	-3.31953777493942	-1.71996018833319
H	2.07659479571473	-4.12887419424238	-2.29138529818843
H	3.41990425503934	-3.72345605350470	-1.22600831661381
H	1.83295478454482	-3.01371495992178	-0.94344810833468
C	3.84505991104243	-2.58220101753732	-3.75159194323240
H	4.08575002242667	-1.75250561098875	-4.41716893434218
H	4.78475679789774	-2.97201503785178	-3.35760811234307

H	3.39447831666461	-3.37296507272310	-4.35286227070873
C	-2.02094222938007	0.18845604081236	-2.45247298845080
C	-2.73877579059100	1.29067302691663	-1.98483757689802
C	-4.11081233307409	1.13828821249089	-1.82408067390509
H	-4.70220769021109	1.96819183135156	-1.45801105745019
C	-4.73226405507988	-0.06356484902414	-2.11201832028983
H	-5.80105593650070	-0.16380564405611	-1.97561819231789
C	-3.99128897507774	-1.14187718786052	-2.56152010616697
H	-4.48950574085510	-2.08110321338494	-2.76626917964848
C	-2.61670867860123	-1.04060593749881	-2.74034609830905
C	-2.08134066644230	2.60779504186038	-1.63406034400865
H	-1.01179849746157	2.53315305681414	-1.83886887068353
C	-2.22743465057649	2.91190470538508	-0.14288250873896
H	-1.69367552927563	3.82960136750867	0.11070405927089
H	-3.27233764488638	3.05264527027854	0.13918892168128
H	-1.82200023416526	2.10664140305655	0.46997938788018
C	-2.62880022320609	3.74738307331627	-2.49359394441772
H	-2.11195168084046	4.67998364344107	-2.26283604858943
H	-2.49884764069683	3.54733400630267	-3.55778081429168
H	-3.69269139185900	3.91138935207736	-2.31648812347995
C	-1.82388241537684	-2.24876036176111	-3.18910833854179
H	-0.78278806870034	-1.95299148813521	-3.33246158973172
C	-1.84036055242010	-3.33114108801585	-2.11015892226466
H	-1.46587977083642	-2.95054166510646	-1.15995987609727
H	-2.84948286716033	-3.71123907695527	-1.94196995563821
H	-1.21485320152583	-4.17463013068612	-2.40754938332447

C	-2.32636953120262	-2.78984703926285	-4.52695661699248
H	-1.70163995890056	-3.62056281867089	-4.85849466722420
H	-3.34934420653908	-3.16277137067476	-4.45683835197233
H	-2.30573956919676	-2.02463349487411	-5.30358397849339
C	0.43352611594982	0.28824990260655	6.37889420140803
C	2.28168030848348	1.46617225703106	6.98944917843034
C	1.20438179004568	2.23382136777295	7.27460449727464
C	3.71837351358769	1.76641374827329	7.22818966811625
H	4.33066849980355	1.63499462738806	6.33558507794325
H	3.83900999142366	2.80207290428007	7.54088890112623
H	4.14740245413737	1.14169695434869	8.01353236695087
C	1.17418961672420	3.60162843674351	7.85855518977795
H	0.47646219224145	3.68848312733833	8.69141300491921
H	2.15718112613469	3.86610706217589	8.24411826437923
H	0.90369856738486	4.36187793103650	7.12387971804692
C	2.56417366246143	-0.78571921757303	5.83098806691854
H	1.82313327842201	-1.55605652618754	5.61708252569612
C	3.59820573685392	-1.40241440629272	6.76501435833272
H	3.93697010673503	-2.35085808941157	6.34721144010340
H	4.48154751629705	-0.77924866506432	6.89867842678318
H	3.17111526709210	-1.60574282175296	7.74818934564061
C	3.14711168315257	-0.35464421274240	4.49170641434989
H	3.65469191633433	-1.19562520579604	4.01806815491669
H	2.35255708750094	-0.03134895395079	3.81948582531005
H	3.87250839407768	0.45305627545220	4.59733769170078
C	-1.30959746534556	1.87438346294282	7.03244547727968

H	-1.84207678430119	1.09360425517609	6.48980251217024
C	-1.77170769180646	1.80940945909562	8.48253871064749
H	-2.84315883829146	2.00523475307370	8.54776300380565
H	-1.58842848585646	0.81540045367238	8.89045046007838
H	-1.27094783449943	2.54003737799313	9.11889611139713
C	-1.64940814278704	3.19363445915408	6.35244808735362
H	-2.73303984843236	3.29392619496602	6.28097563249571
H	-1.28379766891180	4.06365643640689	6.89693203695440
H	-1.24616312252808	3.22056356718220	5.34056780278646
C	-1.78159447157261	-2.11070207658682	6.83577084657825
C	-2.46711692283065	-3.40808756176016	8.57462375321114
C	-3.57843988756426	-2.94374852661534	7.95533686767519
C	-2.38035209512823	-4.30923543886539	9.75422646127093
H	-1.68296653560812	-3.94506237379714	10.50887124858363
H	-3.35291955568821	-4.38915769292655	10.23681002083207
H	-2.07261393886082	-5.32107602981533	9.48522368458184
C	-5.00039642353258	-3.22120493338091	8.29125374529198
H	-5.60364297922103	-2.31503760409271	8.33394209617377
H	-5.47439165295180	-3.89783078611295	7.57838045966911
H	-5.06815971094528	-3.69351664151129	9.26959226527614
C	0.02663733140723	-3.03514925592832	8.19963765899704
H	0.53548648900137	-2.62518959465125	7.32825691293012
C	0.48092651538156	-4.48185518607092	8.33962517202737
H	1.57064914438588	-4.51986817613002	8.31739557590995
H	0.16542271086669	-4.94544155597283	9.27359422478537
H	0.11167380080367	-5.08658236282058	7.51165207890380

C	0.41017113486882	-2.16642152623392	9.38989470946235
H	0.14188634913354	-1.12812294682647	9.19565485734775
H	-0.07469186278820	-2.48243640948019	10.31464700479795
H	1.48804447189283	-2.20651891227428	9.55471407925404
C	-3.95549169402384	-1.44585514784430	5.92662622493728
H	-3.22696307417208	-1.05250149338465	5.21650707765783
C	-4.88217278177816	-2.36133027731439	5.13959213105999
H	-5.29953994080820	-1.81008279975369	4.29687525434339
H	-4.32952537780583	-3.20938999265295	4.73711490081705
H	-5.71946162447611	-2.73461934823809	5.72903461125389
C	-4.68529965873007	-0.25940393651379	6.54346866387408
H	-5.11039449891275	0.36257885272411	5.75469290487328
H	-5.50595943453780	-0.55737106804850	7.19701496176399
H	-3.99954413529663	0.35714804287515	7.12450047032628

Au(IDipp)(μ-C≡P)Rh(Cp*)(PMe_3) (3)

Rh	0.89196527410419	1.25749909699621	3.34050408793688
Au	0.12594864814672	-0.19135443424845	0.04531206564263
N	1.16775126901074	-0.58077775903793	-2.78031559323887
N	-0.95122993815623	-0.41356534911448	-2.81830672433202
C	0.23776607688562	-0.24352371737693	2.02130676975459
P	0.16943405568852	-1.01059922798000	3.44537454437812
P	3.02340984973349	0.73958367088047	2.99650598802719
C	0.10553069966602	-0.33583915576375	-1.98879591491581
C	0.78335030203527	-0.81300110132747	-4.08250151839710
H	1.49132043040740	-1.02807443637510	-4.86339641643180
C	-0.56103780312125	-0.70832882110625	-4.10625207746687
H	-1.26565844408077	-0.81386520982540	-4.91235040950304
C	2.51529530638456	-0.55505321461582	-2.31535839516386
C	3.19467174973220	0.66525364448270	-2.33571720277412

C	4.50599694428629	0.67445933421745	-1.87443273946317
H	5.06564741604189	1.60158813477971	-1.86454127347805
C	5.10696944704801	-0.48395736729168	-1.41493086148883
H	6.12829341246465	-0.45580346295936	-1.05854857485673
C	4.40429693830365	-1.67533491159940	-1.39837719152204
H	4.88354092165588	-2.57006754979713	-1.02152904946309
C	3.08819250463631	-1.73746796053212	-1.84308930447642
C	2.55070811572447	1.95540092693269	-2.79753474954646
H	1.54479643684848	1.73829882087734	-3.16186217538553
C	2.40278294351923	2.93068218383399	-1.63020348904022
H	1.82867548053246	2.49009588432191	-0.81451720614049
H	1.88616143951781	3.83664828519198	-1.95124883454357
H	3.37439610694907	3.22986711169074	-1.23281755679683
C	3.32147841466429	2.58569728829335	-3.95658975376212
H	4.33067134984035	2.87746856118098	-3.66265619079343
H	2.81136019130148	3.48445564630482	-4.30525148994260
H	3.40841097648879	1.90025089913395	-4.80004465608244
C	2.34035360467667	-3.05221472381128	-1.79550449605830
H	1.30924800663699	-2.88291751243224	-2.11228152786453
C	2.27773948041426	-3.61322946996764	-0.37466159973008
H	3.26771471503685	-3.86154667293923	0.01189879620227
H	1.68491688761793	-4.52886751918350	-0.35949730007988
H	1.81385523223587	-2.90672263830600	0.31377789185915
C	2.95802101600209	-4.06123656354869	-2.76455784087166
H	3.98812633480185	-4.29756267723489	-2.49332619887828
H	2.96520891850662	-3.68451250296864	-3.78806247515547
H	2.39384793741054	-4.99472168012475	-2.75554828819365
C	-2.29340953807111	-0.16574059697968	-2.40414552875797
C	-2.77397233261053	1.14306727176233	-2.48425947471812
C	-4.07800115894096	1.37290618582061	-2.06237843914450
H	-4.48250370864612	2.37650293133546	-2.09606215263575
C	-4.86398117396031	0.33689959626589	-1.58927870671270
H	-5.87686588641732	0.53476121691660	-1.26419146487380

C	-4.35789228708454	-0.94847081256628	-1.52128309447408
H	-4.98398185897335	-1.74559645800154	-1.14101647383032
C	-3.05551762768181	-1.22921252270476	-1.91958842839561
C	-1.92021656899026	2.29726711094042	-2.96367872366339
H	-0.98667585516973	1.90271156611225	-3.37098544086590
C	-1.54723032573376	3.20767629901000	-1.79391363836285
H	-2.43223935883688	3.64246781930687	-1.32657916509332
H	-0.91281866421709	4.02868046992050	-2.13300323400915
H	-1.00367151121580	2.65514785752895	-1.02721956997829
C	-2.59750531704170	3.08140173358459	-4.08642496224514
H	-2.86852072427003	2.43452082577792	-4.92128349752294
H	-1.92785795786186	3.85423504984903	-4.46617851643105
H	-3.50599837687357	3.58096254420299	-3.74744374197134
C	-2.52323123440869	-2.64147220279497	-1.81433582950389
H	-1.46913215133459	-2.64304485769776	-2.09917990488262
C	-2.59612976614490	-3.16112103555031	-0.37874723066925
H	-2.05367495618610	-2.51201503550624	0.30864941896270
H	-2.15507814847767	-4.15694647843871	-0.31572053502575
H	-3.62590853075791	-3.23876716728058	-0.02631640830379
C	-3.25698069497505	-3.57167383244782	-2.78101909684192
H	-4.31699976381746	-3.65206210776762	-2.53487621448282
H	-2.83401479978604	-4.57629773680319	-2.73868269337580
H	-3.18351744939745	-3.22072440928998	-3.81111475969711
C	0.23261123075712	3.40586735815822	2.90682113923195
C	1.03456340380758	3.32573777577390	4.09966294611748
C	0.32067765920719	2.58326649736708	5.08971940786434
C	-0.86962667105886	2.11883965934154	4.47495511779676
C	-0.92785863254952	2.63821433145919	3.13947298482050
C	0.54591619196998	4.22773408537517	1.70101453110135
H	0.02680918118062	3.85622573404743	0.81780924406167
H	1.61116881821703	4.21879877963422	1.46775913194620
H	0.25218892631112	5.27367853095920	1.83407976749243
C	2.25762118011560	4.14331295671247	4.36213566147595

H	1.98807887737611	5.12226161481475	4.76716344210231
H	2.83266469645786	4.32305261171876	3.45438916694328
H	2.92110499687153	3.67080717748298	5.08636401340262
C	0.72155383967295	2.40632023150089	6.51623894462213
H	0.33347389351240	3.20972571205745	7.15002003169150
H	1.80543145927183	2.40013339936064	6.62907424843894
H	0.35616903925304	1.46425884070683	6.92337750643919
C	-1.95310899219973	1.32046794534733	5.11777830178649
H	-1.59095862331722	0.78055306531638	5.99183671509200
H	-2.36345746698880	0.57736924527559	4.43295367150823
H	-2.77791617290974	1.96058990393782	5.44319300524921
C	-2.05970701639045	2.39474646684021	2.19993159457927
H	-1.83506522738687	2.74735954659692	1.19464098383551
H	-2.96882703352009	2.90404751992951	2.53032453095854
H	-2.28554845448011	1.33019121470335	2.11677734190678
C	3.96765048304723	1.83106651333534	1.85471601209923
H	3.92740613368572	2.86411682185219	2.19748003348843
H	3.50810412859706	1.78212524236855	0.86856868211976
H	5.01404622275568	1.53140602577373	1.76990295847222
C	4.10456295112264	0.76871365178301	4.48407412131951
H	5.14154066067459	0.52132503538658	4.24847975688665
H	3.72323811152254	0.05120998084732	5.20958868900773
H	4.07872105411829	1.75443645592863	4.94723032892340
C	3.45035780259691	-0.89644576965711	2.29475289191377
H	3.00490903414057	-0.99733185344503	1.30652288660551
H	3.04680781150898	-1.69301253556294	2.91725055661330
H	4.53270558133245	-1.00728684718332	2.21203609162891

Au(IDipp)(μ-C≡P)[Rh(Cp*)(PMe$_3$)][W(CO)$_5$] (4)

Rh	-0.30735969597299	-1.55028898961181	3.32172977471915
Au	-0.16453943014809	0.05859765219461	0.00178387976887
N	-1.25768634962558	0.40087499703899	-2.81040262847137
N	0.86822071212530	0.33013503202007	-2.86788253825409

C	-0.18399483528047	0.08458701653508	1.98263629738039
P	-0.16505624408947	0.75599175639347	3.45087004567396
P	-2.54455672510353	-1.52444390323753	3.23243671332409
C	-0.17832879905861	0.20057296136344	-2.03159140472733
C	-0.89641336482034	0.66264997514522	-4.11323612378302
H	-1.61986041488314	0.85466976273409	-4.88601043073759
C	0.45131814708794	0.62173402884146	-4.14874139172437
H	1.14308741720561	0.76524933506862	-4.96023617119022
C	-2.59223260771701	0.29865933625897	-2.31774624636554
C	-3.17721203891261	-0.96855878212279	-2.26084286035836
C	-4.45577342344568	-1.05759944928483	-1.72300515679643
H	-4.94266573058209	-2.02222171965710	-1.65110223352676
C	-5.11619110475649	0.07030189202695	-1.26771328807859
H	-6.10886268419874	-0.01980200791108	-0.84658626318831
C	-4.51299340138083	1.31244616375078	-1.34603515137095
H	-5.04091502244842	2.18391679893890	-0.97968762830760
C	-3.23454197295669	1.45524333368425	-1.87445185983469
C	-2.47448495418495	-2.21987796837907	-2.74450451832250
H	-1.49086064624657	-1.94582756744315	-3.13146122531449
C	-2.24404807028503	-3.19983882544450	-1.59566025267477
H	-1.65818771507587	-2.73989144644301	-0.79886227897110
H	-1.70117732928334	-4.07949173012174	-1.94476554836168
H	-3.18480695965775	-3.54663624873902	-1.16431834725654
C	-3.24083069596761	-2.87538611834476	-3.89311812481986
H	-4.23104382853434	-3.21086519762079	-3.58177609727878
H	-2.70004522286256	-3.74860318980660	-4.26032553831494
H	-3.37298396007478	-2.18721314070393	-4.72829947271905
C	-2.59003483818230	2.82205943780720	-1.92836930698024
H	-1.60594375333364	2.72998185277906	-2.39283323852153
C	-2.37323307784105	3.38118751628478	-0.52385682819823
H	-3.31739140037542	3.53696527316893	-0.00047022717270
H	-1.86322136139928	4.34389296060366	-0.56995088620699
H	-1.76520961992571	2.70949821549769	0.08099127607993

C	-3.40884449457647	3.78457139757416	-2.78849807465495
H	-4.38804998398739	3.98435744325176	-2.35087233939400
H	-3.56998238675771	3.39405992586466	-3.79392995319425
H	-2.89361853065954	4.74161663689494	-2.87839511485963
C	2.22204555390513	0.09513658986389	-2.47877999538874
C	2.76139790904800	-1.16869777934423	-2.73746591942442
C	4.07264393524177	-1.39878646556958	-2.33916430773976
H	4.52468243063270	-2.36662584245366	-2.51177981137540
C	4.80746897971204	-0.40857731650092	-1.71055945319531
H	5.82609922510107	-0.60688042080335	-1.40411878813313
C	4.24476014889308	0.83064745081716	-1.46792430251707
H	4.83266905960044	1.59195171151650	-0.97201880494433
C	2.93528164409629	1.11339087868825	-1.84389120706510
C	1.95121246668778	-2.28715379163799	-3.36132676460576
H	1.07037006306053	-1.86263812561583	-3.84806427953728
C	1.45218947648289	-3.24131239230852	-2.27527810964239
H	2.28565713285584	-3.70936137122325	-1.74891469492702
H	0.84108733738959	-4.03514402934551	-2.70893711612146
H	0.84488967036032	-2.71736899742941	-1.53603585294076
C	2.72143586375483	-3.04017570930397	-4.44422575404616
H	3.10429129995228	-2.36473112671984	-5.20958418801335
H	2.06767050538453	-3.76392145550307	-4.93224369793204
H	3.56762300038137	-3.59698475533478	-4.04010121592817
C	2.36125354632061	2.48873134890338	-1.58036032850373
H	1.28958793974620	2.47030374641871	-1.78663309701273
C	2.52261926979231	2.90693727429131	-0.11912606968659
H	2.09796104404036	2.16694657075629	0.55865881972363
H	2.00949778990072	3.85240603000654	0.05987750364802
H	3.56818347626863	3.05389603425757	0.15551678502049
C	2.99213535615275	3.51316125840933	-2.52546438198135
H	4.06682731592858	3.60133579939578	-2.35753344327817
H	2.55181979221628	4.49902414150212	-2.37015794646033
H	2.84291104269646	3.24147884986788	-3.57132609444426

C	0.59585129886341	-3.51681364069147	2.67433827475276
C	0.08955515991358	-3.61078719366492	4.02305128842930
C	0.84351727906521	-2.74155313055982	4.85776088725773
C	1.76748016083182	-2.05352228421098	4.01479774625564
C	1.63546708021159	-2.56057445533094	2.68496866304057
C	0.18986932292147	-4.37864217451201	1.52542506058362
H	0.36439659765171	-3.88024026634877	0.57236457534916
H	-0.86903794477499	-4.63371820426095	1.55986453299516
H	0.74852945309633	-5.31913508320916	1.51104450271241
C	-0.88185788482825	-4.64068105174308	4.49987799401090
H	-0.36865330888571	-5.58170986184067	4.71375716866666
H	-1.64904842294020	-4.86015117335559	3.75825476033190
H	-1.38464857072951	-4.33521332912536	5.41653786248033
C	0.74868792814441	-2.63457757283365	6.34294621410846
H	1.42063983959737	-3.34180385530047	6.83764176750810
H	-0.26002734874827	-2.84032802627067	6.69916313568547
H	1.00839608619550	-1.63728426718435	6.69408927841480
C	2.80332338661340	-1.06703683812428	4.44042647606673
H	2.58376384405231	-0.64535407332779	5.41936916348085
H	2.87595450660829	-0.23550810859449	3.73875574366428
H	3.78941685850642	-1.53502503291136	4.49756653956702
C	2.48406448195153	-2.13517493657342	1.53614087675667
H	2.05582731216256	-2.43301454915305	0.58073868258044
H	3.48255452751054	-2.57496053637122	1.60230298046076
H	2.60497827014698	-1.05189746915784	1.50463652471790
C	-3.33005594386451	-2.80896745532461	2.18055342272174
H	-2.99594739707664	-3.80271377371869	2.47384514100228
H	-3.03356232453788	-2.64441088722364	1.14563619472853
H	-4.41886717804327	-2.77364668089748	2.24586759219161
C	-3.40290496673855	-1.78818378090379	4.83252148063176
H	-4.48807438882059	-1.79642132192388	4.71805088453018
H	-3.12684819745859	-0.99267450099839	5.52320657100321
H	-3.09031261946881	-2.73535606706832	5.27018922196313

C	-3.39114728574428	-0.03077603240653	2.60062354376655
H	-3.05984694026829	0.17818959736794	1.58426286472088
H	-3.14939034270179	0.83670998379221	3.21111622550077
H	-4.47161087927534	-0.18120042728352	2.60470579310221
O	-1.54068024618497	4.45693398071956	2.44834985160135
C	-1.01676179580849	3.88502239356548	3.28558648156814
O	2.75498732192958	3.52786268791654	3.46710024803745
C	1.74965422552901	3.30699606846232	3.95841560208831
O	0.00419259395645	5.56008432499244	6.49325996293568
C	-0.02642144875630	4.58528305586464	5.88881297669978
W	-0.07707693996576	2.88883857434623	4.82132994723282
C	-1.90460262765512	2.37184249812528	5.60470990288915
O	-2.92203829321940	2.06214472601481	6.02276397428999
C	0.85664369232122	1.89037981954426	6.35998156561788
O	1.36996619128485	1.34864780924276	7.22427313073674

4. References

[1] D. W. N. Wilson, S. J. Urwin, E. S. Yang, J. M. Goicoechea, *J. Am. Chem. Soc.* **2021**, *143*, 10367–10373.

[2] S. Bajo, M. G. Alférez, M. M. Alcaide, J. López-Serrano, J. Campos, *Chem. Eur. J.* **2020**, *26*, 16833–16845.

[3] R. A. Brown, G. R. Dobson, *Inorganica Chim. Acta* **1972**, *6*, 65–71.

[4] S. Gaillard, A. M. Z. Slawin, S. P. Nolan, *Chem. Commun.* **2010**, *46*, 2742–2744.

[5] L. Tendera, M. Helm, M. J. Krahfuss, M. W. Kuntze-Fechner, U. Radius, *Chem. Eur. J.* **2021**, *27*, 17849–17861.

[6] CrysAlisPro, Agilent Technologies, Version 1.171.35.8.

[7] a) G. M. Sheldrick in *SHELXL97, Programs for Crystal Structure Analysis* (Release 97-2), Institut für Anorganische Chemie der Universität, Tammanstrasse 4, D-3400 Göttingen, Germany, 1998; b) G. M. Sheldrick, *Acta Crystallogr. Sect. A* **1990**, *46*, 467−473; c) G. M. Sheldrick, *Acta Crystallogr. Sect. C* **2015**, *71*, 3–8.

[8] F. Neese, *Wiley Interdiscip. Rev. Comput. Mol. Sci.* **2012**, *2*, 73–78.

[9] F. Neese, *Wiley Interdiscip. Rev. Comput. Mol. Sci.* **2018**, *8*, 1–6.

[10] F. Neese, F. Wennmohs, U. Becker, C. Riplinger, *J. Chem. Phys.* **2020**, *152*, 224108.

[11] S. Grimme, J. G. Brandenburg, C. Bannwarth, A. Hansen, *J. Chem. Phys.* **2015**, *143*, 054107.

[12] Y. S. Lin, G. De Li, S. P. Mao, J. Da Chai, *J. Chem. Theory Comput.* **2013**, *9*, 263–272.

[13] F. Neese, F. Wennmohs, A. Hansen, U. Becker, *Chem. Phys.* **2009**, *356*, 98–109.

[14] R. Izsák, F. Neese, *J. Chem. Phys.* **2011**, *135*, 144105.

[15] D. A. Pantazis, X. Y. Chen, C. R. Landis, F. Neese, *J. Chem. Theory Comput.* **2008**, *4*, 908–919.

[16] M. Bühl, C. Reimann, D. A. Pantazis, T. Bredow, F. Neese, *J. Chem. Theory Comput.* **2008**, *4*, 1449–1459.

[17] F. Neese, E. F. Valeev, *J. Chem. Theory Comput.* **2011**, *7*, 33–43.

[18] E. D. Glendening, J. K. Badenhoop, A. E. Reed, J. E. Carpenter, J. A. Bohmann, C.

M. Morales, P. Karafiloglou, C. R. Landis, F. Weinhold, **2018**.

[19] M. Mitoraj, A. Michalak, *J. Mol. Model.* **2007**, *13*, 347–355.

[20] L. Zhao, M. von Hopffgarten, D. M. Andrada, G. Frenking, *Wiley Interdiscip. Rev. Comput. Mol. Sci.* **2018**, *8*, e1345.

[21] T. Lu, F. Chen, *J. Comput. Chem.* **2012**, *33*, 580–592.

[22] T. K. Ghanty, *J. Chem. Phys.* **2005**, *123*, 241101.

[23] B. Kiran, X. Li, H. J. Zhai, L. S. Wang, *J. Chem. Phys.* **2006**, *125*, 133204.

[24] X. Li, B. Kiran, L. S. Wang, *J. Phys. Chem. A* **2005**, *109*, 4366–4374.

[25] M. Megha, C. Kamal, K. Mondal, T. K. Ghanty, A. Banerjee, *J. Phys. Chem. A* **2019**, *123*, 1973–1982.
